# Ionic weaving network enables mechanically robust smart hydrogels with anti-swelling and anti-freezing capabilities

**DOI:** 10.1038/s41467-026-76165-4

**Published:** 2026-07-29

**Authors:** Mengyu Gan, Chenxi Yang, Xiaozheng Su, Jiahao Kang, Ye Lin, Jiale Hong, Piaopiao Zhou, Xiaolin Lyu, Zhigang Zou

**Affiliations:** 1 State Key Laboratory of Green and Efficient Development of Phosphorus Resources, College of Materials Science and Engineering, Fuzhou University, Fuzhou, Fujian China; 2 Department of Lung Transplantation, Fujian Medical University Union Hospital, Fuzhou, Fujian China; 3 Key Laboratory of Cardio-Thoracic Surgery, Fujian Medical University, Fuzhou, Fujian China; 4 Eco-materials and Renewable Energy Research Center, College of Engineering and Applied Sciences, Nanjing University, Nanjing, China

**Keywords:** Mechanical properties, Mechanical properties, Gels and hydrogels

## Abstract

Stretchable hydrogels hold great promise in cutting-edge fields such as flexible sensing and soft robotics. However, the poor mechanical properties and environmental intolerance restrict their practical applications in intelligent integrated systems. Herein, we construct ionic weaving hydrogels (IWHs) by pre-arranging polymer chains through force-induced restrictive drying, followed by the introduction of metal ions to form a 3D topological network with uniformly distributed dynamic coordination. This design dissipates energy through the synchronous dissociation of massive coordination bonds, yielding the hydrogel with high strength, high modulus, and excellent toughness. Additionally, the strong topological constraints impart excellent anti-swelling and anti-freezing capabilities, as well as dehydration-rehydration activation characteristics. Consequently, the IWH enables not only stable underwater sensing but also integrated actuation-sensing functions through solvent stimulation. Moreover, when processed by 3D printing, it achieves spatiotemporally programmable actuation while simultaneously monitoring the response process in real time. This work demonstrates an intelligent 4D printing platform that seamlessly integrates complex structures, actuation, and self-sensing, thereby opening an avenue for designing next-generation multifunctional smart hydrogels and soft material systems.

## Abstract (web summary)

Stretchable hydrogels have a range of potential applications, but mechanical properties limit current practical use. Here, the authors report the development of weaving hydrogels by prearranging of polymer chains with metals ions, to give energy dissipation and therefore improved properties.

## Introduction

Hydrogels demonstrate tremendous potential in cutting-edge fields such as flexible electronics, wearable devices, and soft robotics^1–5^. For instance, highly sensitive flexible sensors based on hydrogels can monitor human motion in real-time^6–10^, while the reversible deformation of hydrogels in response to external stimuli (e.g., temperature, humidity, solvents) enables them to function as artificial muscles or soft actuators, achieving complex movements like grasping and peristalsis^11–16^. However, the poor mechanical properties (e.g., low strength, limited stretchability) and environmental instability (e.g., swelling underwater, freezing at low temperatures) of most hydrogels severely limit their practical application and stable operation in a wide range of environments.

The inherent weak mechanical properties of traditional hydrogels stem from the weak interactions within polymer networks, resulting in poor energy dissipation capabilities^17–19^. Therefore, researchers have developed various strategies to toughen hydrogels through energy dissipation mechanisms in recent years, such as utilizing a second network or noncovalent interactions as sacrificial bonds, and introducing nanomaterials like MXene, graphene, and liquid metals to inhibit crack propagation^20–24^. Additionally, phase-separated regions can be further constructed within hydrogels through hydrophobic interactions, salting-out effects, and mechanical training to enhance their strength^25–30^. Nevertheless, these hydrogels still exhibit environmental intolerance. They tend to swell via hydration of hydrophilic groups underwater or become brittle due to freezing of water molecules at low temperatures, leading to deterioration of mechanical properties and degradation of sensing and actuation performance^31–33^. The environmental stability of hydrogels is influenced by multiple factors, including water state, osmotic pressure, ion leaching, solvent exchange, network charge density, polymer-water interactions, and structural heterogeneity. These factors make hydrogels susceptible to swelling by external water molecules or freezing of internal water molecules^34,35^. Meanwhile, hydrogels often experience irreversible degradation in mechanical properties upon rehydration after dehydration, further compromising their functional reliability in practical applications^36,37^. In addition, although advanced manufacturing techniques like 3D printing can conveniently construct hydrogel actuators and even artificial muscles with complex structures, these systems typically lack intrinsic sensing capabilities^38,39^. They cannot perceive their own deformation in real-time while executing actions, like muscles do, which significantly limits their autonomy and intelligence. Although external sensors can be integrated into hydrogels to achieve sensing functions, this approach increases system complexity and introduces feedback latency^40^. Therefore, synergistically enhancing the mechanical robustness and environmental tolerance of hydrogels for intelligent sensing and actuation systems remains an urgent yet challenging task.

**Fig. 1:**
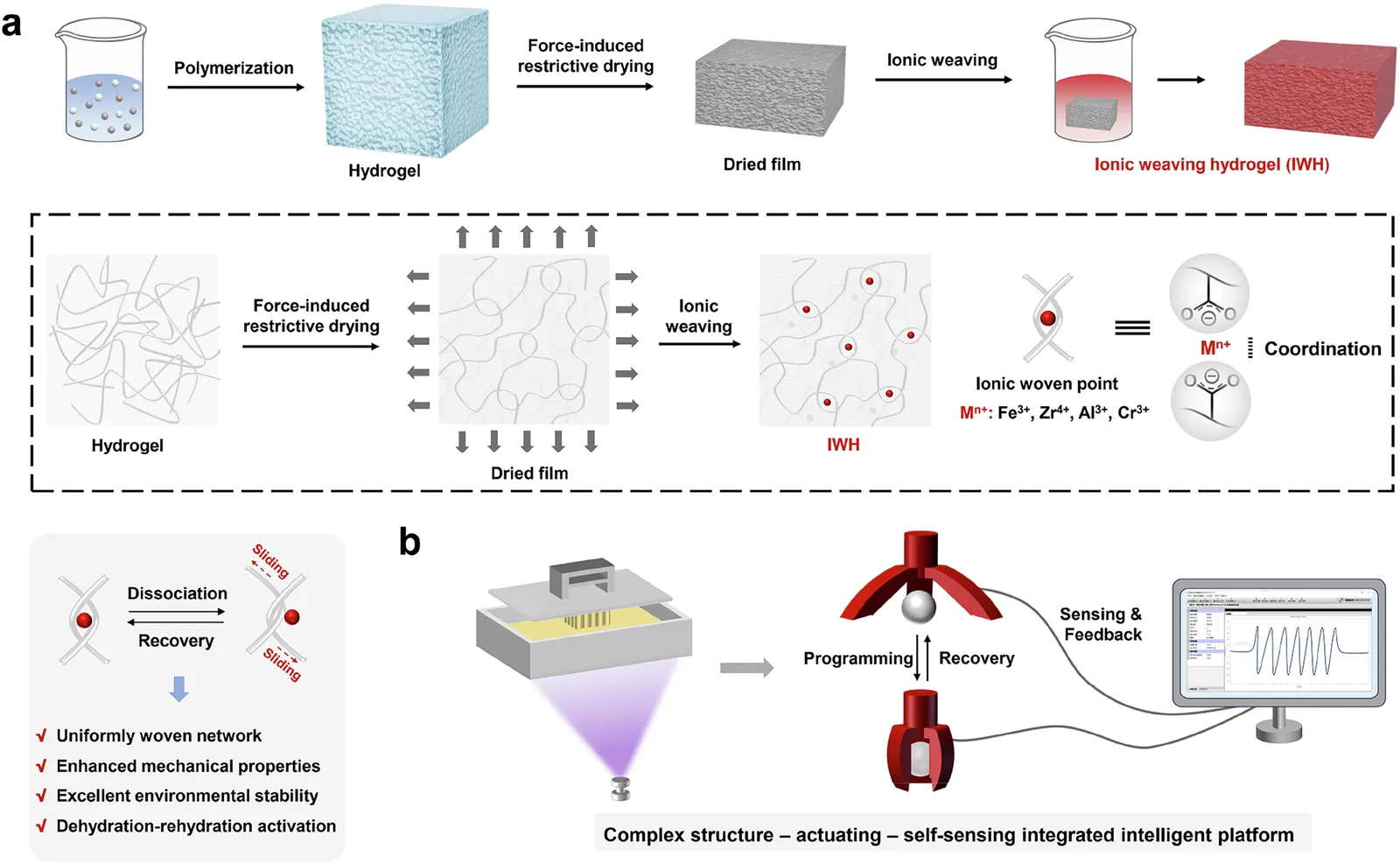
Design of IWH and integrated intelligent platform. **a** Schematic illustration of the preparation process and structure of IWH. **b** Schematic illustration of the intelligent platform integrating complex structure, actuation, and sensing functions.

Herein, we ingeniously design a kind of ion-conductive hydrogel with favorable mechanical properties and environmental stability by employing an ionic weaving strategy. The core design of ionic weaving hydrogels (IWHs) lies in a pre-treatment step involving restricted drying, which induces dense pre-alignment of polymer chains. Subsequently, metal ions are introduced to form interlaced dynamic coordination bonds among the pre-organized polymer chains, thereby constructing a uniform 3D topological network that pervades the entire hydrogel (Fig. 1a). This structural design exerts robust topological constraints through uniform ion coordination, enabling tight entanglement of polymer chains. During deformation, energy is dissipated via the synchronous dissociation of numerous coordination bonds within the polymer network, resulting in IWH with excellent mechanical properties. Moreover, the topological constraint suppresses the movement of polymer chain segments and restricts water molecules, conferring IWH with anti-swelling and intrinsic anti-freezing capabilities. Furthermore, even after dehydration, IWH can be restored to its original state through water activation while retaining the original performance. Leveraging the ion-conductive properties, IWH can achieve stable flexible sensing capabilities underwater and realize integrated actuation-sensing functions in biomimetic actuators through solvent-induced shape memory effects. Simultaneously, the complex 3D-printed structures of IWH can not only exhibit programmable responses in both spatial and temporal dimensions upon solvent stimulation but also spontaneously perceive the shape-changing process through electrical signals, further achieving seamless integration among actuators, complex structures, and self-sensing (Fig. 1b). This elucidates the intelligent 4D printing capabilities of IWH. This work opens up a design avenue for constructing high-performance hydrogels and next-generation intelligent soft materials.

## Results

### Design and structural characterization of IWH

**Fig. 2:**
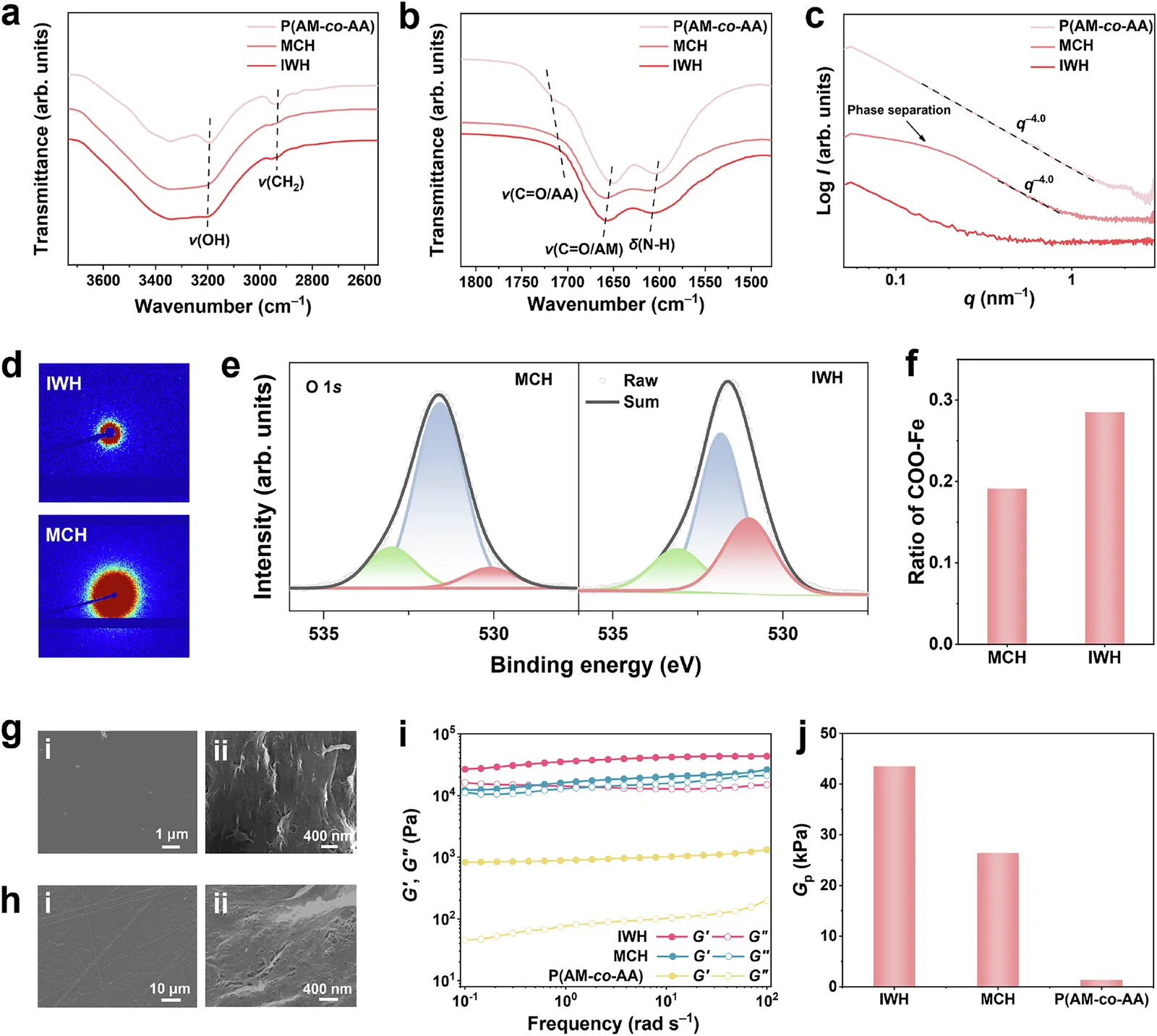
Structural characterization of IWH. **a**, **b** Infrared spectra of P(AM-*co*-AA), MCH, and IWH. **c** 1D SAXS curves of P(AM-*co*-AA), MCH, and IWH. **d** 2D SAXS images of IWH and MCH. **e** O 1*s* XPS spectra of IWH and MCH. Green: C-O, blue: C=O, red: COO^–^−Fe^3+^. **f** Peak area ratio of Fe^3+^-COO^–^ in IWH and MCH. SEM images of the surface (i) and cross-section (ii) of IWH (**g**) and MCH (**h**). **i** Rheological frequency-sweeping curves of P(AM-*co*-AA), MCH, and IWH. **j** Storage modulus at 10 rad s^–1^ of P(AM-*co*-AA), MCH, and IWH. The bar chart column in (**f** and **j**) represents a single measurement.

To elucidate our design strategy, the polymer network of the model hydrogel was obtained by copolymerizing acrylamide (AM) and acrylic acid (AA), with the metal ions supplied by FeCl_3_ solution. AM and AA, as common monomers, offer advantages such as low cost and easy availability. The two monomers, initiator, and cross-linker were dissolved in water, and the original poly(acrylamide-*co*-acrylic acid) (P(AM-*co*-AA) hydrogel was polymerized under UV light (Fig. 1a). Subsequently, the hydrogel was placed on a flat substrate. After the four edges were fixed, force-induced restricted drying was carried out to induce dense re-alignment of the polymer chain segments. The restrictedly dried film was then immersed in an FeCl_3_ solution for ionic weaving through metal-coordination interactions. Finally, it was immersed in deionized water to remove excess Fe^3+^, resulting in the IWH. A series of hydrogels were prepared and named IWH-*m*-*n*, where *m* is the mass ratio of AA to AM, and *n* M is the concentration of the FeCl_3_ solution used for immersion. The results show that the strengthened metal-coordination interactions render the IWH darker in color and lower in water content with increasing AA content and Fe^3+^ concentration (Supplementary Fig. 1). Meanwhile, a traditional metal-coordination hydrogel (MCH) was prepared using a traditional soaking method by directly immersing the P(AM-*co*-AA) hydrogel in FeCl_3_ solution without the restricted drying step as a control. Unlike the MCH, which undergoes significant dimensional shrinkage when soaked in a salt solution, the IWH shows almost no noticeable changes in size and area during the drying and soaking processes (Supplementary Fig. 2). This suggests that there are substantial differences in the internal interactions and structures between the hydrogels obtained by our ionic weaving method and the traditional soaking method. First, Fourier-transform infrared (FTIR) spectroscopy was used to investigate the effects of different preparation methods on the internal interactions within the hydrogels. The intermolecular interactions among polymer chains in the P(AM-*co*-AA) hydrogel are primarily composed of hydrogen bonds formed between carboxyl groups. After introducing Fe^3+^, the carboxyl groups in both the IWH and MCH coordinate with Fe^3+^ to form metal-coordination interactions, leading to a decrease in the peak intensity of *ν*(OH) and a redshift of *ν*(C = O/AA) (Fig. 2a, b). Moreover, the formation of metal-coordination interactions also modifies the packing arrangement of the polymer chains, resulting in blueshifts of the *δ*(C-H) and *ν*(CH_2_) of the methylene groups on the main chain, as well as the amide I and amide II bands (Fig. 2a, b, and Supplementary Fig. 3).

Subsequently, small-angle X-ray scattering (SAXS) measurements were performed on the P(AM-*co*-AA) hydrogel, IWH, and MCH. The SAXS results reveal that the P(AM-*co*-AA) hydrogel exhibits a power-law exponent of –4.0 in the *q*-value range of 0.1–1 nm^–1^, indicating that the primary scattering from molecular chains originates from hydrogen-bonded clusters formed between polymer chains (Fig. 2c). For the MCH, the power-law exponent in the high *q* region of 0.4–1 nm^–1^ remains –4.0 after the introduction of Fe^3+^, suggesting the continued presence of hydrogen-bond clusters formed by polymer chains. Meanwhile, the peak observed near *q* = 0.2 nm^–1^ in the MCH arises from metal polynuclear clusters formed by carboxylic acid bridging, with an averaged spacing of ~31 nm^20,41^. Therefore, direct soaking in a salt solution generates an inhomogeneous distribution of metal ions in the MCH, resulting in the simultaneous formation of hydrogen-bond clusters and metal clusters. However, for the IWH, the scattering from hydrogen-bond clusters in the high *q* region of 0.4–1 nm^–1^ completely disappears, and there are no metal clusters caused by metal-coordination interactions. Thus, the coordination between Fe^3+^ and carboxyl groups can achieve a uniform distribution in the IWH by introducing the restricted drying step, enabling the close entanglement of polymer chains through ions and the formation of a uniform dynamic coordination network (Fig. 1a). The correlation between polymer chains can be further analyzed through wide-angle X-ray scattering (WAXS). The formation of metal-coordination interactions can enhance the electron cloud density contrast between the polymer backbones, resulting in the emergence of a peak near 3.7 nm⁻^1^ (Supplementary Fig. 4). Moreover, compared to the MCH, the ionic weaving network renders the polymer network in the IWH more ordered, leading to a higher peak intensity. The 2D SAXS results further confirm that the uniformly coordinated network in the IWH exhibits a distinct isotropic structure, while the scattering ring of the MCH is slightly elliptical, indicating a certain degree of anisotropy due to the unevenly distributed hydrogen-bond clusters and metal clusters (Fig. 2d). Subsequently, we further explored the coordination between Fe^3+^ and carboxyl groups within the hydrogels using X-ray photoelectron spectroscopy (XPS). The O 1*s* spectra reveal the emergence of a COO^–^−Fe^3+^ coordination peak in both the IWH and the MCH (Fig. 2e). By analyzing the peak area ratio of COO^–^−Fe^3+^, we find that the content of coordinated Fe^3+^ in the IWH is considerably higher than that in the MCH, indicating that the number of coordinated carboxyl groups in the IWH is much greater than that in the MCH (Fig. 2f). Furthermore, XPS results show that the peak area ratio of COO^–^−Fe^3+^ is similar on the surface and cross-section of IWH (Supplementary Fig. 5a, b). Moreover, energy-dispersive spectroscopy of SEM further indicates that the Fe element is uniformly distributed along the thickness direction of the hydrogel, without aggregation or concentration gradient (Supplementary Fig. 5c).

The differences in internal interactions also lead to significant differences in the microstructures of the hydrogels. The microscopic morphology was observed by scanning electron microscopy (SEM). The P(AM-*co*-AA) hydrogel exhibits a porous structure (Supplementary Fig. 6). After the introduction of Fe^3+^, the uniform ionic weaving approach enables the polymer porous network to undergo homogeneous shrinkage, resulting in the formation of a dense, non-porous architecture in the IWH (Fig. 2g). However, the inhomogeneous structure gives rise to both closed-pore and open-pore structures in the MCH due to the simultaneous presence of metal clusters and hydrogen-bond clusters (Fig. 2h). Therefore, compared to the MCH, the IWH possesses enhanced internal interactions and a more stable structure, enabling it to better resist external forces. The frequency-sweeping rheological results indicate that the IWH has a higher shear modulus compared to the MCH (Fig. 2i). Its storage modulus at 10 rad s^−^^1^ is almost twice that of the MCH (Fig. 2j). In summary, the ionic weaving strategy can regulate the distribution and coordination mode of Fe^3+^ through the synergistic effects of re-alignment of polymer chain segments and dynamic coordination, constructing a dense and uniform topological network structure. This can effectively enhance the mechanical properties and environmental stability of the hydrogel, which will be discussed in detail subsequently.

### Mechanical properties, anti-swelling, and anti-freezing

**Fig. 3:**
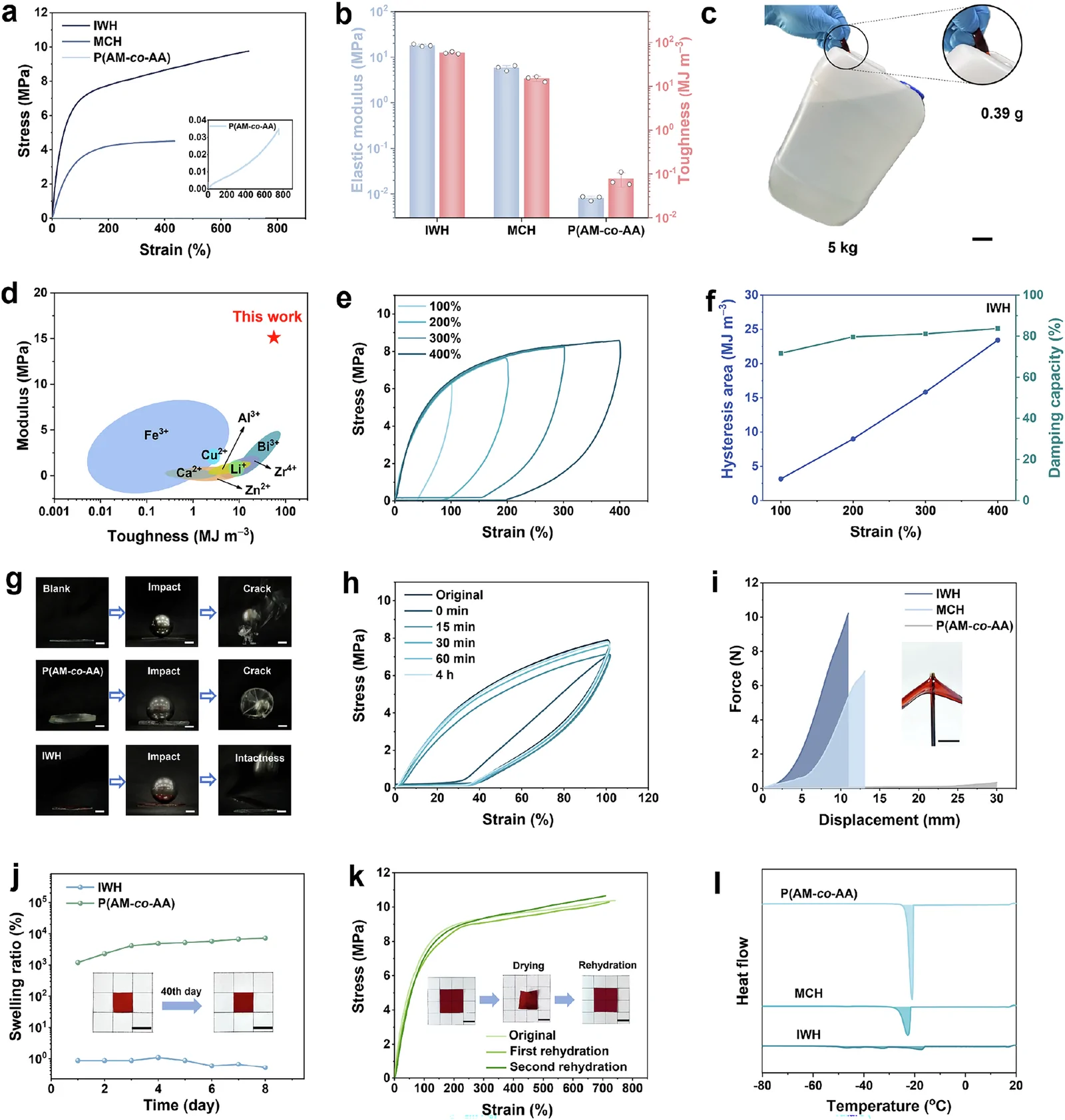
Mechanical properties of hydrogels. Stress-strain curves of different hydrogels (**a**) and the corresponding Young’s modulus and toughness (**b**). **c** Photograph of IWH lifting a 5 kg bucket. Scale bar: 20 cm. **d** Comparison of Young’s modulus and toughness between the IWH in this study and different metal-coordination hydrogels. Loading-unloading curves of IWH at different strains (**e**) and the corresponding dissipated energy and damping capacity (**f**). **g** Photographs of an iron ball falling from a height of 50 cm onto a glass sheet. Scale bar: 1 cm. **h** Tensile stress-strain curves of IWH during repeated loading-unloading cycles at a strain of 100% with different waiting times. **i** Puncture force-displacement curves of IWH, MCH, and P(AM-*co*-AA) hydrogels. Scale bar: 5 cm. **j** Swelling curves of IWH and P(AM-*co*-AA) hydrogels in water. Scale bar: 1 cm. **k** Stress-strain curves of IWH after drying-rehydration. Scale bar: 1 cm. **l** DSC curves of IWH, MCH, and P(AM-*co*-AA). Data in (**b**) are reported as their means ± SDs from *n* = 3 independent samples.

The dynamic ionic weaving point can provide robust linkages between polymer chain segments, effectively dissipating energy during deformation and imparting excellent mechanical properties to IWH. First, we characterized the mechanical properties of the dried sample to rule out processing artifacts caused by restricted drying, which was brittle and rigid with poor stretchability and fractured immediately upon stretching (Supplementary Fig. 7). Subsequently, we investigated the effects of monomer ratio and metal ion concentration on the mechanical properties of the hydrogel. As the proportion of AA increases, the modulus and strength of IWH rapidly improve while its stretchability decreases due to the strengthened topological cross-linking induced by increased coordination density (Supplementary Fig. 8a, b). Similarly, the modulus and strength of IWH also increase with an increase in the Fe^3+^ concentration, while its stretchability decreases (Supplementary Fig. 8c, d). Overall, when the AA content is 20% and the Fe^3+^ concentration is 0.1 M, the IWH exhibits optimal comprehensive mechanical properties, with a strength, modulus, and toughness of 9.2 MPa, 18.2 MPa, and 57.4 MJ m^−^^3^, respectively. Therefore, we select IWH with this component ratio for subsequent research and compare it with the MCH to elucidate the effectiveness of the ionic weaving network for metal-coordination hydrogels. The original P(AM-*co*-AA) hydrogel is relatively soft and brittle (Fig. 3a, b). In contrast, the uniform coordination mode of IWH can effectively enhance the mechanical properties of the hydrogel, outperforming the MCH significantly. We further explored the influence of different environmental conditions during restricted drying on the mechanical properties of IWH. Similar mechanical properties are observed under different temperatures and humidities, revealing that the improvement in the mechanical properties of IWH is primarily dependent on the restricted drying step and is less influenced by environmental parameters (Supplementary Fig. 9). IWH can not only sustain various deformations such as stretching and kinking (Supplementary Fig. 10) but also lift a weight over 12,000 times its own (Fig. 3c). This indicates that IWH integrates flexibility and high load-bearing capacity. Thus, IWH achieves an effective combination of high strength, high stiffness, and high toughness, demonstrating significantly enhanced mechanical properties. The mechanical properties of IWH exceed those of most metal coordination hydrogels, which reveals the effectiveness of the ionic weaving strategy (Fig. 3d and Supplementary Fig. 11).

Then, we characterized the energy dissipation of the IWH through cyclic stress-strain curves. As the strain increases from 100% to 400%, the dissipated energy of IWH rapidly rises from 3.2 MJ m^−^^3^ to 23.2 MJ m^−^^3^, with a damping capacity exceeding 80% (Fig. 3e, f). Compared with the MCH, IWH exhibits dramatically improved dissipated energy and damping capacity (Supplementary Fig. 12). The excellent energy dissipation capability of IWH is mainly attributed to the fact that the metal-coordination motifs in the uniform coordination network can undergo dissociation and effectively disperse stress during deformation. Furthermore, the IWH also demonstrates excellent impact resistance. IWH can effectively reduce the impact force by 33%, and its impact resistance far surpasses that of the MCH, P(AM-*co*-AA) hydrogel, and conventional damping materials such as PDMS (Supplementary Fig. 13). Photographic demonstrations also indicate that the protection provided by IWH can prevent the iron ball from shattering the glass sheet when an iron ball falls from a height of 50 cm (Fig. 3g). In contrast, the glass without protection and the glass under the protection of the P(AM-*co*-AA) hydrogel show obvious fragmentation. Moreover, the reversibility of the coordination bonds provides IWH with good self-recovery ability. After 24 h of recovery, its length is consistent with the original state (Supplementary Fig. 14). Through cyclic stress-strain curves, it can be found that the modulus and dissipated energy can be fully recovered after a waiting time of 4 h (Fig. 3h and Supplementary Fig. 15). Besides, the ionic weaving approach endows the IWH with good puncture resistance and crack propagation resistance. Puncture experiments demonstrate that the puncture force and puncture energy of IWH are considerably higher than those of the MCH and P(AM-*co*-AA) hydrogel, allowing it to better resist the impact of sharp objects (Fig. 3i and Supplementary Fig. 16). Meanwhile, the fracture energy and fractocohesive length of IWH (9.8 kJ m^−^^2^, 2.2 mm) are also higher than those of the MCH sample (4.9 kJ m^−^^2^, 0.47 mm) and P(AM-*co*-AA) hydrogel (0.08 kJ m^−^^2^, 1.9 mm) (Supplementary Fig. 17).

In addition to its good mechanical properties, the ionic weaving network gives the IWH excellent anti-swelling capability, which is conducive to the application of the hydrogel in complex and dynamic environments. Following immersion in deionized water for one week, the swelling ratio of IWH is only 0.5%, which is much lower than that of P(AM-*co*-AA) hydrogel (7225%) (Fig. 3j). Changing the thickness of IWH does not affect its anti-swelling capability (Supplementary Fig. 18). Even after being soaked in water for 40 days, IWH can still retain its original dimensions (Supplementary Fig. 19). Moreover, the leaching amount of Fe^3+^ during the swelling test is low, confirming the high stability of the coordination network (Supplementary Fig. 20). Through tensile tests, it is found that IWH still exhibits excellent mechanical properties after being soaked in water for 2–6 days (Supplementary Fig. 21). It is worth mentioning that IWH also has reversible drying-rehydration capability. When IWH is dried and then placed back into water, it can rapidly revert to its original state, and the mechanical properties remain stable during multiple drying-hydration cycles (Fig. 3k).

Furthermore, traditional hydrogels are susceptible to structural damage at low temperatures due to water freezing, which severely restricts their applications under cryogenic conditions. However, the uniformity of the ionic weaving network can effectively suppress the formation of ice crystals, thereby enabling the IWH to exhibit good anti-freezing capability. As displayed in Fig. 3l, the results of differential scanning calorimetry (DSC) measurements reveal that both P(AM-*co*-AA) hydrogel and MCH exhibit prominent crystallization peaks, while the crystallization peak in IWH is greatly suppressed. This shows that the strong topological confinement of the ionic weaving network can attenuate the hydrogen-bonding interactions among water molecules, allowing only trace amounts of ice crystals to form. Therefore, IWH can always retain structural integrity when exposed to low temperatures (−20 °C and −196 °C) (Supplementary Fig. 22). In contrast, the P(AM-*co*-AA) hydrogel and MCH undergo fracture after being placed at −196 °C due to structural damage caused by water freezing. Compared with the recently reported environmentally stable hydrogels, our IWH demonstrates enhanced anti-swelling and anti-freezing capabilities (Supplementary Fig. 23).

### Insights into the structure-property relationship

IWH exhibits excellent mechanical properties and network stability due to the formation of an ionic weaving network. Therefore, we conduct an in-depth exploration of its structure-property relationship to further elucidate the mechanism of the ionic weaving network on the properties. We reveal the dynamic characteristics of the ionic weaving network through a series of experiments by comparing the IWH and MCH.

**Fig. 4:**
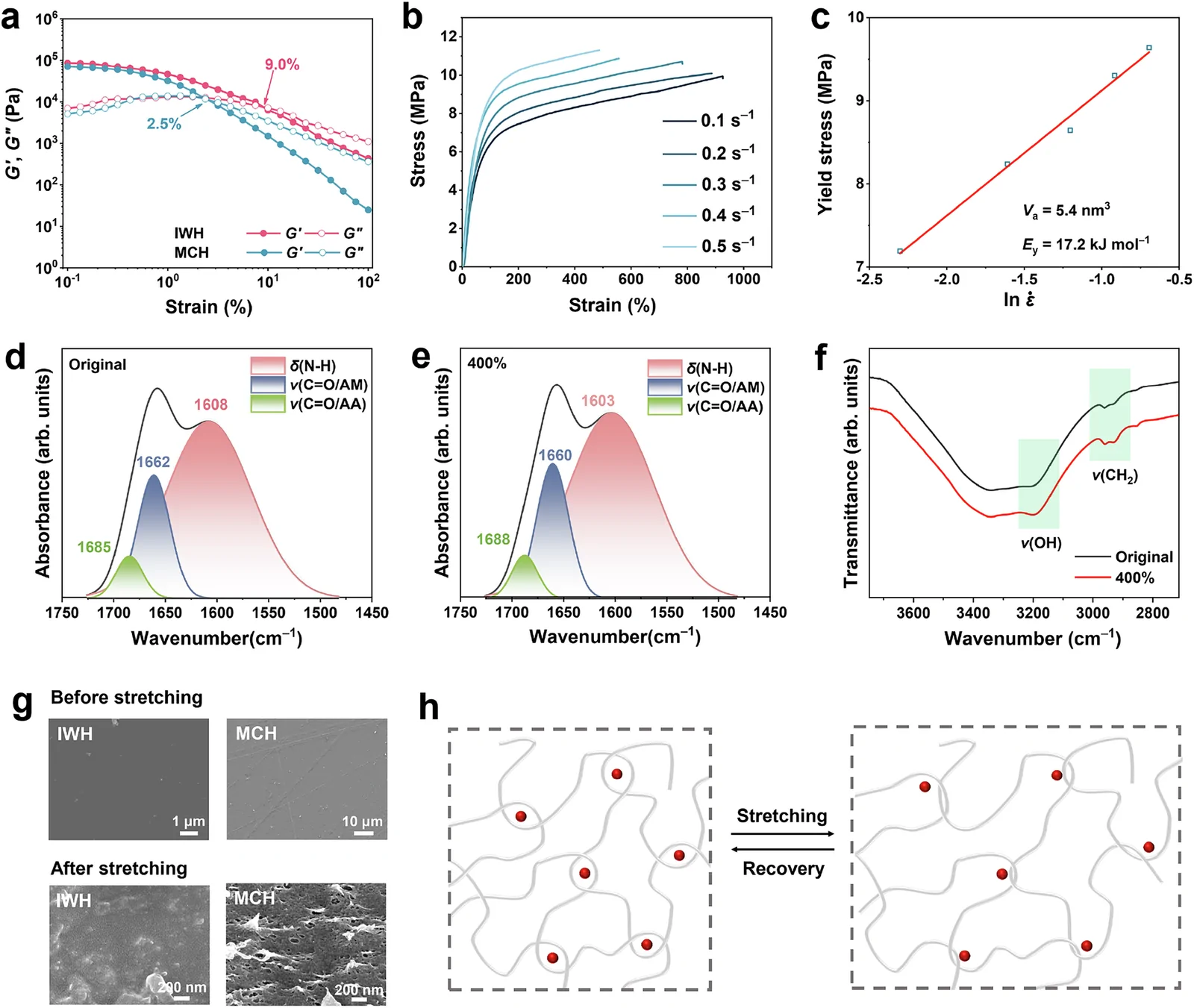
Ionic weaving mechanism of IWH. **a** Strain-sweeping rheological curves of IWH and MCH. **b** Stress-strain curves of IWH at different stretching rates. **c** Fitted curve of the yield stress versus the deformation rate. **d**–**f** Infrared results of IWH before and after stretching. **g** SEM images of IWH and MCH before and after stretching. **h** Schematic illustration of chain-segment movement in IWH before and after stretching.

The strain-sweeping rheological results show that the transition point of IWH (9.0%) is larger compared with that of MCH (2.5%) (Fig. 4a). This result indicates that the coordination network in IWH can effectively restrict the free movement of chain segments, making the polymer network more stable. Furthermore, the good reversibility of the ionic coordination network in IWH is confirmed through step-strain rheology with alternating small and large strains (Supplementary Fig. 24). At 1% strain, the stable coordination network results in a storage modulus (*G*’) higher than the loss modulus (*G*”). When the strain is increased to 100%, the dynamic dissociation of the coordination network leads to the *G*” exceeding the *G*’. Subsequently, when the strain is switched back to 1%, both the *G*’ and *G*” rapidly return to their initial values owing to the reformation of the coordination network. Meanwhile, the stress-strain curves of IWH exhibit rate-dependence at different stretching rates, further demonstrating the dynamic characteristics of the ionic weaving network (Fig. 4b). Moreover, the yield stress and tensile strength of the material gradually increase as the strain rate increases (from 0.1 s^−1^ to 0.5 s^−1^), while the fracture strain gradually decreases, indicating enhanced restriction of chain-segment movement at faster stretching rates. There is a good linear relationship between the yield stress and the natural logarithm of the deformation rate (ln $\dot{\varepsilon }$), which is consistent with the Eyring model for mechanically induced dissociation of noncovalent bonds. The fitted activation volume and activation energy are 5.4 nm^3^ and 17.2 kJ mol^−1^, respectively (Fig. 4c). This suggests that IWH must overcome a relatively high energy barrier within a small space during yielding, reflecting the improved network stability and enhanced performance caused by the ionic weaving network. After yielding, the polymer chains further slide and align under the confinement of the coordination network, entering the plastic deformation region and exhibiting strain-stiffening. This can be attributed to the gradual unfolding of the coordination network until it completely fractures. In contrast, the activation volume and activation energy of the MCH are 7.2 nm^3^ and 10.9 kJ mol^−1^, respectively (Supplementary Fig. 25). Its chain segments have a larger moving space and a lower energy barrier, indicating that the polymer network in the MCH has a weaker confining effect, resulting in relatively lower energy dissipation capacity and mechanical performance.

Furthermore, the molecular-scale changes in IWH during stretching were monitored via FTIR. When the IWH is stretched to 400% strain, the entire ionic weaving network dissipates energy through the synchronous dissociation of coordination bonds. As a result, *ν*(C = O/AA) undergoes a corresponding blue shift (Fig. 4d, e). Simultaneously, the exposed carboxyl groups re-associate to form hydrogen bonds owing to the dissociation of metal ions, so *ν*(O-H) is re-enhanced (Fig. 4f). Moreover, the network structure of the ionic weaving network does not change significantly during deformation. Therefore, the positions of *δ*(C-H) and *ν*(CH_2_) in the polymer chains remain almost unchanged (Fig. 4f and Supplementary Fig. 26). However, the characteristic peaks of the MCH show almost no change before and after stretching (Supplementary Fig. 27). This is likely because its non-weaving nature causes it to dissipate energy mainly through the fracture of polymer chains rather than the coordinated dissociation of the entire network. SEM results also indicate that IWH exhibits uniformly distributed fine microcracks after stretching owing to the synergistic dissociation of the ionic weaving network during stretching, suggesting that stress can be uniformly transferred and efficiently dissipated via dynamic cross-linking sites (Fig. 4g). However, the MCH shows a large number of irregular pores along with the fine microcracks, and the pore size distribution is heterogeneous, reflecting its tendency to experience stress concentration during stretching, leading to local chain-segment fracture and structural damage (Fig. 4g).

Based on the above analysis, we describe the potential mechanism underlying the excellent properties of IWH as follows. After restricted drying, the polymer chains can undergo close pre-alignment, and the subsequently introduced metal ions enable the formation of a dense topological network structure. Because of the uniform cross-linking characteristics of the polymer network, it exhibits good structural stability without undergoing swelling underwater, and can always maintain its original structure after drying-rehydration cycles. Meanwhile, the topological network structure exerts a strong restrictive effect on the polymer chains, significantly reducing the content of free water and thus endowing it with excellent anti-freezing performance. When subjected to force, the entire network of IWH can deform synchronously, achieving stress dispersion and dissipating a large amount of energy through the dissociation of the coordination network, which confers high stretchability and high toughness to IWH (Fig. 4h). In addition, the dense network can effectively constrain the sliding range of polymer chain segments and preserve the network integrity, thereby enhancing its stiffness and strength. Furthermore, it should be noted that although we have confirmed the dynamic nature of the coordination network through deformation-induced dissociation and reformation, temperature‑dependent coordination dynamics were not directly investigated in this study. Therefore, compared with hydrogen-bonded crosslinking hydrogels and traditional metal-coordination hydrogels, the uniform coordination network and synergistic deformation-dissociation in IWH notably improve its macroscopic properties, offering an approach for the design and regulation of high-performance hydrogels.

### Strategy universality

To demonstrate the broad applicability of the ionic weaving strategy in optimizing the mechanical properties of hydrogels, we prepared a series of IWH materials with high mechanical properties by varying the types of monomers and metal ions. First, we substituted the AA monomer with methacrylic acid (MAA), itaconic acid (IA), and maleic acid (MA), all of which bear abundant carboxyl groups. The resulting hydrogels were named IWH-X (where X represents different alternative monomers). IWH-X exhibits higher strength, modulus, and toughness compared with the control samples (Supplementary Fig. 28). Furthermore, in terms of metal ion selection, we replaced Fe^3+^ with three other metal salts (ZrCl_4_, AlCl_3_, and CrCl_3_) to form IWH. The resulting hydrogels were named IWH-Y (where Y represents different metal ions). The results indicate that IWH-Y also possesses higher strength and toughness compared with the control samples (Supplementary Fig. 29). Therefore, the ionic weaving network achieved through the synergy of restricted drying and metal ion coordination is a versatile strategy to prepare high-performance hydrogels. This strategy provides a scalable and modular design paradigm for constructing high-performance hydrogels suitable for complex mechanical environments such as wearable devices and soft robotics.

### Electrical properties and sensing performance

**Fig. 5:**
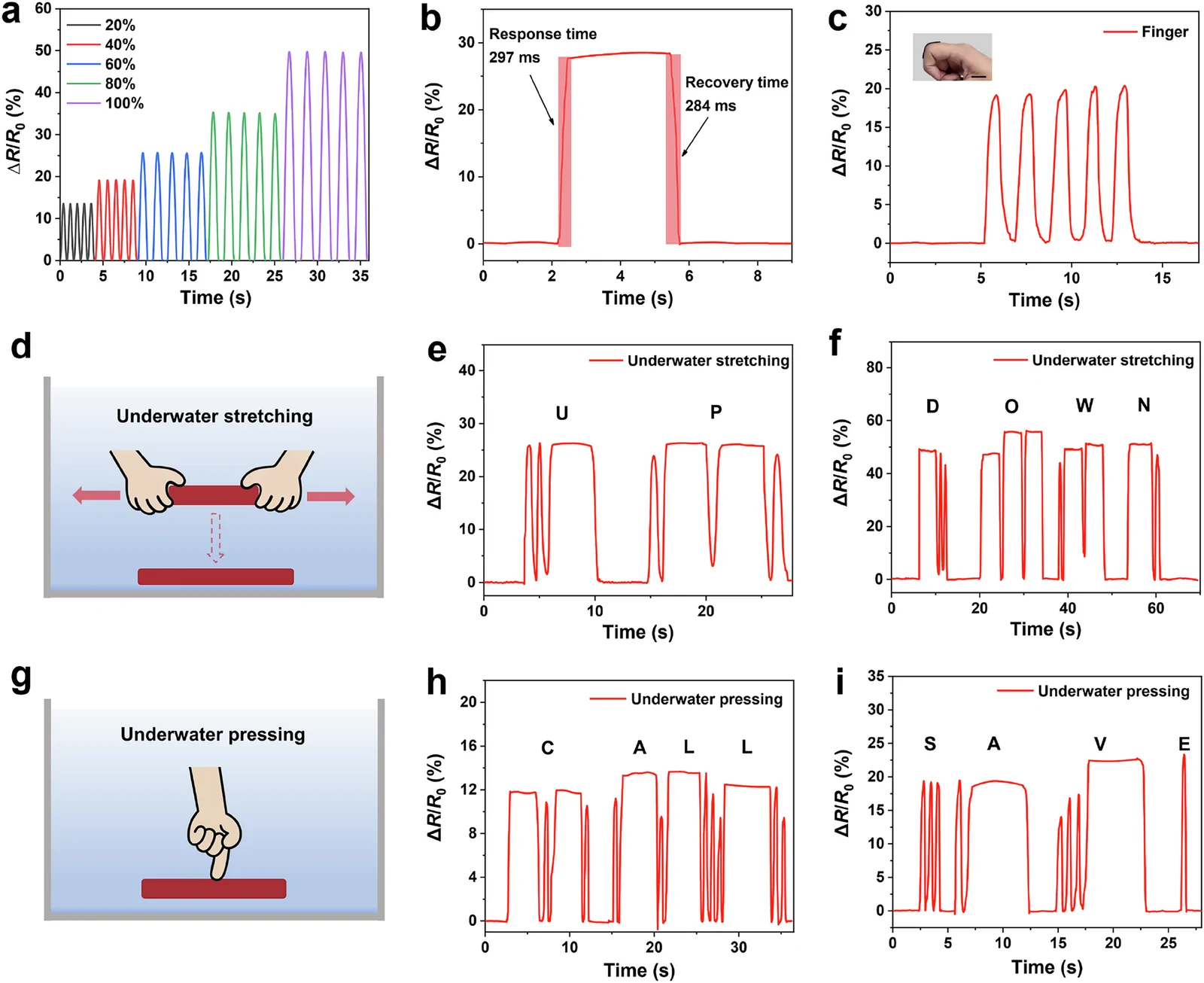
Sensing characteristics of IWH. **a** Relative resistance change of IWH under strains ranging from 20% to 100%. **b** Stretching and recovery response times. **c** Relative resistance change during finger bending. Scale bar: 2 cm. **d** Schematic illustration of underwater stretching. **e**, **f** Simulation of information transmission using Morse code via stretch-induced variable resistance signals. **g** Schematic illustration of underwater pressing. **h**, **i** Simulation of information transmission using Morse code via press-induced variable resistance signals.

In addition to enhancing the macroscopic performance of IWH, the incorporation of metal salts can also provide IWH with excellent ionic conductivity. As the concentration of Fe^3+^ increases, the ionic conductivity of IWH also rises, reaching a maximum conductivity of 9.2 × 10^−1^ S m^−1^ at room temperature (Supplementary Fig. 30). However, when the Fe^3+^ concentration is too high, an increase in crosslinking density restricts the migration ability of Fe^3+^, leading to a decrease in conductivity instead. Furthermore, cytotoxicity assessments indicate preliminary in vitro cytocompatibility of the hydrogel (Supplementary Figs. 31, 32). After immersion in simulated sweat, the leaching amount of Fe^3+^ is low, indicating that the release of Fe^3+^ is within a safe range (Supplementary Fig. 33). Subsequently, we show the application potential of IWH through various demonstrations. Firstly, IWH can serve as a sensor for strain sensing. Within the strain range of 20–100%, it can maintain a stable response in terms of resistance change (Fig. 5a). The magnitude of its resistance change increases with increasing strain (Supplementary Fig. 34). Meanwhile, IWH also has a rapid response, with the stretching and recovery response times of 297 ms and 284 ms, respectively (Fig. 5b). Benefiting from its good conductivity and response sensitivity, IWH can real-time monitor human motion. For example, when body parts such as fingers bend, IWH stretches accordingly and its resistance increases (Fig. 5c). It can also stably capture signal variations for larger deformations of body parts (Supplementary Fig. 35). When subjected to pressing, the resistance of IWH decreases, and the pressing speed and force can be identified by analyzing the frequency and amplitude of the resistance signal curve (Supplementary Fig. 36).

In addition, IWH can maintain a stable structure and function underwater owing to the excellent anti-swelling properties, making it suitable for underwater sensing scenarios. When fixed on a finger, it can sensitively detect bending movements underwater (Supplementary Fig. 37). Furthermore, effective information transmission can be achieved by combining it with Morse code. Morse code is a coding method that represents letters through a sequence of combinations of “dots (·)” and “dashes (−)”^42^. We distinguish between “·” and “−” by controlling the duration of stretching. Different combinations can transmit different letters or words underwater, enabling covert information transfer. For example, signals such as “UP” and “DOWN” are transmitted through underwater stretching (Fig. 5d–f), while signals like “CALL” and “SAVE” are conveyed through underwater pressing (Fig. 5g–i). Moreover, since there is a difference in the environmental pressure that IWH experiences in air and water, its resistance increases when it moves from water into the air. Therefore, we can also generate distress signals such as “SOS” and “HELP” by having IWH enter and leave the water surface (Supplementary Fig. 38). Thus, IWH provides a type of interaction mode for underwater communication.

### Dynamic shape memory and intelligent 3D/4D printing

**Fig. 6:**
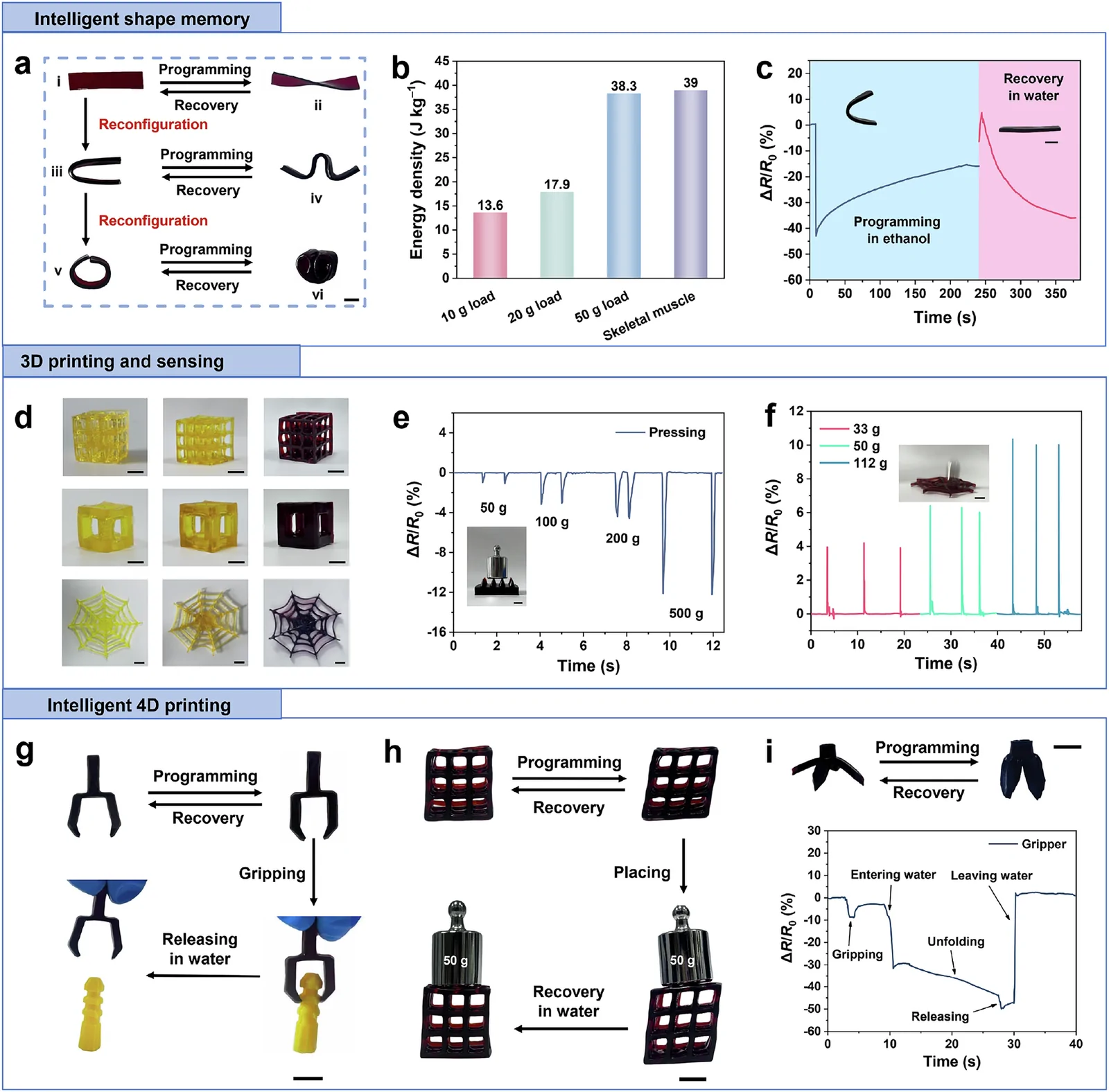
Shape memory and intelligent 3D/4D printing. **a** Reconfigurable shape memory of IWH. Scale bar: 1 cm. **b** Energy density of IWH performing work on different weights and its comparison with the energy density of human muscles. **c** Relative resistance change during the programming and recovery processes of IWH. Scale bar: 1 cm. **d** 3D-printed P(AM-*co*-AA) (left), dried P(AM-*co*-AA) (middle), and IWH (right) in various shapes. Scale bar: 2 cm. **e** Relative resistance change of 3D-printed IWH in the shape of a microneedle array when bearing different weights. Scale bar: 2 cm. **f** Relative resistance change of spider-web-shaped IWH protecting against the impact of small balls of different weights. Scale bar: 2 cm. **g** 3D-printed clip capable of grasping and releasing objects. Scale bar: 2 cm. **h** Printed hollow cube-shaped IWH that can still recover to its original shape after being programmed into an inclined state and bearing a weight of 50 g. Scale bar: 2 cm. **i** Relative resistance change of a printed claw during the process of grasping and releasing objects. Scale bar: 2 cm. The bar chart column in (**b**) represents a single measurement.

IWH also exhibits appealing solvent-induced shape programming and water-activated reversible recovery characteristics without the need for external heating. As shown in Supplementary Fig. 39, the IWH can undergo shape programming to form a temporary shape when it is in a bent state and immersed in anhydrous ethanol. When placed back in water, it can rapidly recover its original shape. The shape memory triggered by solvent stimulation is attributed to the fact that the competition between ethanol and water molecules within the polymer network weakens the water-polymer hydrogen bonds, causing water molecules to migrate out of the network. This leads to the immobilization of polymer chains. Re-absorption of water can restore the segmental mobility of polymer chains, enabling shape recovery. It is worth mentioning that the shape-memory behavior of IWH is reconfigurable because of the dynamic nature of the coordination network. The schematic of shape memory and reconfiguration process is shown in Supplementary Fig. 40. IWH can achieve shape memory through immersion in ethanol and water, and can be immersed in an FeCl_3_ solution to realize shape reconfiguration. As shown in Fig. 6a, IWH can be programmed into a temporary shape (ii) through ethanol stimulation and then recover to a permanent shape (i) in water. Subsequently, its shape can be fixed as a permanent shape (iii) by bending IWH into a U-shape and placing it in a 1 M FeCl_3_ solution for reprogramming. Then, the permanent shape (iii) can be programmed into a temporary shape (iv) through ethanol stimulation, and the temporary shape (iv) can recover to the permanent shape (iii) in water. Furthermore, when we bend IWH into a circular shape and re-place it in the FeCl_3_ solution for reprogramming, its shape can be fixed as a permanent shape (v). Then, the permanent shape (v) can be programmed into a temporary shape (vi) through ethanol stimulation, and the temporary shape (vi) can recover to the permanent shape (v) in water. Therefore, the dynamic ion-weaving network enables the reconfigurable shape memory behavior of IWH.

In addition, the water-driven shape memory process of IWH is converted into performing work on external objects (e.g., lifting weights), mimicking the actuation process of artificial muscle. The calculated energy density of the IWH increases with the increase in the load weight, similar to the working mode of muscle fibers (Fig. 6b). The maximum energy density can reach 38.3 J kg^−1^, which is close to that of human skeletal muscle, indicating its potential for water-sensitive artificial muscle applications. Moreover, we can synchronously sense the shape memory process by monitoring the electrical signals during deformation. Taking the resistance of the bent IWH (temporary shape) in air as the baseline, the resistance initially drops when it is immersed in anhydrous ethanol for shape programming (Fig. 6c and Supplementary Fig. 41). Subsequently, the resistance gradually increases because of the dehydration inside the gel. When the gel is completely dehydrated after 200 s, the resistance starts to remain stable. At the initial stage of immersing the gel in water for shape recovery, its resistance increases briefly. Then, the gel gradually recovers to its permanent shape as ethanol is replaced by water. Meanwhile, the ion migration rate increases, causing the resistance to slowly decrease and finally stabilize.

Digital light processing (DLP) 3D printing is an efficient polymer additive manufacturing technology that can rapidly construct complex structures without the need for expensive and time-consuming mold-making processes. Given the promising prospects of hydrogels in fields including flexible electronics, this technology can be extended to hydrogel systems^43–46^. With the help of 3D printing technology, we can fabricate IWH materials in various complex shapes, such as cubes, spider webs, and microneedle arrays (Fig. 6d and Supplementary Fig. 42a). The 3D-printed IWHs also exhibit excellent mechanical properties. For instance, the IWH microneedle array shows no obvious deformation even under a 1 kg load, demonstrating high modulus (Supplementary Fig. 42b). However, the P(AM-*co*-AA) hydrogel microneedle array suffers severe deformation under a 200 g load (Supplementary Fig. 42c). Combining its sensing characteristics, the printed microneedle array can be used as a pressure sensor. The resistance decreases as the load weight increases (Fig. 6e). Moreover, the spider-web-shaped IWH can retain its structural integrity after withstanding the free-fall impact of an iron ball dropped from a height of 60 cm and achieve synchronous impact signal detection, showing good intelligent protective effects (Fig. 6f).

In addition, the 3D-printed IWHs can change their shapes through external stimuli after fabrication. This processing method with a time-dependent response is known as 4D printing^47–50^. To demonstrate the 4D printing capability of IWH, we printed a flower-shaped IWH to simulate the opening and closing behavior of real flowers (Supplementary Fig. 43). The 3D-printed open flower is programmed into a closed state in anhydrous ethanol and then gradually opens into a blooming state when placed in water. Similarly, the 3D-printed clip can be programmed into a closed state to grasp objects (Fig. 6g). Subsequently, it can reopen to release the objects when placed in water. Moreover, we also printed a hollow-cube-shaped IWH, programmed it into an inclined state, and placed a heavy object on top (Fig. 6h). The inclined hollow cube can still sustain the load without deformation. Furthermore, it can recover to its original shape even with a heavy object present when placed in water. Then, we integrate the 4D printing process with flexible sensing functions to achieve self-sensing 4D-printed materials. As shown in Fig. 6i and Supplementary Fig. 44, when the 3D-printed claw with a temporarily closed state grasps an object, its resistance drops and remains stable. When the claw with the grasped object is placed in water, its resistance experiences an instantaneous and rapid decrease. Subsequently, the water-activation process causes the claw to gradually open, leading to a further slow decrease in resistance. Finally, the object is released and sinks to the bottom. When the claw is taken out of the water, its resistance increases to a stable value. Similarly, the 3D-printed IWH chair can also sense pressure after deformation (Supplementary Fig. 45). The resistance decreases when a 200 g weight is placed on the programmed chair. And when the weight is removed, the resistance recovers to its original state. Therefore, IWH can achieve intelligent 4D printing with integrated actuating and sensing capabilities. The comparison of the functional features of IWH with those of previously reported work is shown in Supplementary Table 4, which highlights the advantages of our strategy.

## Discussion

In conclusion, this work develops a family of hydrogel materials with good mechanical properties, environmental stability, and intrinsic sensing capabilities. The core of these materials lies in an ionic weaving strategy. This strategy utilizes restricted drying to afford a densely aligned polymer chain template for weaving. Subsequently, dynamic coordination bonds are formed among the pre-organized polymer chains by introducing metal ions, thereby constructing a uniform 3D topological crosslinking network. This design not only confers IWH with high strength, high modulus, and high toughness through the synchronous dissociation of coordination bonds but also grants it anti-swelling, anti-freezing, and dehydration-rehydration activation properties through topological confinement effects. Benefiting from these attributes, the IWH can achieve stable underwater sensing and solvent-stimulated shape memory functions, as well as integrated actuation and sensing capabilities. More importantly, we also realize the 3D printing of IWH with complex structures and demonstrate its intelligent 4D printing behavior that seamlessly integrates “actuation-complex structure-sensing” in the spatial and temporal dimensions, showcasing its enormous potential in constructing next-generation soft robotics and intelligent systems. This work opens up an avenue for the design of intelligent soft materials.

## Methods

### Materials

Acrylamide (AM), acrylic acid (AA), 2-hydroxy-2-methylpropiophenone (HMPP), and *N*,*N’*-methylenebisacrylamide (MBAA) were purchased from Macklin. Ferric chloride (FeCl_3_) was purchased from Sinopharm Chemical Reagent Co., Ltd. All reagents are used directly without further purification.

### Preparation of hydrogel

AM (1.6 g) and AA (0.32 g) were added to 17.71 g of deionized water. Then, MBAA (0.05 mol%) and HMPP (0.01 mol%) were introduced as the cross-linking agent and initiator, respectively. After thorough mixing, a precursor solution was obtained. Subsequently, the precursor solution was poured into a hollow glass mold. The mold was exposed to 365 nm ultraviolet light for 5 h to initiate polymerization, resulting in the formation of P(AM-*co*-AA) hydrogel. The P(AM-*co*-AA) hydrogel was then removed from the mold and placed on a smooth, flat glass plate. The sample dimensions were 5 cm × 5 cm in length and width. Its four sides were secured with clamps onto a glass slide without pre-applied tension, and it was left to dry naturally in an indoor environment (25 °C, 60% RH, natural convection) for one day. The fixed boundaries generated internal forces during drying to achieve the pre-alignment of polymer chains. The endpoint of drying was defined as the sample reaching a constant weight state, *i.e*., no significant mass change between two consecutive weighings. After that, the dried P(AM-*co*-AA) was immersed in a 0.1 M FeCl_3_ solution for 24 h. Finally, the hydrogel was soaked in deionized water for one day to remove excess Fe^3+^ ions, yielding the ionic weaving hydrogel (IWH). For the traditional metal-coordination hydrogel (MCH) as a control, the P(AM-*co*-AA) hydrogel was directly immersed in a 0.1 M FeCl_3_ solution for 24 h, followed by soaking in deionized water for 24 h. By varying the monomer ratio, metal ion concentration, monomer type, and ion type, other hydrogels can also be prepared.

### Characterization

The hydrogel samples were tested using an infrared spectrometer equipped with attenuated total reflection (ATR) infrared spectroscopy technology. The test was conducted within the infrared spectral region with a wavenumber range of 4000–600 cm^−1^. After freeze-drying the hydrogel samples, they were tested using an X-ray photoelectron spectrometer. The hydrogel samples were cut into 5 mm × 5 mm squares. After being frozen with liquid nitrogen, they were vacuum-freeze-dried for 24 h using a freeze-dryer to remove moisture. Then, the samples were subjected to gold-sputtering treatment. The microscopic morphology of the samples was observed using a thermal field-emission scanning electron microscope (SEM) at an accelerating voltage of 10 kV. Small-angle X-ray scattering (SAXS) and wide-angle X-ray scattering (WAXS) tests were carried out using the Ganesha system. The thermal properties were tested using a differential scanning calorimeter (DSC). The test was performed under a nitrogen atmosphere, with the temperature raised from −80 °C to 100 °C at a heating rate of 5 °C min^−1^. The samples were cut into circular shapes with a diameter of 25 mm and tested using a rotational rheometer (MCR-302, Anton Paar). The tests were conducted at room temperature (25 °C). With a fixed shear strain of 1%, frequency-sweeping tests were carried out on the samples within the angular frequency range of 0.1–100 rad s^−1^. Additionally, strain-sweeping tests were performed on the samples within the strain range of 1–100%.

### Mechanical properties

The mechanical tests of the hydrogels were conducted on a universal testing machine (Xiamen Meters Instrument) equipped with a 100 N load cell. The stretching speed was fixed at 100 mm min^−1^. The Young’s modulus was calculated from the initial slope of the elastic deformation stage of the stress-strain curve, while the toughness was obtained through the integral of the area under the stress-strain curve. The fracture energy was calculated via the single-edge crack test. The damping capacity was defined as the ratio of the dissipated energy (the area enclosed by the loading and unloading regions) to the loading energy (the area under the loading curve). The stress-strain curves of the samples were tested at strain rates of 0.1, 0.2, 0.3, 0.4, and 0.5 s^−1^, respectively, to obtain the yield stresses, which were then fitted using the Eyring model:

1 $${\sigma }_{y}\approx \frac{2{kT}}{{V}_{a}}{{{\mathrm{ln}}}}\dot{{{{\rm{\varepsilon }}}}}+\frac{2{E}_{y}}{{V}_{a}}$$

where $\dot{{{{\rm{\varepsilon }}}}}$, ${\sigma }_{y}$, ${V}_{a}$ and ${E}_{y}$ represented the deformation rate, yield stress, activation volume, and activation energy, respectively.

Circular samples with a diameter of 100 mm were fixed onto a puncture testing device, and a steel needle was used to perform puncture tests at a speed of 50 mm min^−1^. The puncture energy was calculated through the integral area under the force-displacement curve. A steel ball with a diameter of 20 mm and a weight of 33 g was dropped from a height of 100 cm to generate an impact. A tube with a diameter of 60 mm was used to prevent the steel ball from flying out. A planar pressure sensor was employed to record the impulse changes during the impact of the falling steel ball. 1 mm-thick IWH, MCH, P(AM-*co*-AA), and PDMS were placed on the sensor to compare their impact resistance.

### Swelling test

The samples were cut into square specimens of 1 cm × 1 cm with thicknesses of 0.5 mm, 1.0 mm, and 1.5 mm. The specimens with different thicknesses were immersed in deionized water at room temperature, and the sample weights were recorded periodically. All tests were performed in triplicate for each thickness, and the data are presented as mean ± standard deviation (SD) to ensure the reliability and reproducibility of the results. The swelling ratio of the specimens was calculated using the following equation:

2 $$W=\frac{{m}_{t}-{m}_{0}}{{m}_{0}}\times 100\%$$

where W is the swelling ratio, ${m}_{0}$ is the initial weight of the sample, and ${m}_{t}$ is the real-time weight of the sample after immersion.

### Electrical properties

The hydrogel was cut into circular shapes with a diameter of 16 mm and sandwiched between two stainless-steel electrodes. An electrochemical workstation (CHI 660e) was used to characterize the electrochemical impedance spectroscopy (EIS) of the hydrogel within a frequency range of 1 Hz to 1 MHz. Subsequently, the resistance (R) was determined from the obtained Nyquist plot. The ionic conductivity (*σ*) of the gel sample was then calculated using the formula:

3 $$\sigma=\frac{L}{R\times S}$$

where L and S represented the length and cross-sectional area of the sample, respectively.

### Sensing tests

A resistive-type sensor was assembled using IWH as the conductive material and silver wires as the electrodes. The IWH was fixed on body parts such as fingers and wrists. Meanwhile, different actions were performed, including bending, stretching, and pressing. The resistance changes were measured using a source-meter (Keithley 2601b). The formula for calculating the relative resistance change was as follows:

4 $$\frac{\Delta R}{{R}_{0}}=\frac{R-{R}_{0}}{R}$$

where ${R}_{0}$ represented the initial resistance, and R represented the real-time resistance during measurement. The gauge factor (GF) of the sensor was defined as:

5 $${GF}=\frac{\Delta R/{R}_{0}}{\varepsilon }$$

where $\Delta R/{R}_{0}$ represented the relative change in resistance, and ε represented the corresponding strain value during the experiment.

### Energy density test and calculation

The hydrogel was stretched to a pre-strain (100%) and then placed in anhydrous ethanol for shape programming to fix its shape. After that, the hydrogel was loaded with weights of different masses and placed in deionized water for shape recovery. The recovery performance was calculated using the formula:

6 $$W=\frac{{m}_{1}}{{m}_{2}}{gd}$$

where ${m}_{1}$ was the loaded mass, ${m}_{2}$ was the mass of the hydrogel, d was the displacement, and g was the acceleration due to gravity.

### 3D/4D printing

The water content of the precursor solution was reduced to 60%. Meanwhile, the initiator was replaced with LAP (0.05 mol%), and 0.3 vol% of co-initiator was added to prepare 3D printing ink. The process parameters for 3D printing were set as a layer thickness of 100 μm and an exposure time of 20 s per layer. Subsequently, the model was imported into a light-curing 3D printer (Whale 2 Nova) for printing. The shape of the printed sample was then altered through solvent stimulation.

### Determination of sensing signals during actuation

A source-meter was used to record the resistance changes of the sample during the actuation process. The formula for calculating the relative resistance change was as follows:

7 $$\frac{\Delta R}{{R}_{0}}=\frac{R-{R}_{0}}{R}$$

where ${R}_{0}$ represented the initial resistance and R represented the real-time resistance during measurement.

### Cytotoxicity testing

The cytotoxicity of mouse corneal epithelial cells (purchased from Procell, catalog number CP-M120) co-cultured with different amounts of IWH was tested using the MTT assay. First, the IWH was sterilized under ultraviolet light for 24 h. Then, the sterilized IWH was immersed in a special culture medium for HCE-T at 37 °C for 24 h to obtain the leachate. The corneal epithelial cells were seeded into a 96-well plate at a density of 4 × 10^3^ cells per well and cultured in an environment of 37 °C with 5% CO_2_ for 24 h. Subsequently, the leachate was added, and the cells were cultured for another 48 h. After that, 20 μL of MTT solution (prepared as 5 mg mL^−1^ with PBS buffer) was added to each well, and the cells were cultured for 4 h. The absorbance was measured at 450 nm using a microplate reader.

### Experiments on human subjects

All volunteers involved in wearable sensors took part following informed consent after being fully informed of the experimental procedures and potential risks. Ethical approval was not required for this work.

## Supplementary information


Supplementary Information
Transparent Peer Review file


## Source data


Source data


## Data Availability

The data that support the findings of this study are available from the corresponding authors on request. The data supporting the findings of this study are available within the article and its Supplementary Information/Source Data. Source data are provided with this paper.
