# Supplementary material for: Ionic weaving network enables mechanically robust smart hydrogels with anti-swelling and anti-freezing capabilities
Source: Nat Commun. 2026 Jul 29;17:9191. doi: 10.1038/s41467-026-76165-4 (PMC13522580; doi:10.1038/s41467-026-76165-4)
Supplement: Supplementary file 1 [file 41467_2026_76165_MOESM1_ESM.pdf]

Supplementary Information for

Ionic weaving network enables mechanically robust smart hydrogels with anti-swelling and anti-freezing capabilities

Mengyu Gan,[1] *Chenxi Yang,*[1] *Xiaozheng Su,*[1] *Jiahao Kang,*[1] *Ye Lin,*[1] *Jiale Hong,*[1] *Piaopiao Zhou,*[2,3,*] *Xiaolin Lyu,*[1,*] *Zhigang Zou*[1,4]

[1] State Key Laboratory of Green and Efficient Development of Phosphorus Resources, College of Materials Science and Engineering, Fuzhou University, Fuzhou, Fujian, 350108, China

[2] Department of Lung Transplantation, Fujian Medical University Union Hospital, Fuzhou, Fujian, 350001, China

[3] Key Laboratory of Cardio-Thoracic Surgery, Fujian Medical University, Fuzhou, Fujian, 350004, China

[4] Eco-materials and Renewable Energy Research Center, College of Engineering and Applied Sciences, Nanjing University, Nanjing 210093, China

Table of Contents

Supplementary Discussion ……………………………… S3

Supplementary Figures 1-45 ……………………………… S4

Supplementary Tables 1-5 ……………………………… S26

Supplementary References ……………………………… S31

Supplementary discussion

The sensing principle of the actuation process through electrical signals is as follows. The actuation-sensing integration of IWH arises, on one hand, from the intrinsic correlation between its deformation and electrical signals, and on the other hand, from the dynamic changes of the ion coordination network under solvent action. When IWH undergoes deformation (*e.g.*, stretching, bending, and twisting), its resistance changes according to Pouillet's law. For example, the resistance increases upon stretching and decreases under compression. When IWH is immersed in ethanol, the solvent pressure first causes its resistance to drop. Subsequently, dehydration resulting from solvent exchange hinders chain mobility, thereby fixing the temporary shape, and gradually increases the resistance by suppressing ion migration. Once the solvent exchange is complete, the resistance reaches a stable value. After re-immersing IWH in water, the rehydration caused by solvent exchange promotes chain mobility, restoring the original shape. Meanwhile, the water pressure and the recovery of ion migration capability lead to a decrease in resistance. When the rehydration is complete, the resistance stabilizes again. The changes in electrical signals are closely related to macroscopic deformation, solvent diffusion, chain mobility, ion migration capability, *etc.*, enabling IWH to continuously sense the shape change process, thus achieving actuation-sensing integration.

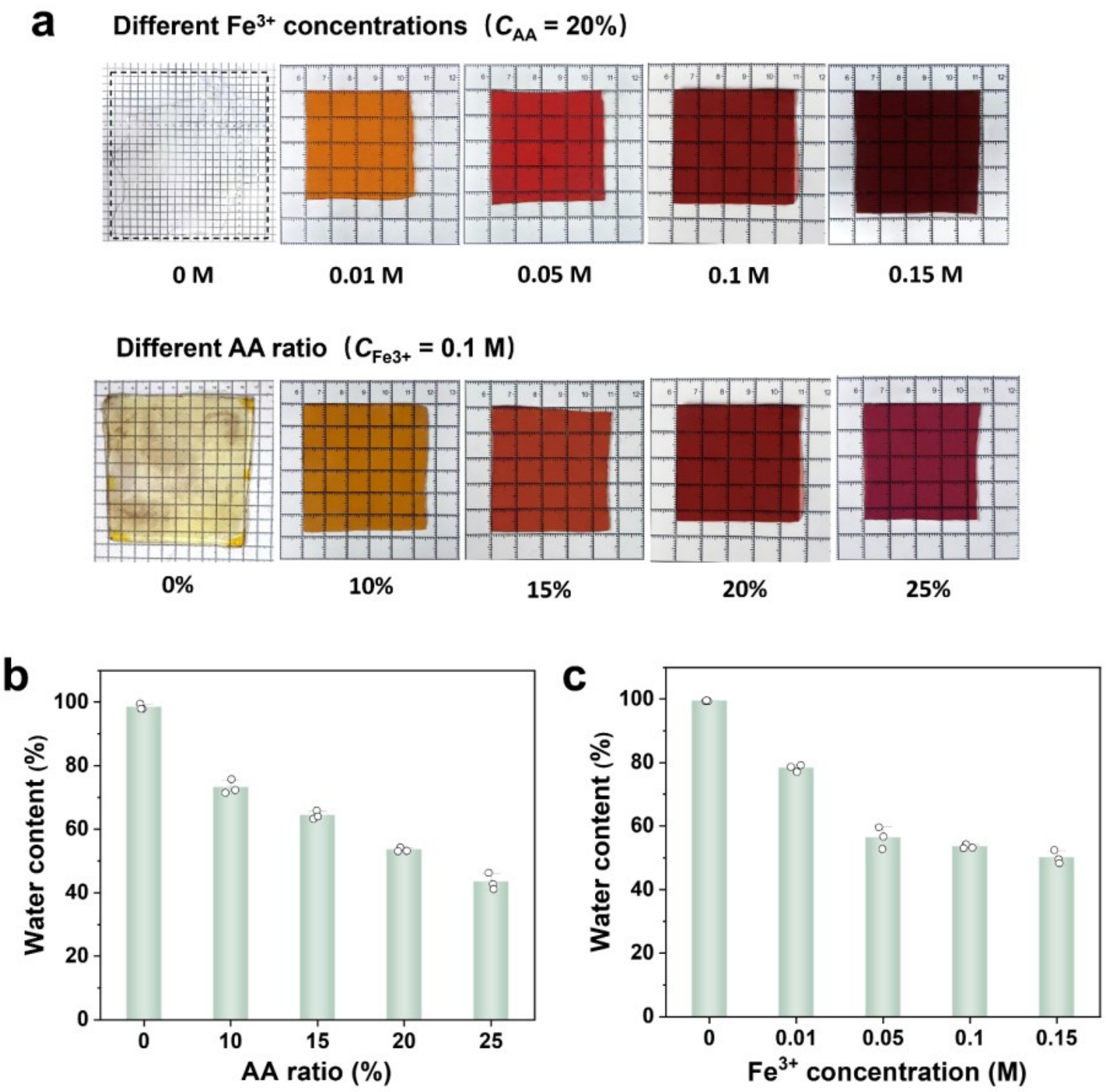

Supplementary Figure 1. Photographs and water contents of different hydrogels. a) Photographs of hydrogel samples with different Fe^{3+} concentrations and AA contents. The side length of the small square is 1 cm. b-c) Water contents of hydrogels with different AA ratios (b) and Fe^{3+} concentrations (c). Both IWH-0%-0.1 and IWH-20%-0 samples exhibit severe swelling after immersion owing to the inability to form a stable coordination network. Data in (b and c) are reported as their means ± SDs from $n = 3$ independent samples.

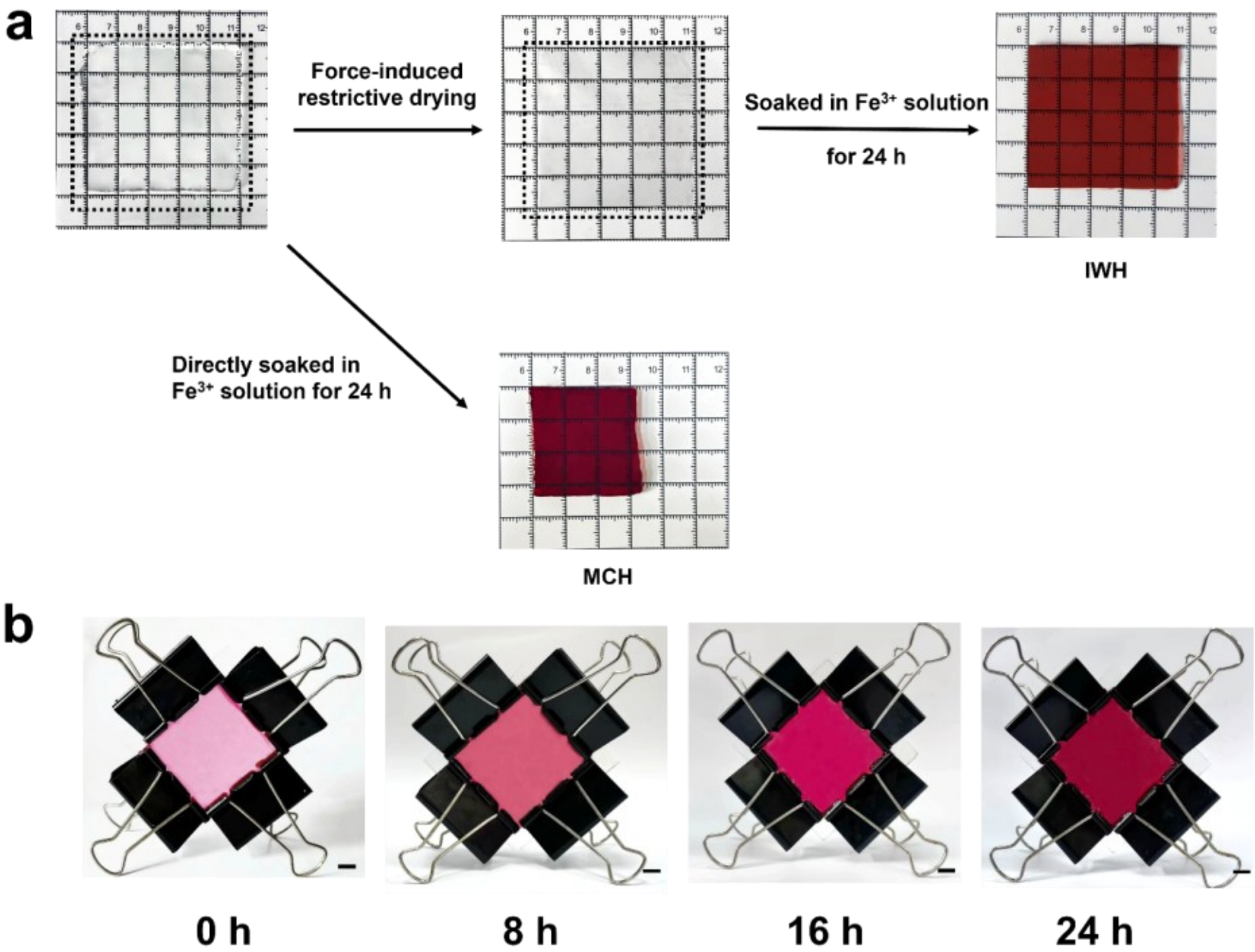

Supplementary Figure 2. Photographs of the preparation and drying processes of hydrogels. a) Photographs of the preparation steps of IWH and MCH. The side length of the small square is 1 cm. b) Photographs showing the morphological evolution of the P(AM-*co*-AA) hydrogel during restricted drying at 0, 8, 16, and 24 h. Scale bar: 1 cm. (The P(AM-*co*-AA) hydrogel was dyed red for clarity of observation).

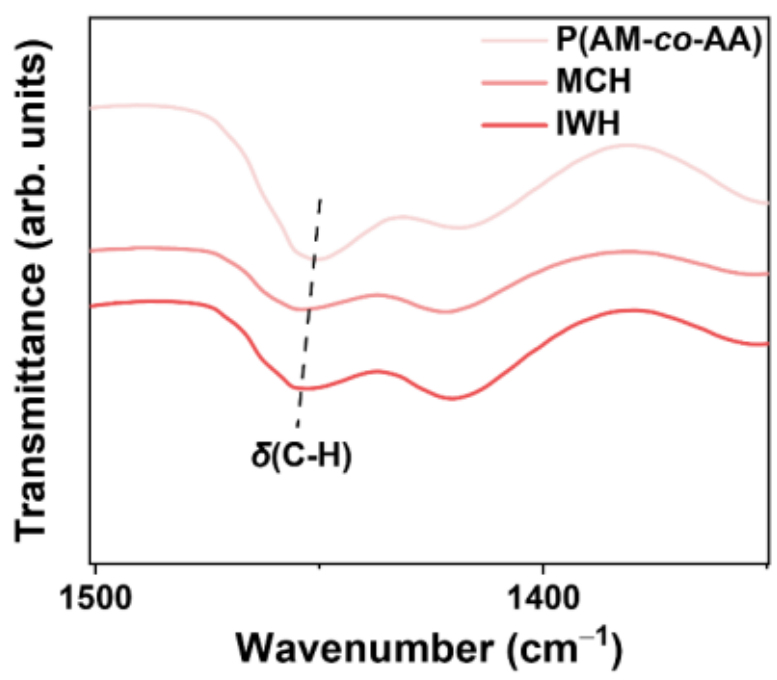

Supplementary Figure 3. Infrared spectra of δ(C-H) for IWH, MCH, and P(AM-*co*-AA). δ(C-H) exhibits blueshift after metal coordination.

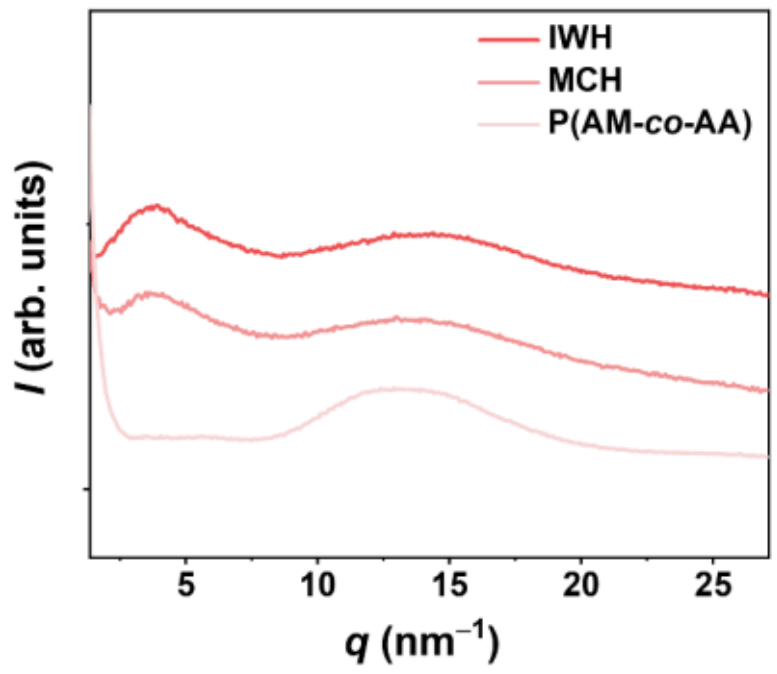

Supplementary Figure 4. 1D WAXS curves of IWH, MCH, and P(AM-*co*-AA). A new peak near 3.7 nm^{-1} occurs after metal coordination.

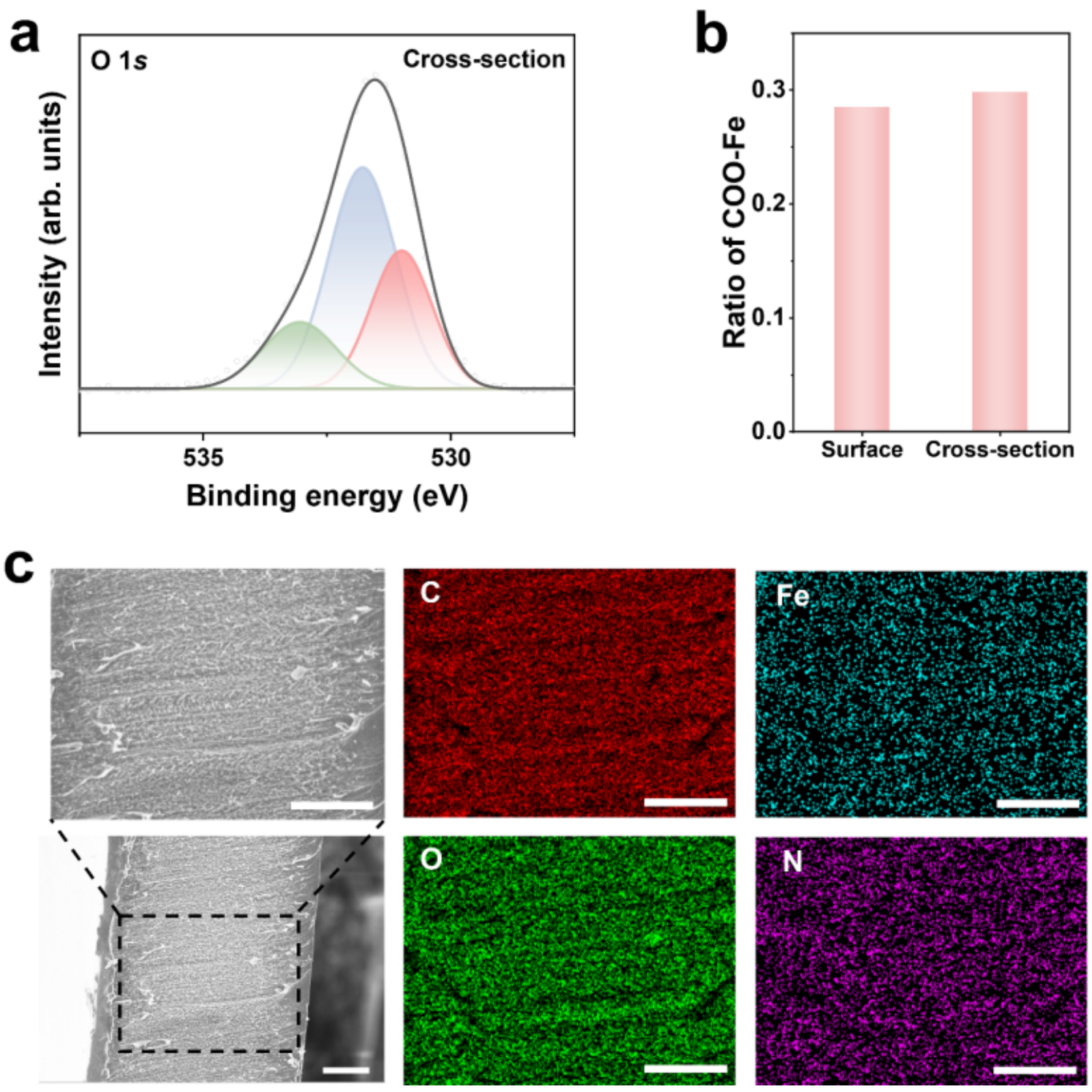

Supplementary Figure 5. Element distribution of the cross-section of IWH. a) O 1*s* XPS spectra of the cross-section of IWH. Green: C-O, blue: C=O, red: COO^{-}–Fe^{3+}. b) Peak area ratios of Fe^{3+}-COO^{-} in the surface and cross-section of IWH. c) Elemental mapping of the cross-section of IWH. Scale bar: 50 μm. The bar chart column in (b) represents a single measurement.

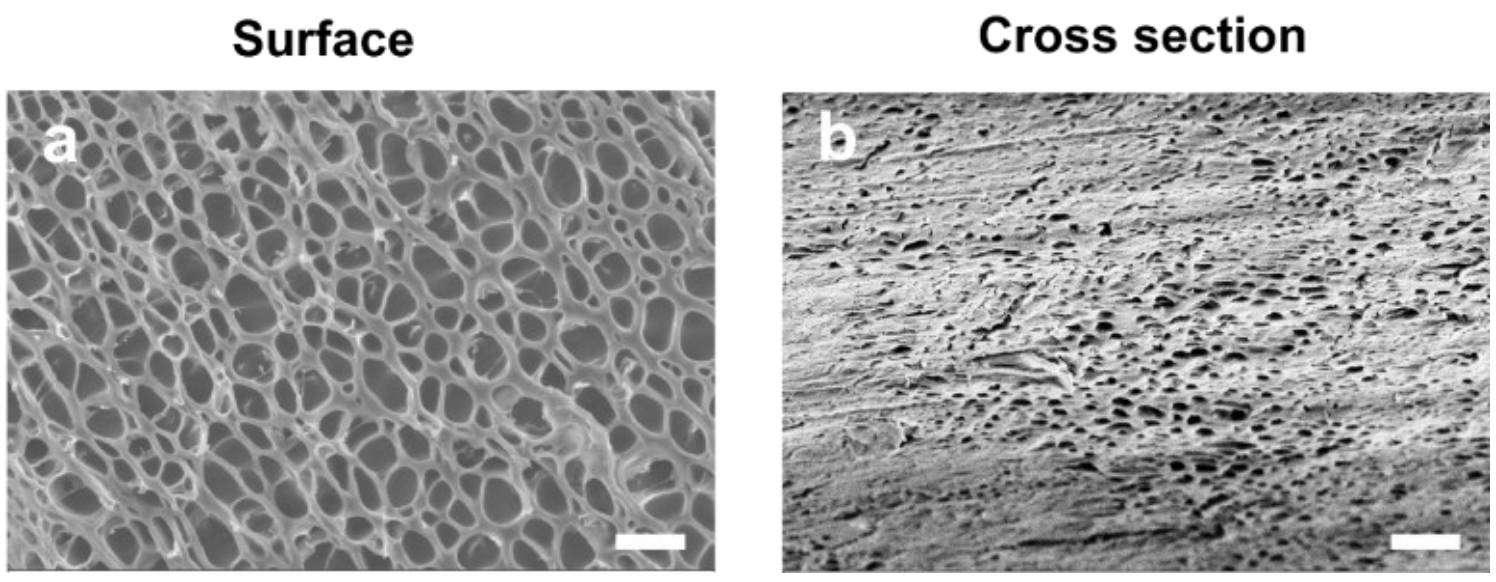

Supplementary Figure 6. SEM images of the P(AM-*co*-AA) hydrogel. a) Surface. Scale bar: 10 µm. b) Cross-section. Scale bar: 20 µm.

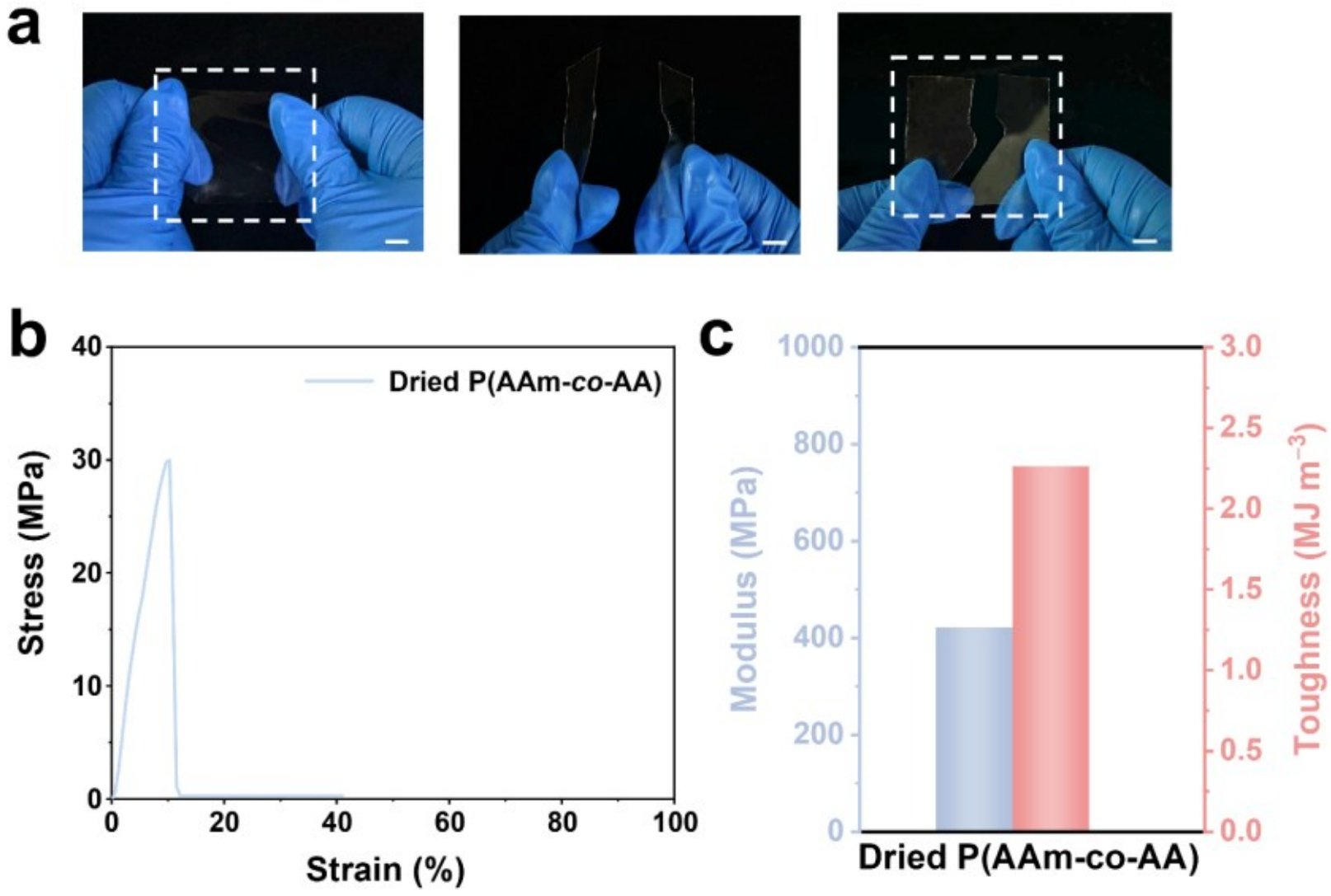

Supplementary Figure 7. Mechanical properties of dried P(AM-*co*-AA). a) Brittle fracture of the dried sample during bending. Scale bar: 1 cm. b) Stress-strain curves of the dried sample. c) Modulus and toughness of the dried sample. The bar chart column in (c) represents a single measurement.

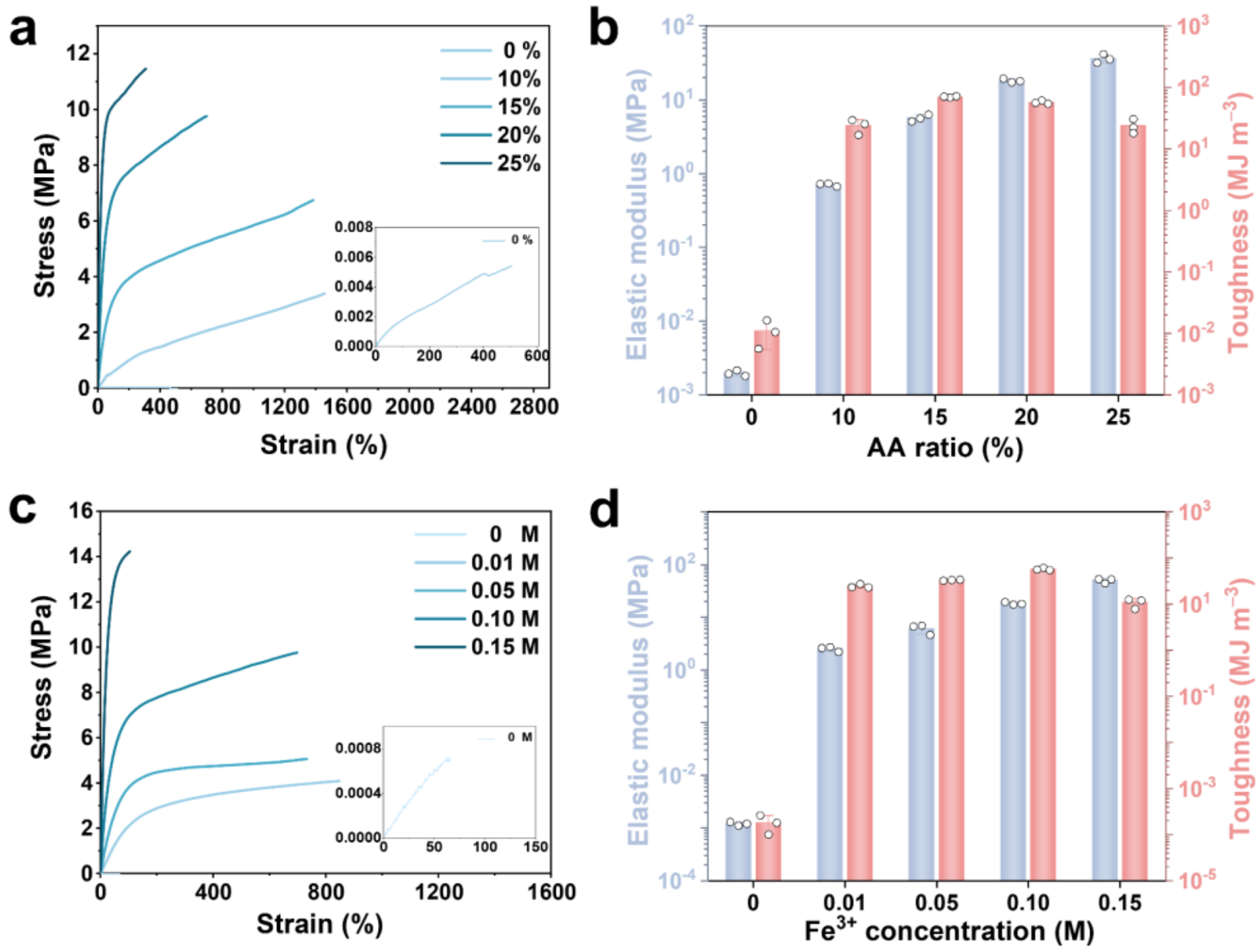

Supplementary Figure 8. Mechanical properties of hydrogels with different AA ratios and Fe^{3+} concentrations. a-b) Tensile stress-strain curves of hydrogels with different AA ratios (a) and the corresponding modulus and toughness (b). c-d) Tensile stress-strain curves of hydrogels with different Fe^{3+} concentrations (c) and the corresponding modulus and toughness (d). The mechanical properties of IWH-0%-0.10 and IWH-20%-0 are very poor owing to the inability to form a stable coordination network. Data in (b and d) are reported as their means ± SDs from n = 3 independent samples.

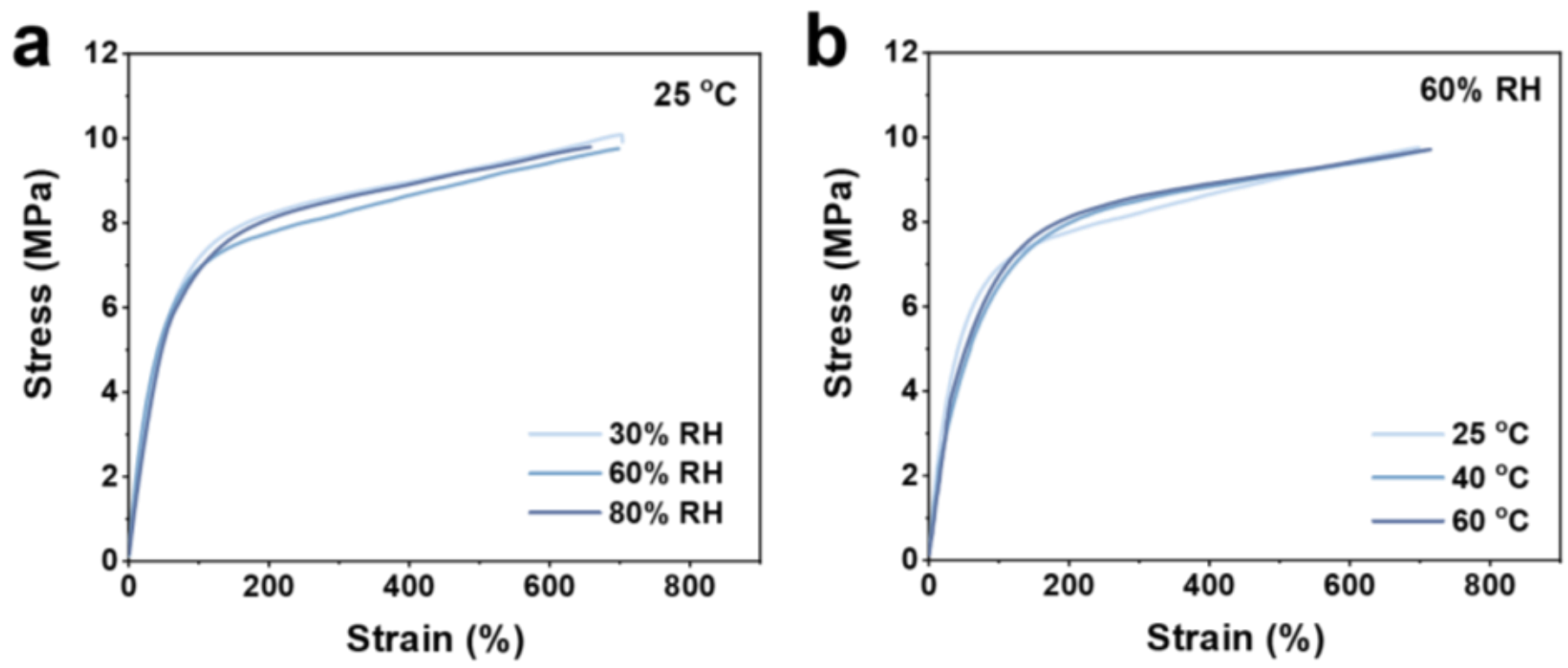

Supplementary Figure 9. Effect of restricted drying under different environmental conditions on the mechanical properties of IWHs. Stress-strain curves of IWHs prepared under different humidities (a) and different temperatures (b).

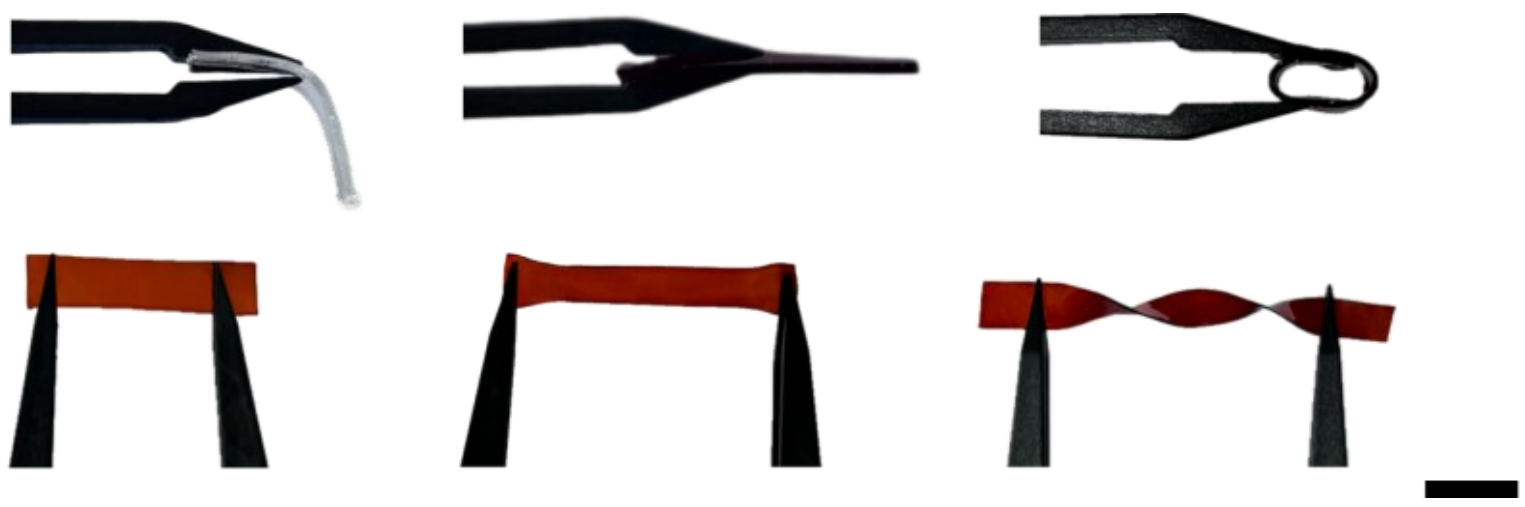

Supplementary Figure 10. Photoshops of IWH being stretched and kinked. The IWH exhibits good deformability. Scale bar: 2 cm.

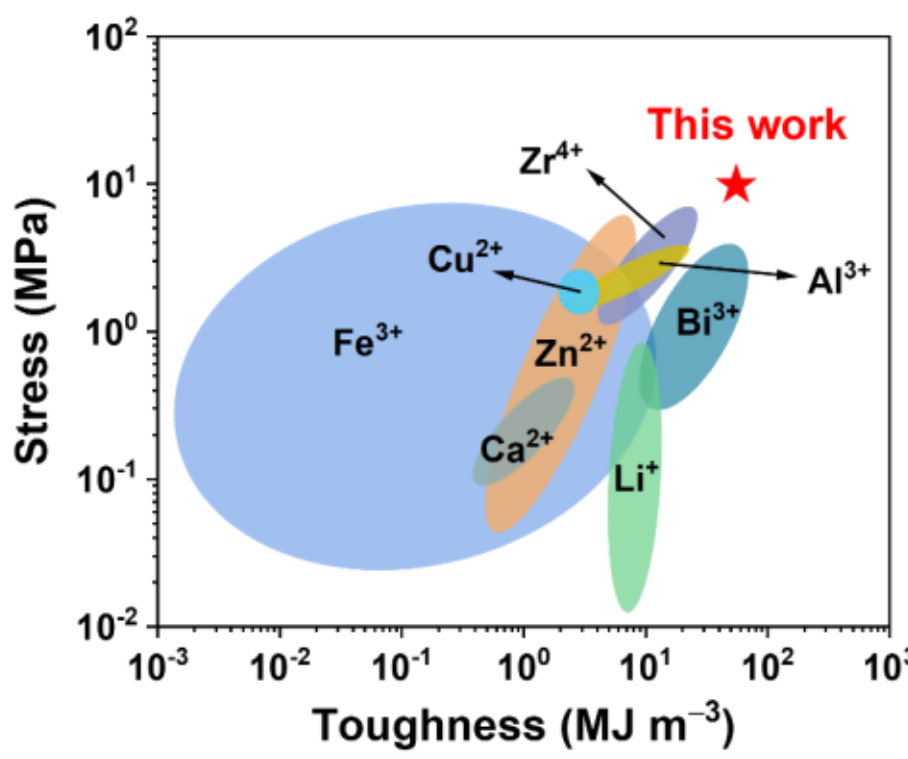

Supplementary Figure 11. Comparison of IWH with different metal coordination hydrogels in terms of toughness and stress. The IWH exhibits good mechanical properties.

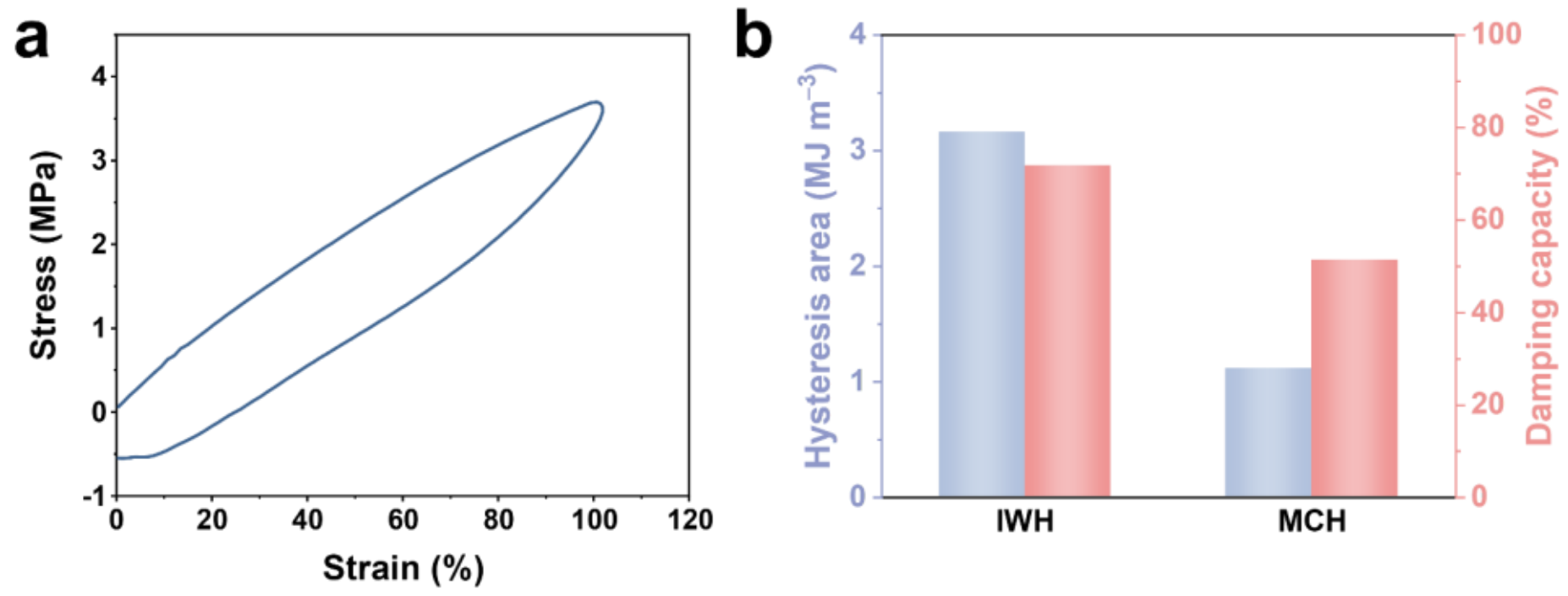

Supplementary Figure 12. Mechanical properties of MCH. a) Cyclic stress-strain curve of MCH at a strain of 100%. b) Dissipated energies and damping capacities of IWH and MCH. The bar chart column in (b) represents a single measurement.

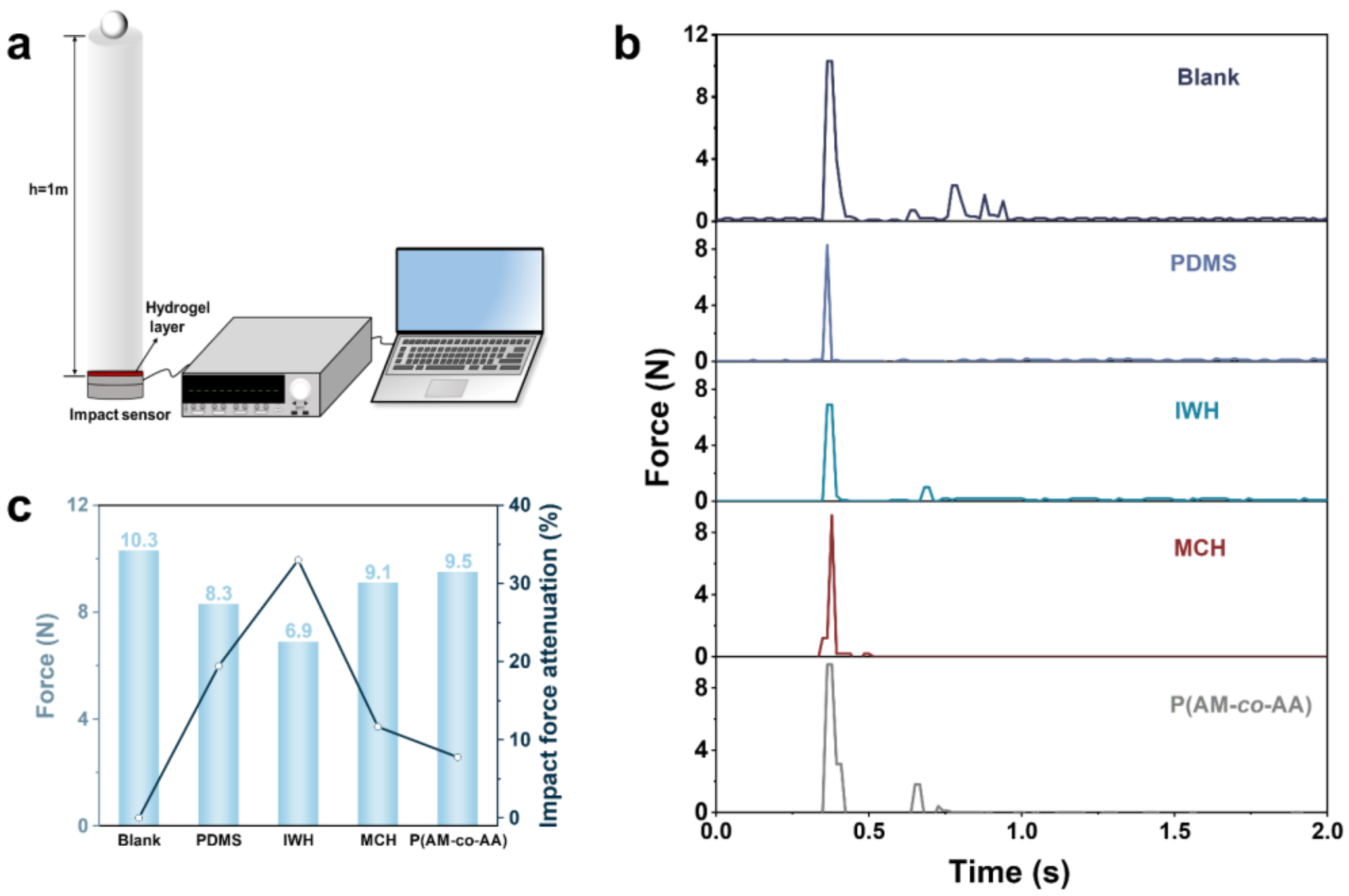

Supplementary Figure 13. Demonstration of impact resistance. a) Schematic diagram of the impact testing apparatus. b) Force-time curves recorded by sensors under cushioning pads made of IWH and other materials during impact tests. c) Shock-absorbing effects of IWH and other materials against impact forces. The bar chart column in (c) represents a single measurement.

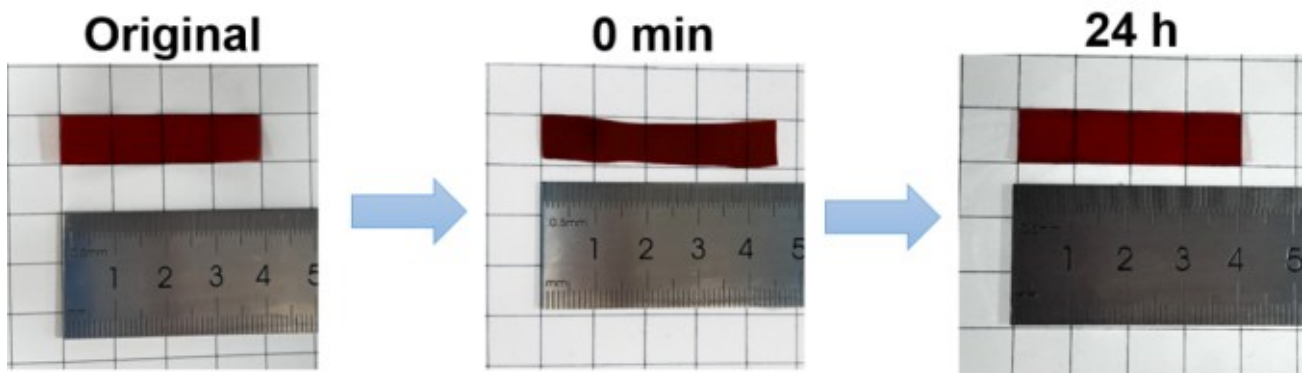

Supplementary Figure 14. Self-recovery ability of IWH. Photographs of IWH recovering to its original length within 24 hours after stretching. The side length of the small square is 1 cm.

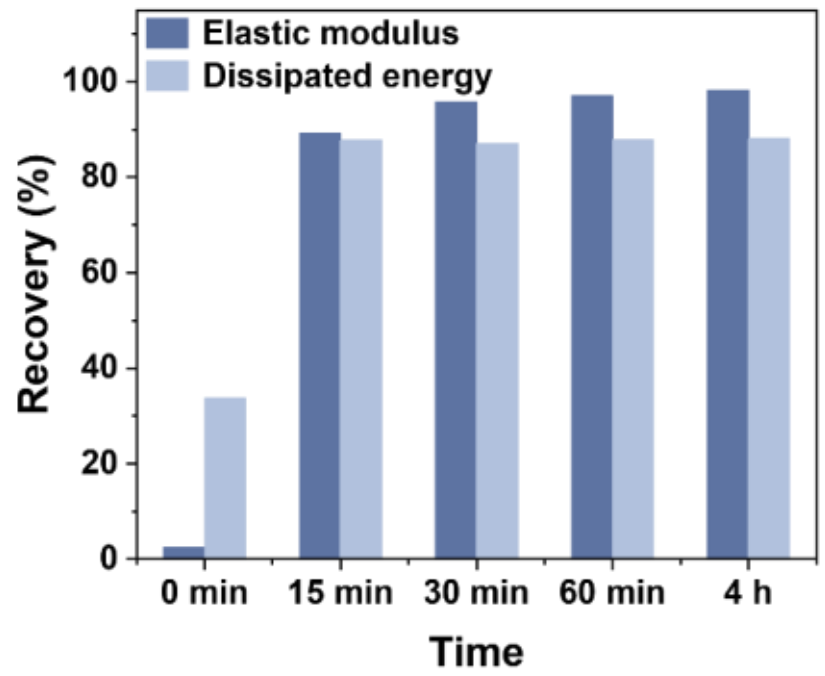

Supplementary Figure 15. Mechanical property recovery of IWH. The recovery ratios of modulus and dissipated energy for IWH after different waiting times. The bar chart column represents a single measurement.

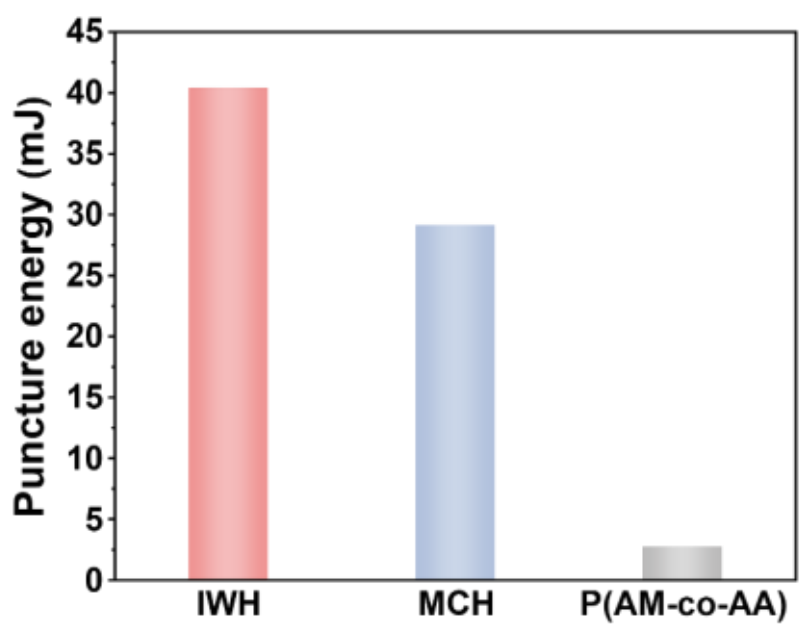

Supplementary Figure 16. Puncture energies of IWH, MCH, and P(AM-*co*-AA). The IWH exhibits the highest puncture energy. The bar chart column represents a single measurement.

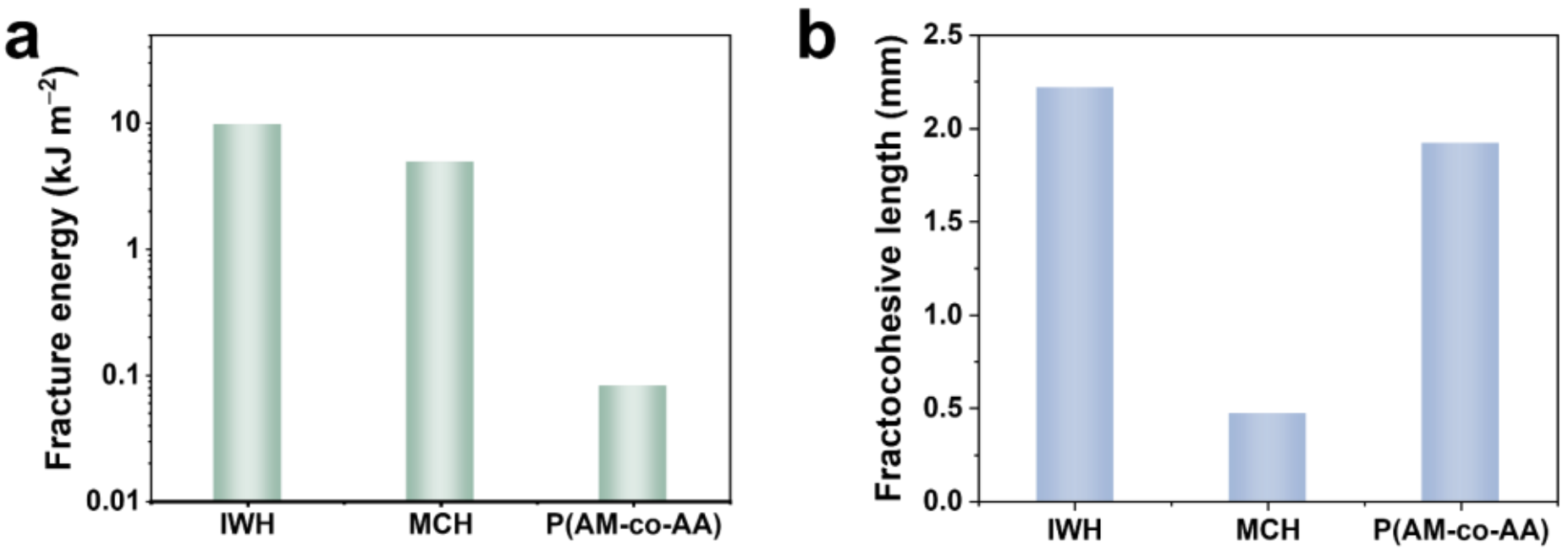

Supplementary Figure 17. Crack resistance of different hydrogels. a) Fracture energies of IWH, MCH, and P(AM-*co*-AA). b) Fractocohesive lengths of IWH, MCH, and P(AM-*co*-AA). The bar chart column in (a and b) represents a single measurement.

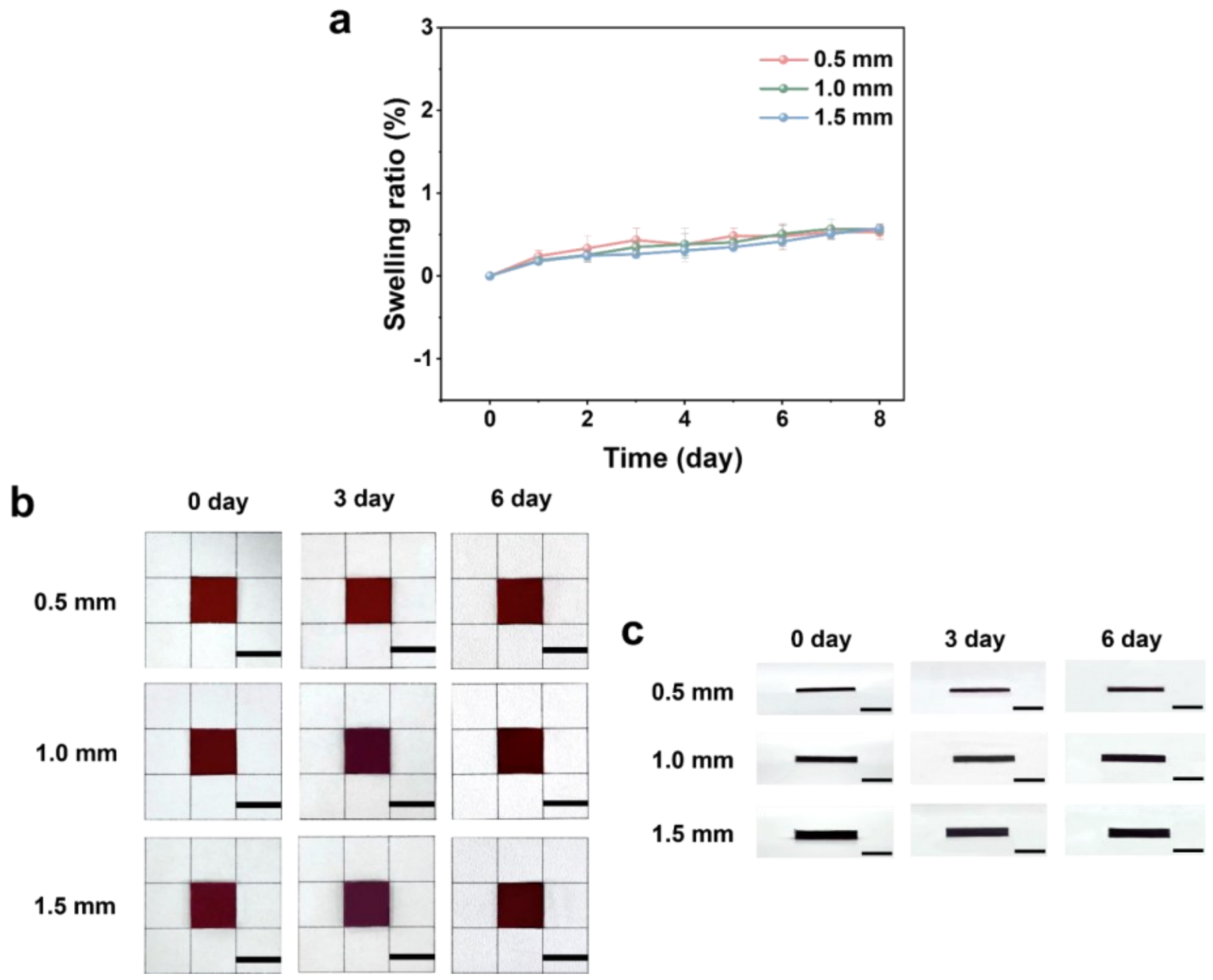

Supplementary Figure 18. Anti-swelling property of IWH. a) Swelling curves of IWHs with different thicknesses over 0–8 days. Scale bar: 1 cm. b-c) Photographs showing the changes in area (b) and thickness (c) of IWHs with different thicknesses at 0, 3, and 6 days. Scale bar: 5 mm. Data in (a) are reported as their means ± SDs from n = 3 independent samples.

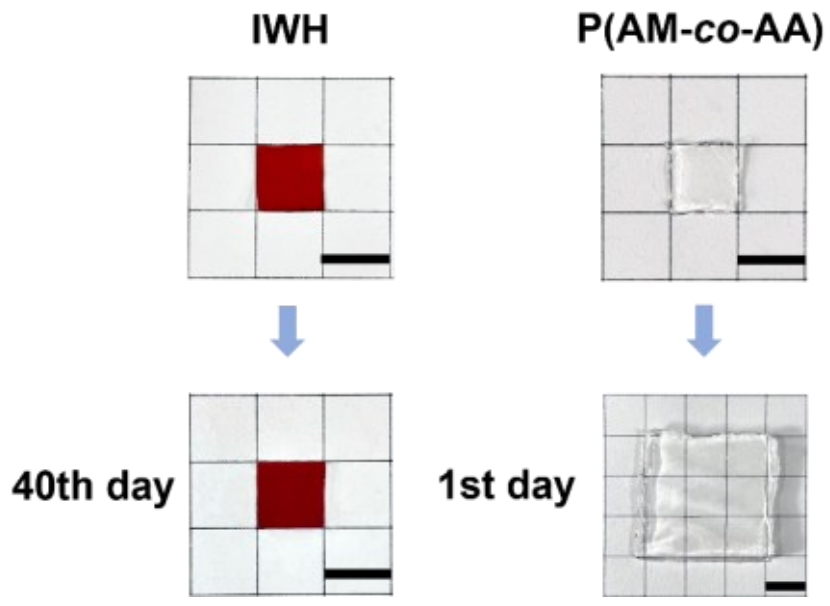

Supplementary Figure 19. Dimensional changes of IWH and P(AM-*co*-AA) after immersion in water. The IWH remains unchanged while the P(AM-*co*-AA) hydrogel swells severely. Scale bar: 1 cm.

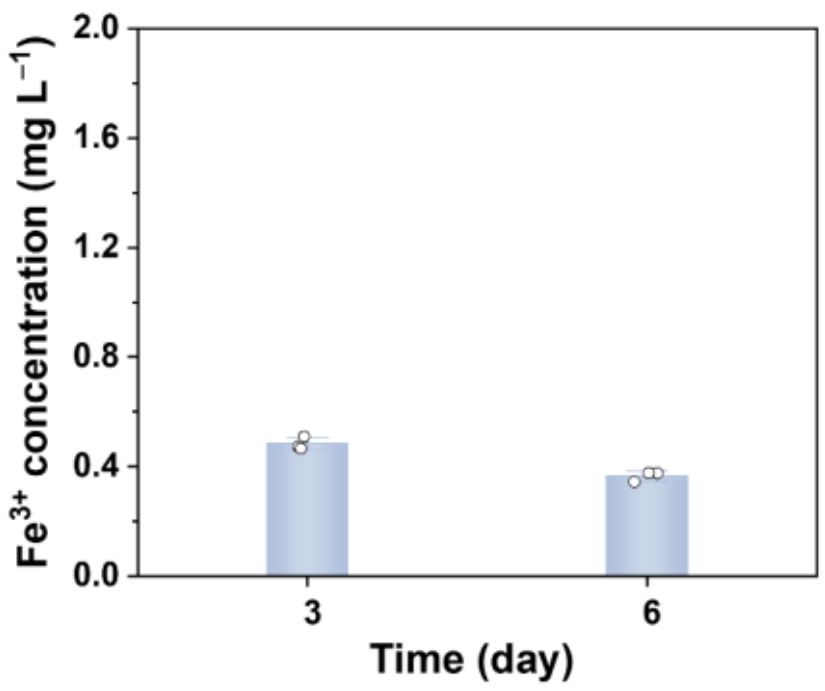

Supplementary Figure 20. Fe^{3+} concentration of the solution after immersing IWH in deionized water for 3 and 6 days. The volume of deionized water used during the swelling test is 100 mL, and the size of IWH is 1 cm × 1 cm × 1 mm. It is estimated that the leaching amount of Fe^{3+} is less than 3%. Data are reported as their means ± SDs from n = 3 independent samples.

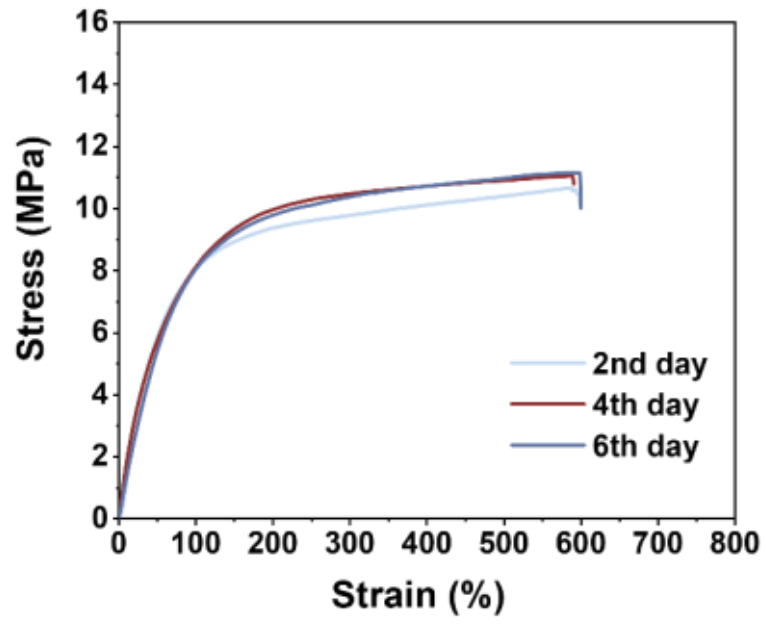

Supplementary Figure 21. Tensile stress-strain curves of IWHs after immersion in water for 2-6 days. The IWH retains good mechanical properties underwater.

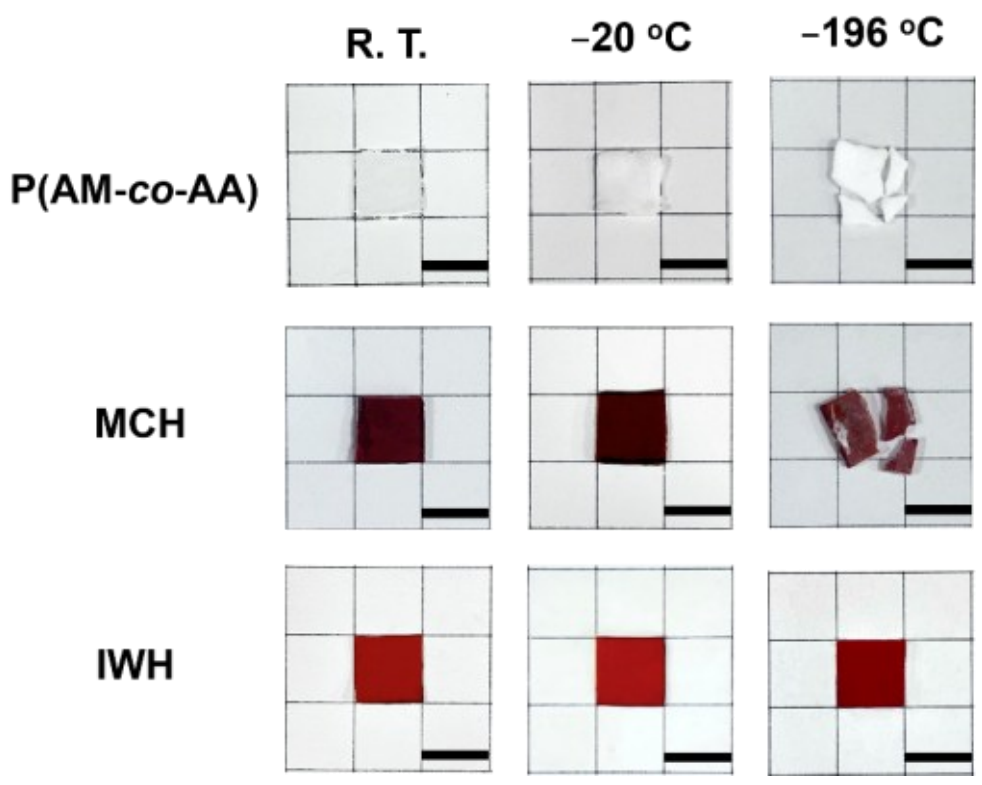

Supplementary Figure 22. Anti-freezing property of different hydrogels. Photographs of IWH, MCH, and P(AM-*co*-AA) at room temperature, –20 °C, and –196 °C. Scale bar: 1 cm.

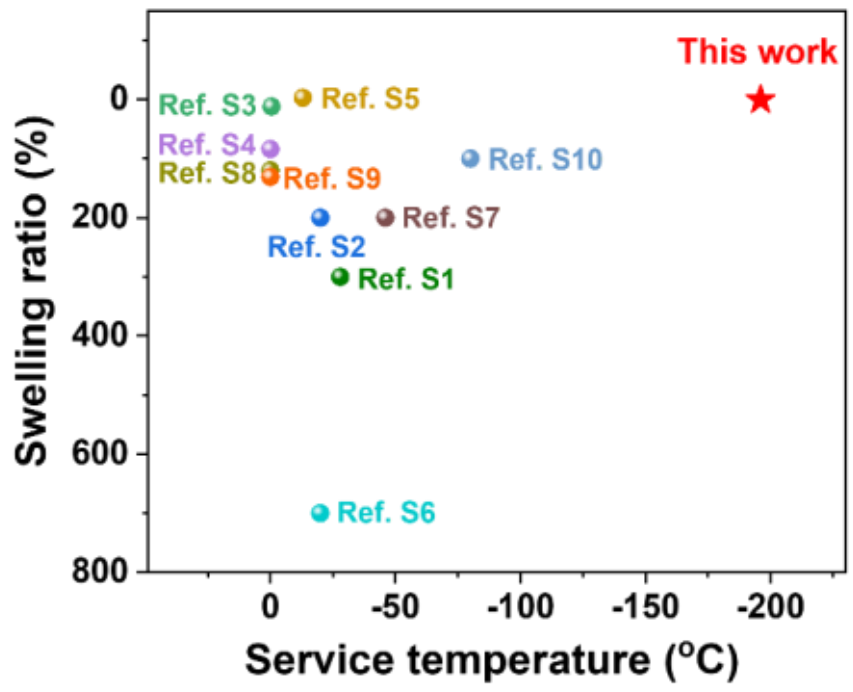

Supplementary Figure 23. Comparison of anti-swelling and anti-freezing properties between IWH and recently reported hydrogels. The IWH simultaneously possesses anti-freezing and anti-swelling capabilities.

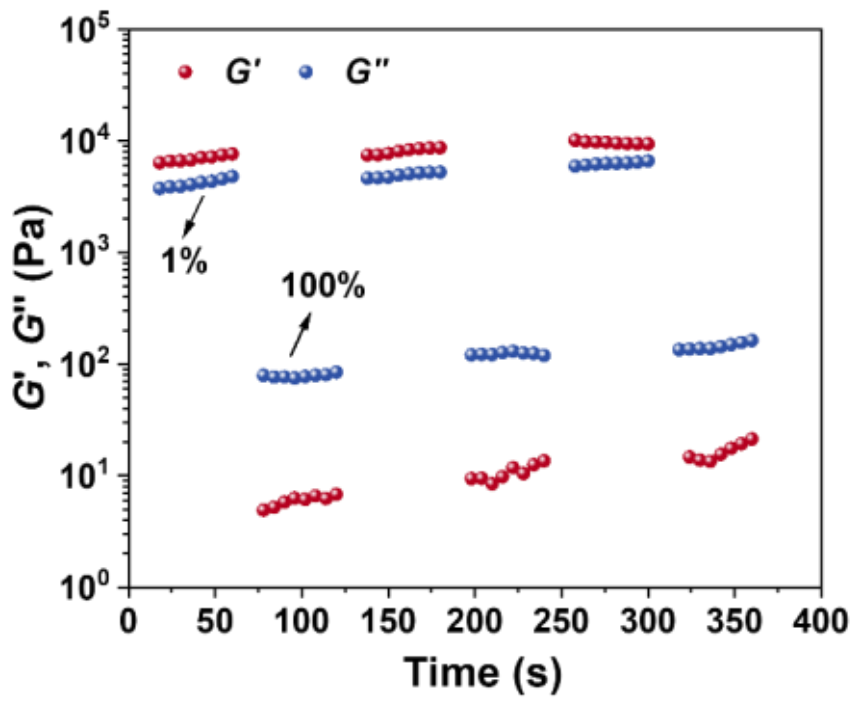

Supplementary Figure 24. Step strain rheological test of IWH with alternating shear strains of 1% and 100%. The IWH possesses a good dynamic nature.

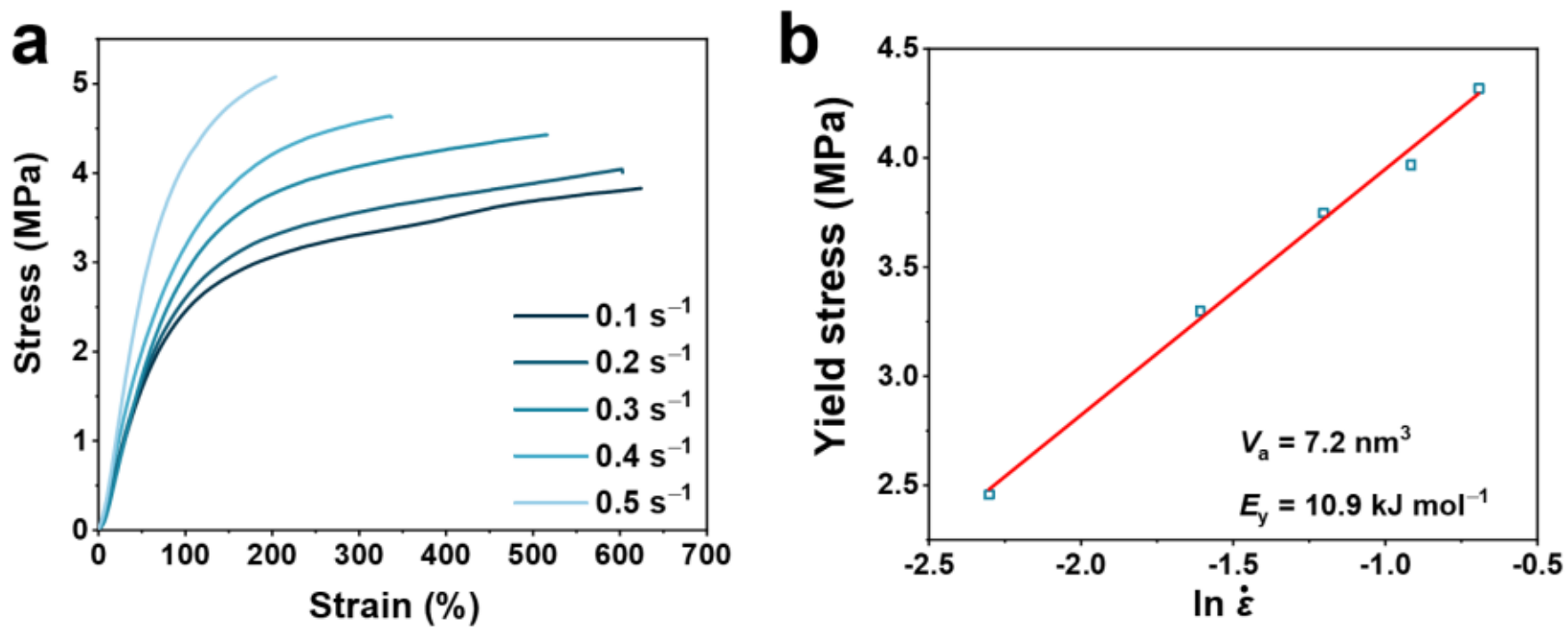

Supplementary Figure 25. Rate-dependent mechanical properties of MCH. a) Stress-strain curves of the MCH under different deformation rates. b) Fitting curves of yield stress versus deformation rate.

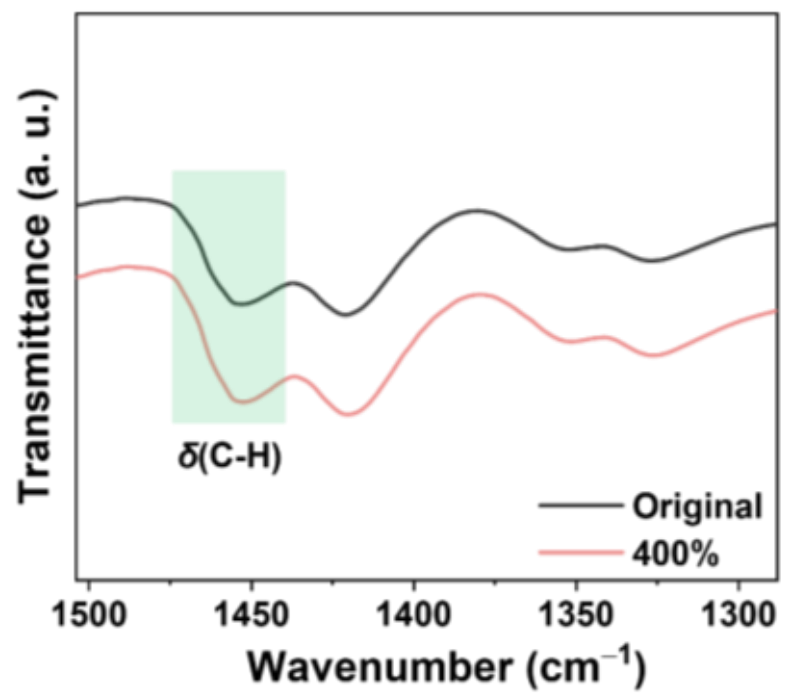

Supplementary Figure 26. Infrared spectra of δ(C-H) for IWH before and after tensile deformation. δ(C-H) remains unchanged during deformation.

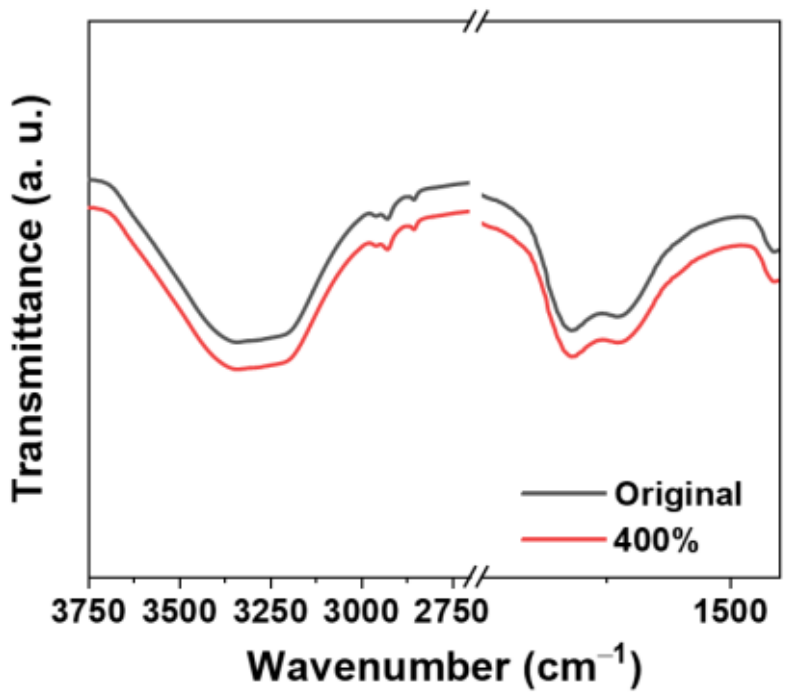

Supplementary Figure 27. Infrared spectra of the MCH before and after tensile deformation. The spectra remain unchanged during deformation.

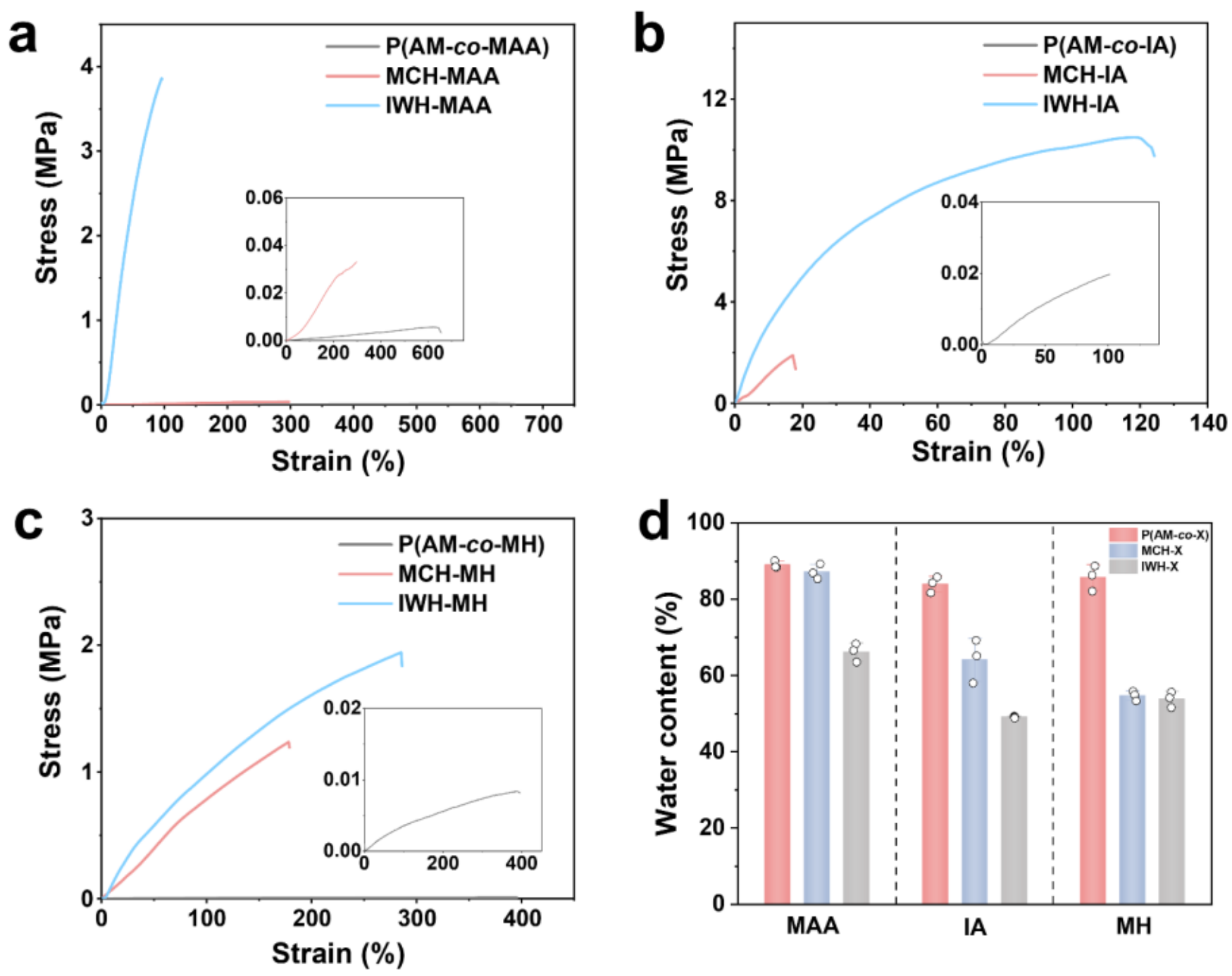

Supplementary Figure 28. Mechanical properties of different hydrogels. a) Tensile stress-strain curves of IWH-MAA, P(AM-*co*-MAA), and MCH-MAA. b) Tensile stress-strain curves of IWH-IA, P(AM-*co*-IA), and MCH-IA. c) Tensile stress-strain curves of IWH-MA, P(AM-*co*-MA), and MCH-MA. d) Water contents of IWH-MAA, IWH-IA, IWH-MH, and their control samples. Data in (d) are reported as their means ± SDs from n = 3 independent samples.

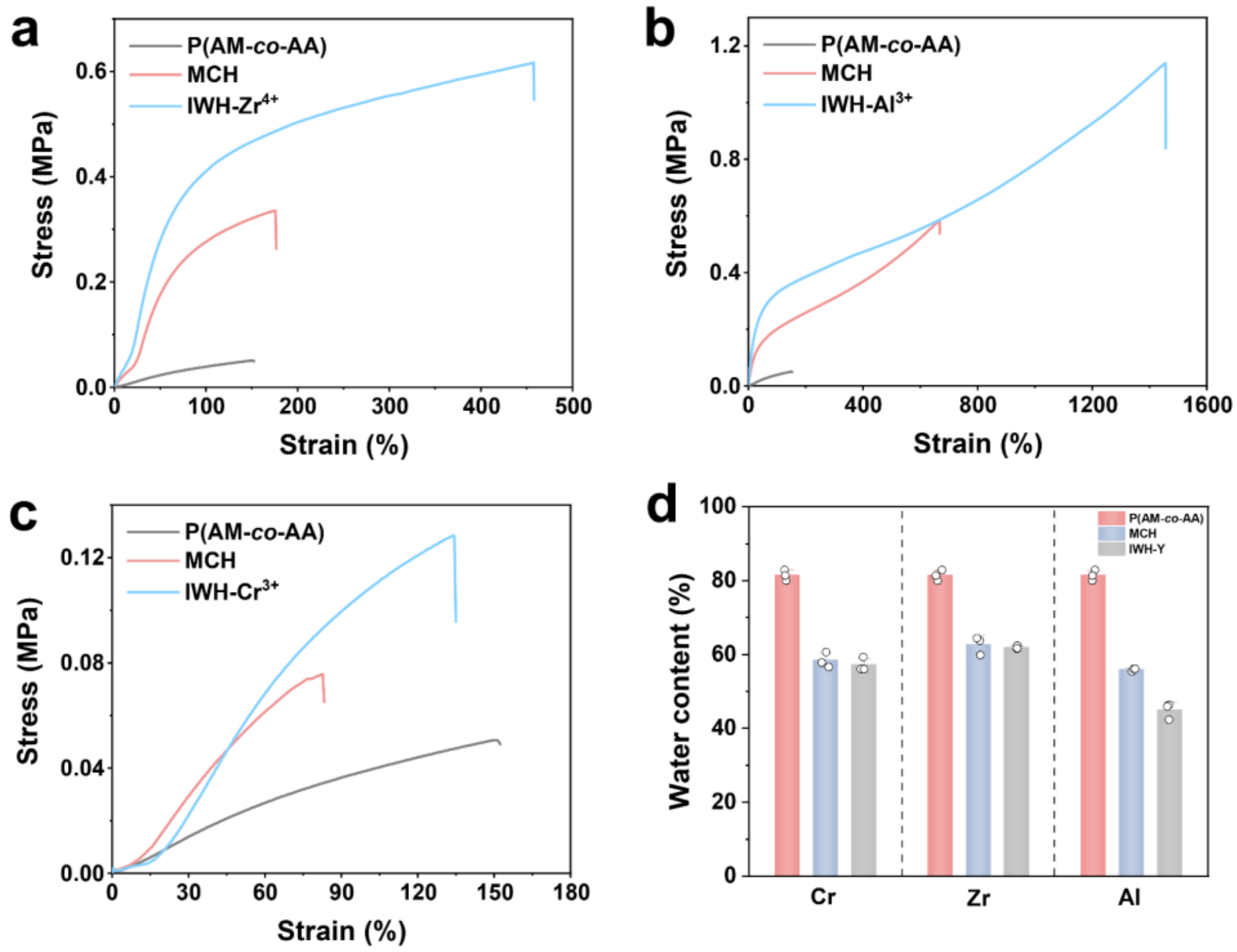

Supplementary Figure 29. Mechanical properties of different hydrogels. a) Tensile stress-strain curves of IWH-Zr^{4+}, P(AM-*co*-AA), and MCH-Zr^{4+}. b) Tensile stress-strain curves of IWH-Al^{3+}, P(AM-*co*-AA), and MCH-Al^{3+}. c) Tensile stress-strain curves of IWH-Cr^{3+}, P(AM-*co*-AA), and MCH-Cr^{3+}. d) Water contents of IWH-Zr^{4+}, IWH-Al^{3+}, IWH-Cr^{3+}, and their control samples. Data in (d) are reported as their means ± SDs from n = 3 independent samples.

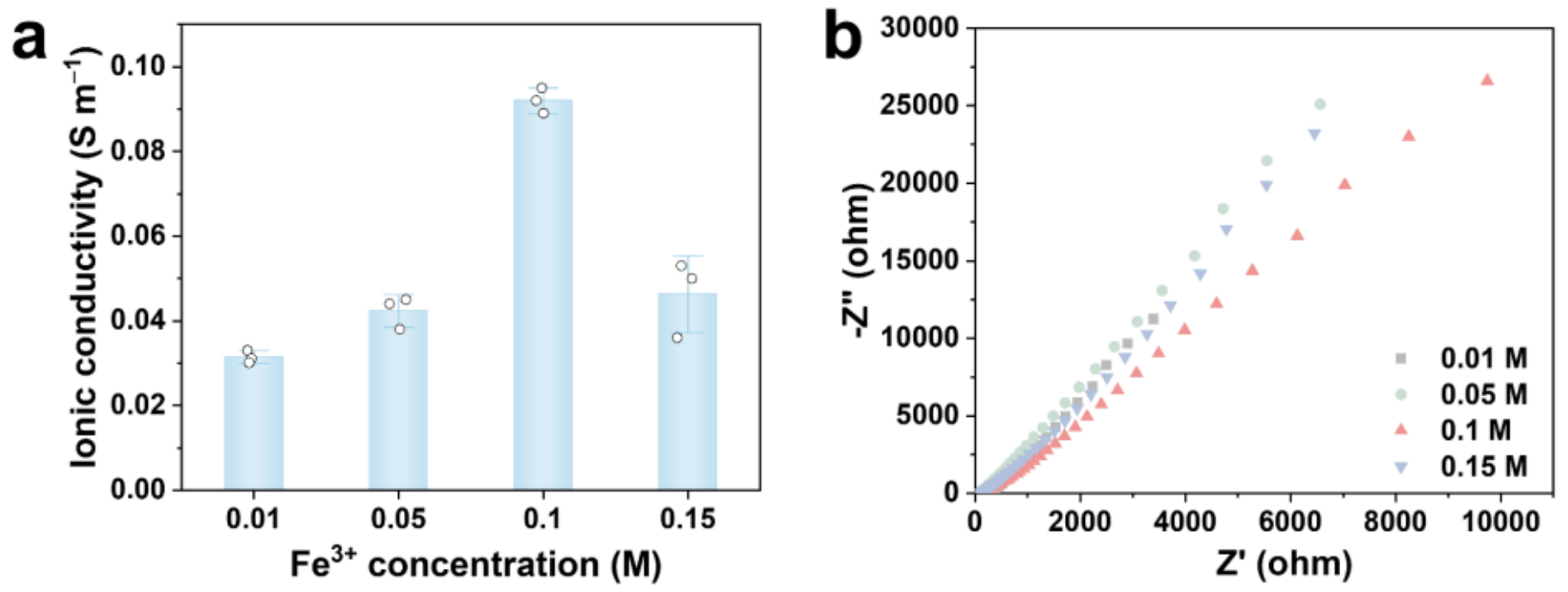

Supplementary Figure 30. Electrical properties of IWHs. a) Ionic conductivities of IWHs with different Fe^{3+} concentrations. b) Impedance spectra of IWHs with different Fe^{3+} concentrations. Data in (a) are reported as their means ± SDs from n = 3 independent samples.

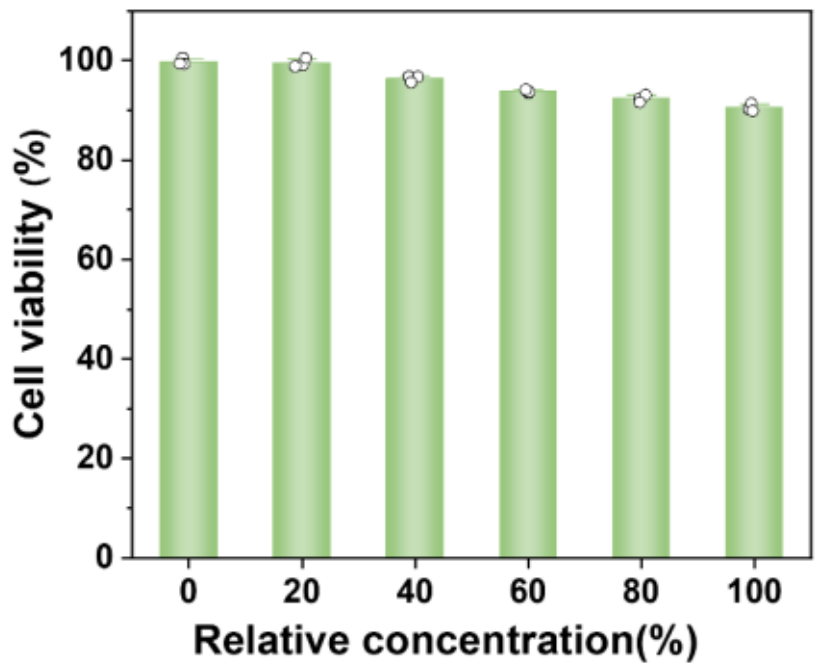

Supplementary Figure 31. Cytotoxicity test of IWH. Cell viability of mouse corneal epithelial cells incubated with different amounts of IWH for 24 h. Data are reported as their means ± SDs from n = 3 independent samples.

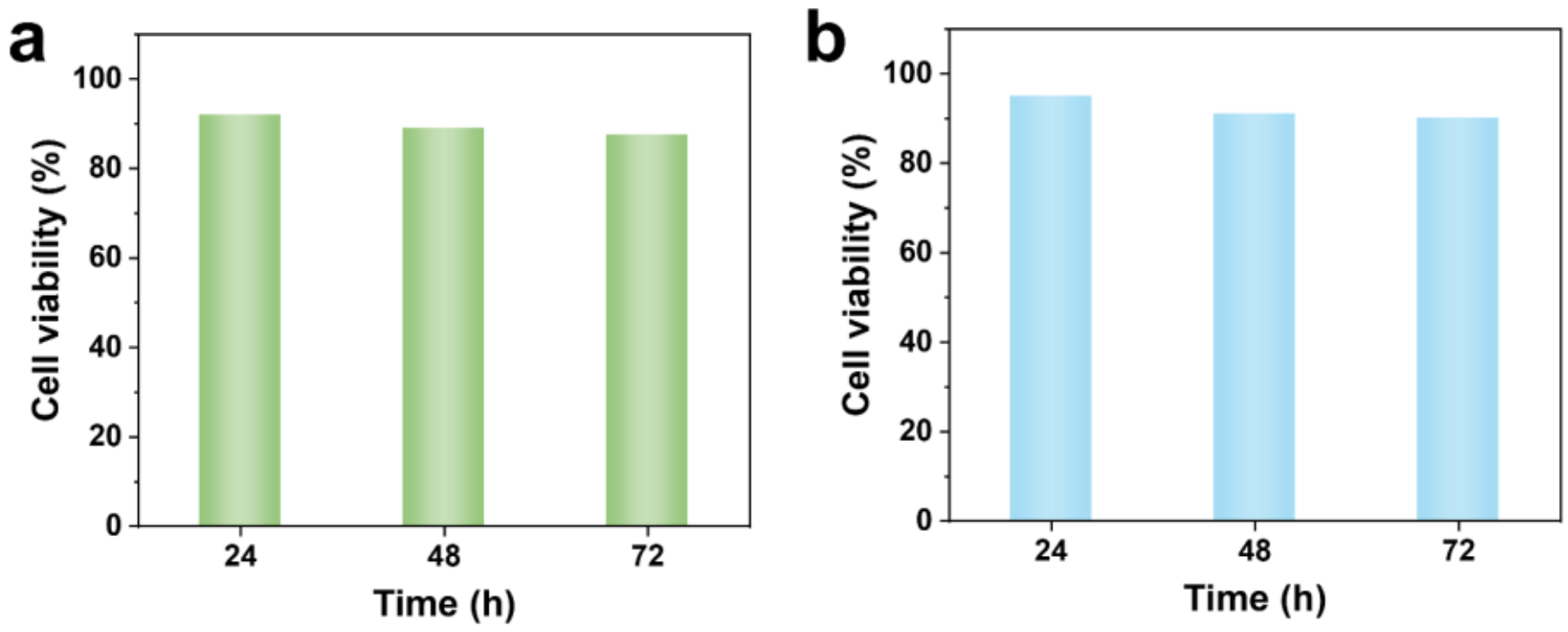

Supplementary Figure 32. Cytotoxicity test of IWH. a) Cell viability of skin fibroblasts incubated with IWH for different times. b) Cell viability of skin keratinocytes incubated with IWH for different times. The bar chart column represents a single measurement.

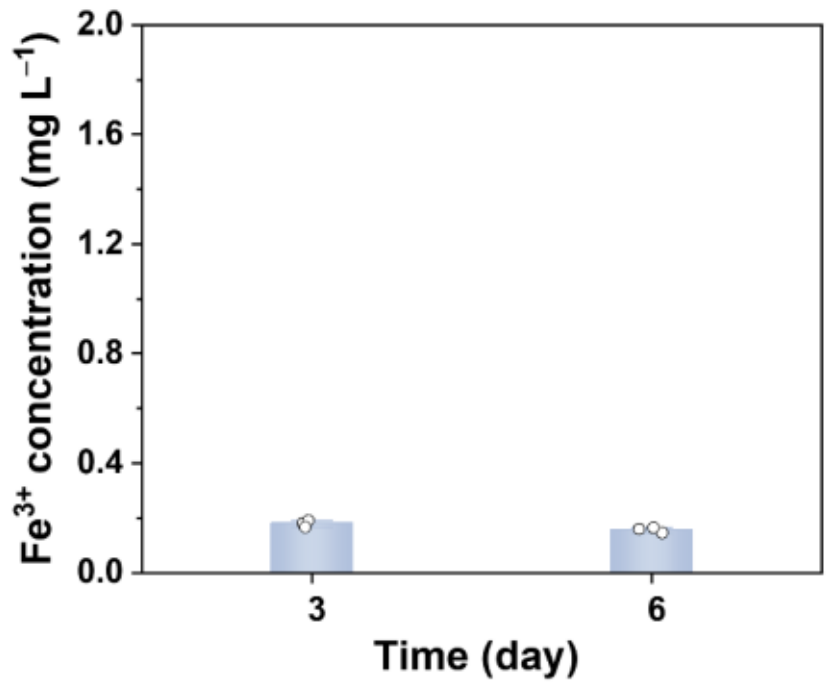

Supplementary Figure 33. Fe^{3+} concentration of the solution after immersing IWH in the simulated sweat for 3 and 6 days. The volume of the simulated sweat used during the swelling test is 100 mL, and the size of IWH is 1 cm × 1 cm × 1 mm. It is estimated that the leaching amount of Fe^{3+} is less than 1.5%. Data are reported as their means ± SDs from $n = 3$ independent samples.

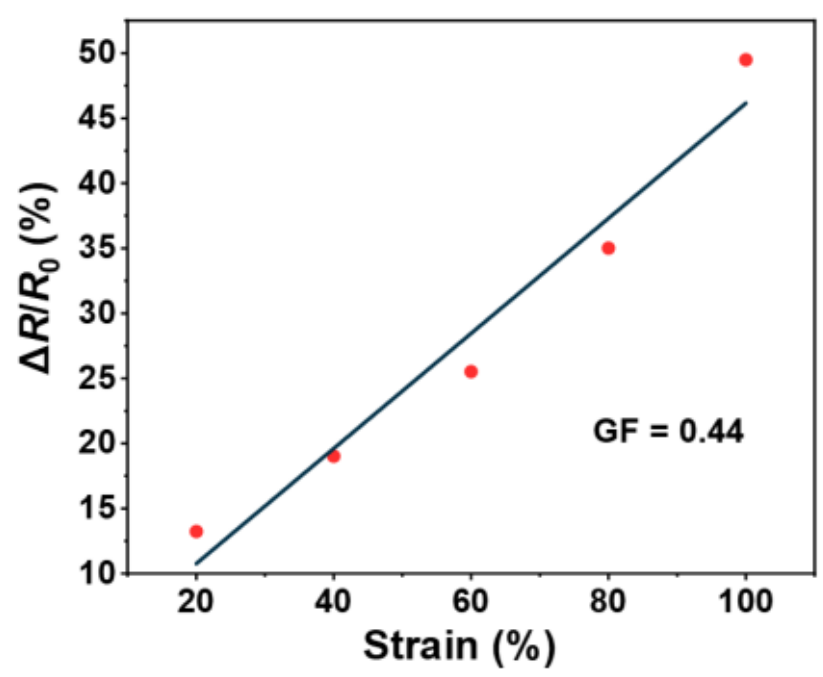

Supplementary Figure 34. Sensitivity of the IWH sensor. Resistance-strain dependence under 20%–100% strain.

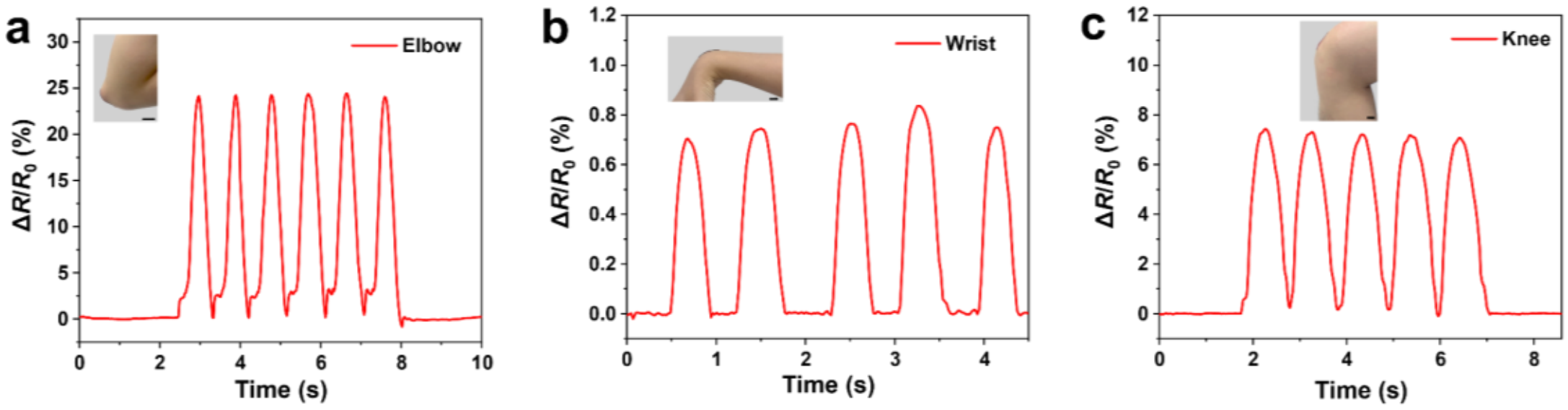

Supplementary Figure 35. Relative resistance change signals of IWH attached to different joints during bending. a) Elbow. b) Wrist. c) Knee. Scale bars: 2 cm.

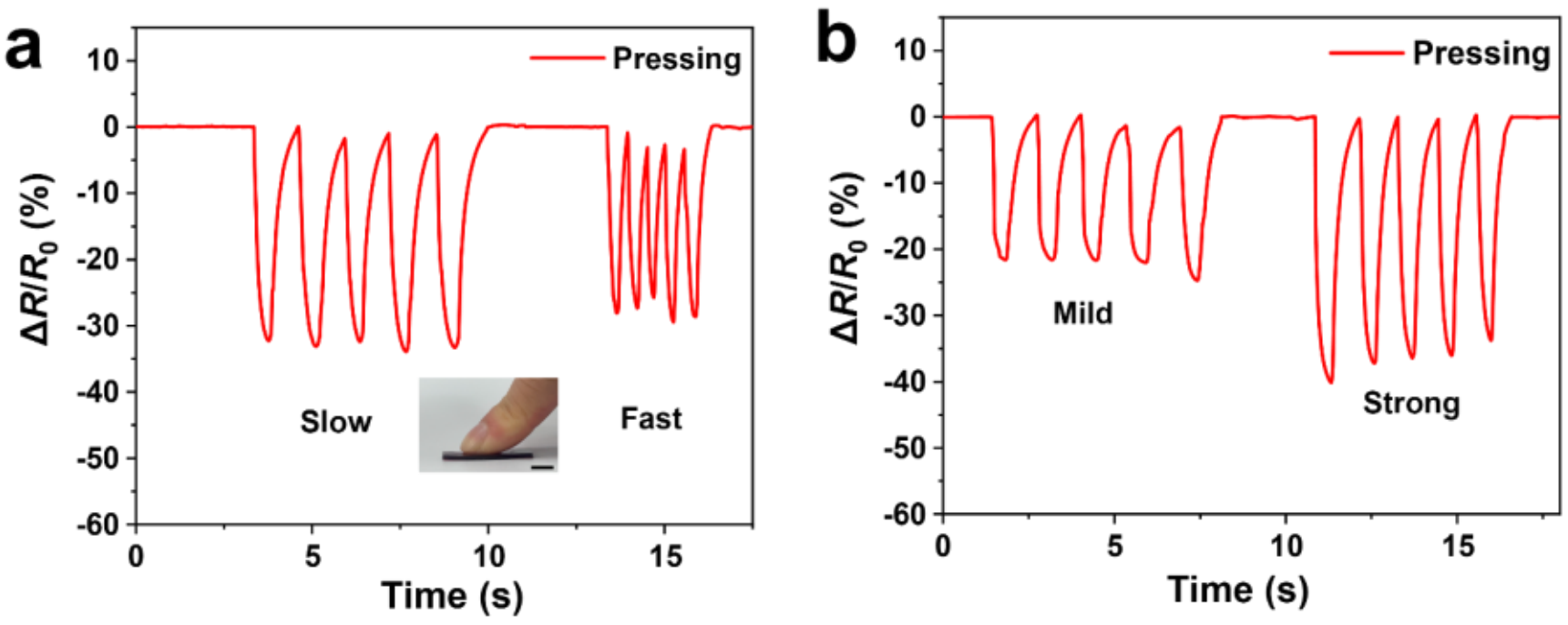

Supplementary Figure 36. Relative resistance change signals of IWH under pressing. a) Different pressing speeds. b) Different pressing amplitudes. Scale bar: 1 cm.

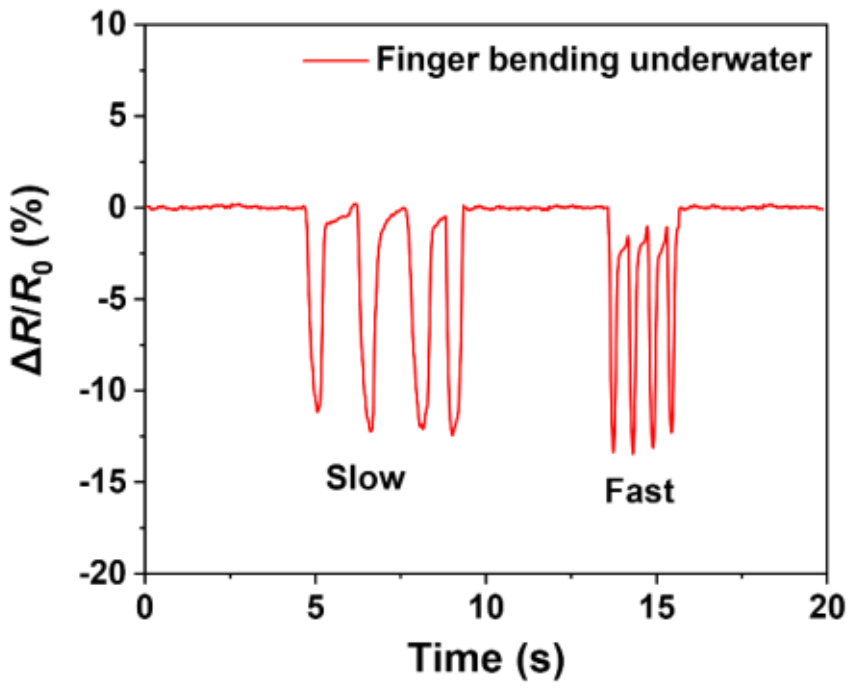

Supplementary Figure 37. Relative resistance change signals of IWH attached to a finger during underwater bending at different speeds. The sensing ability remains good underwater.

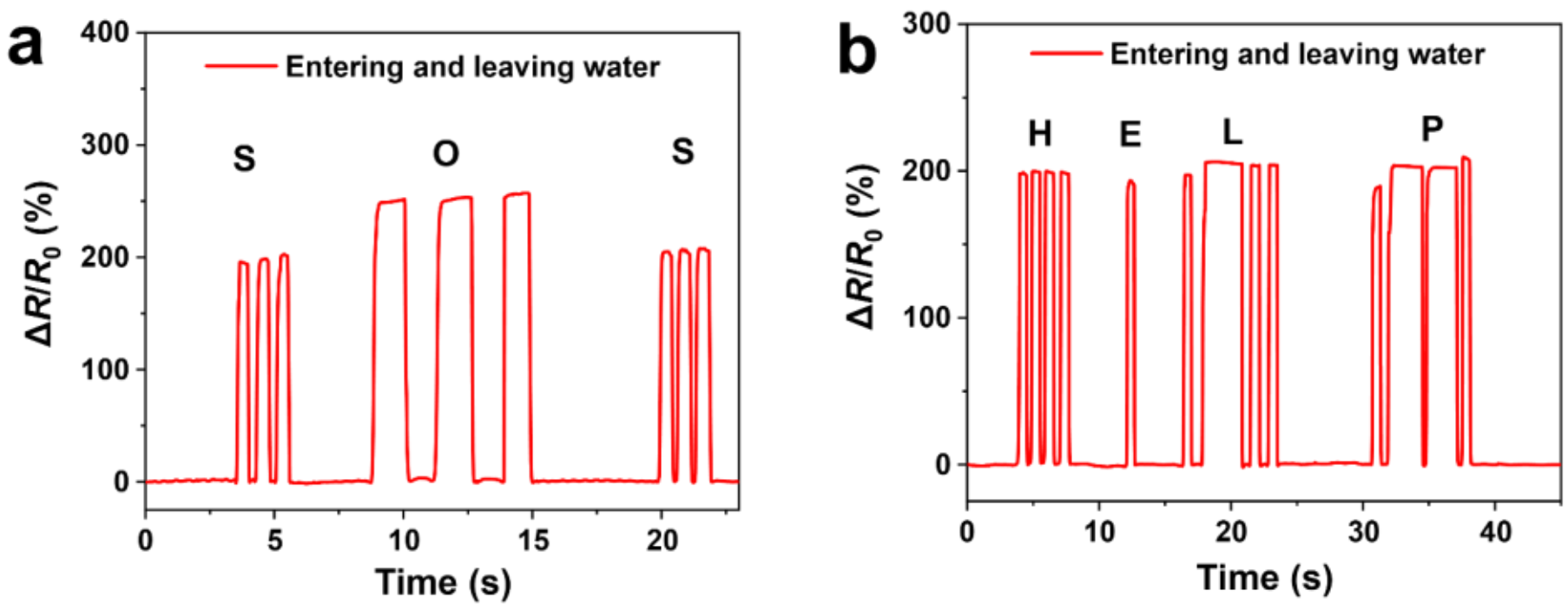

Supplementary Figure 38. Simulation of information transmission using Morse code via variable resistance signals during entering and leaving water. a) SOS. b) HELP.

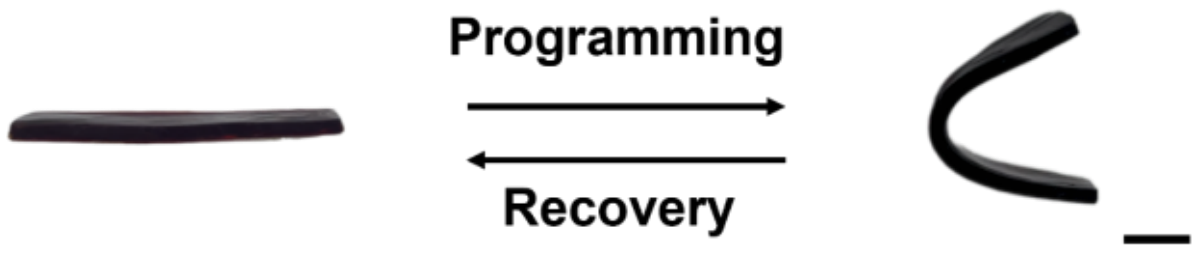

Supplementary Figure 39. Photographs of the shape memory of IWH. The sample can be programmed in ethanol and restored in water. Scale bar: 1 cm.

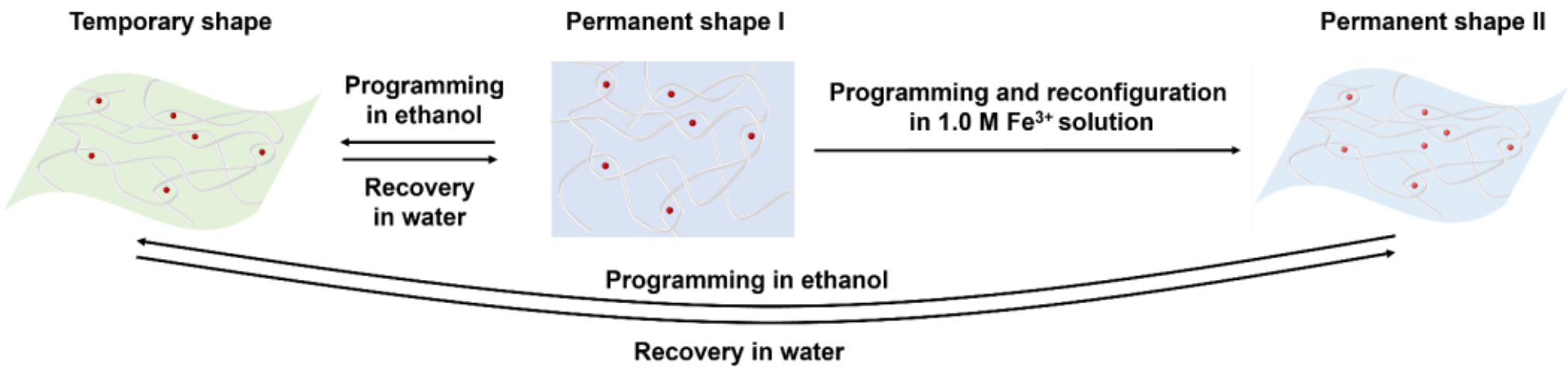

Supplementary Figure 40. Schematic illustration of the shape memory and reconfiguration processes. The IWH can be programmed by solvent stimulation and reconfigured by $FeCl_3$ solution.

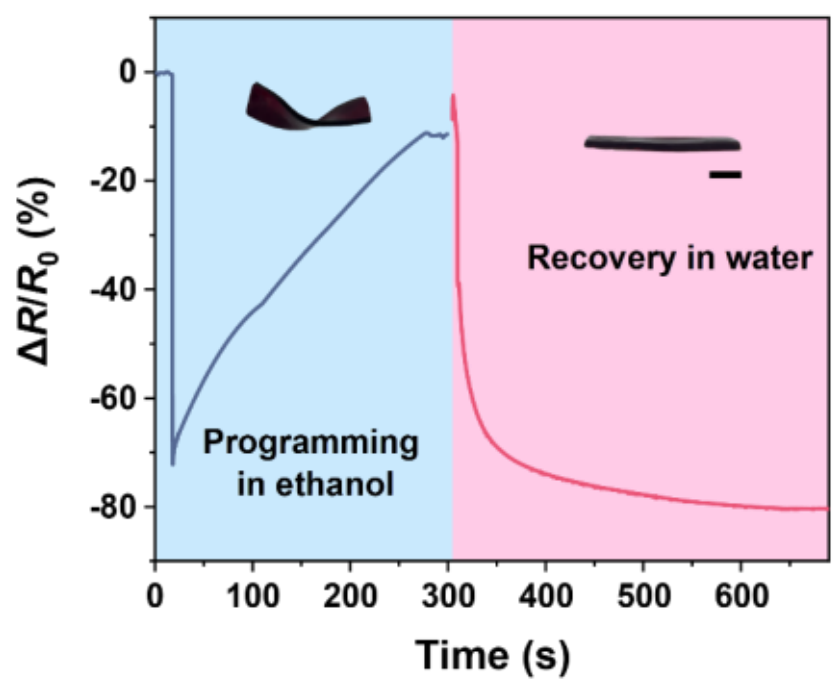

Supplementary Figure 41. Sensing signal of the shape memory process. Relative resistance change during the programming and recovery processes of IWH. Scale bar: 1 cm.

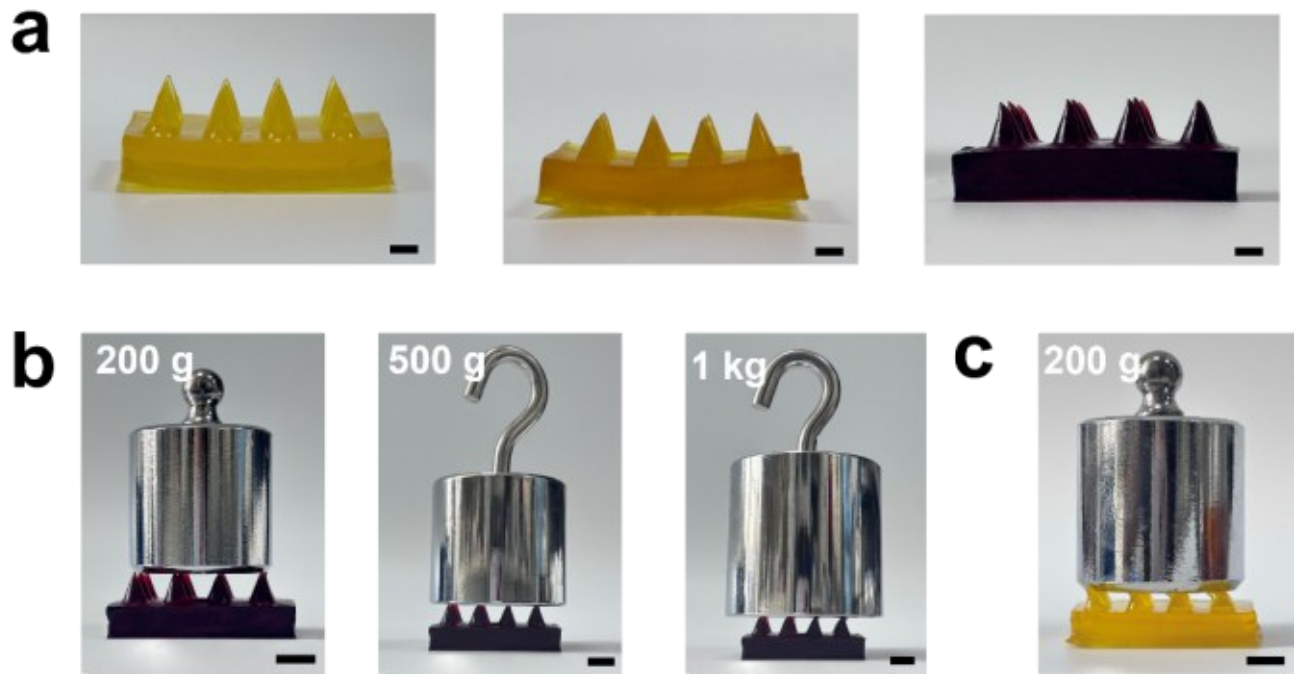

Supplementary Figure 42. 3D printing demonstration of IWH. a) Photographs of the 3D-printed P(AM-*co*-AA) hydrogel (left), dried sample (middle), and IWH (right). Scale bar: 1 cm. b) Photographs of IWH maintaining the structural integrity under 200 g, 500 g, and 1 kg loads. Scale bar: 2 cm. c) Severe deformation of the P(AM-*co*-AA) hydrogel under 200 g load. Scale bar: 2 cm.

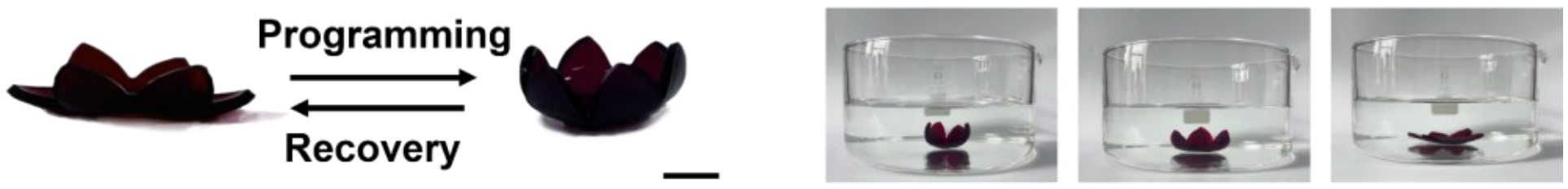

Supplementary Figure 43. 4D printing demonstration of IWH. Photographs of the 4D printing process of the flower-shaped IWH simulating blooming/closing behavior. Scale bar: 1 cm.

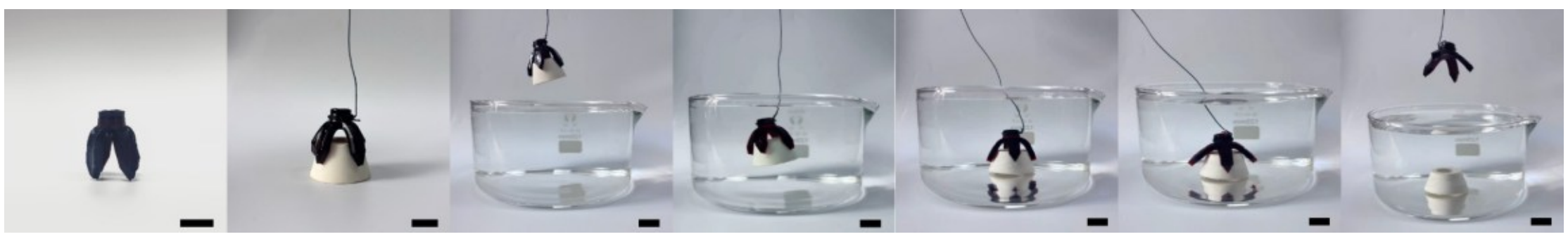

Supplementary Figure 44. Photographs of a 3D-printed claw during object grasping/releasing. The object can be grasped in the air and released in water. Scale bar: 2 cm.

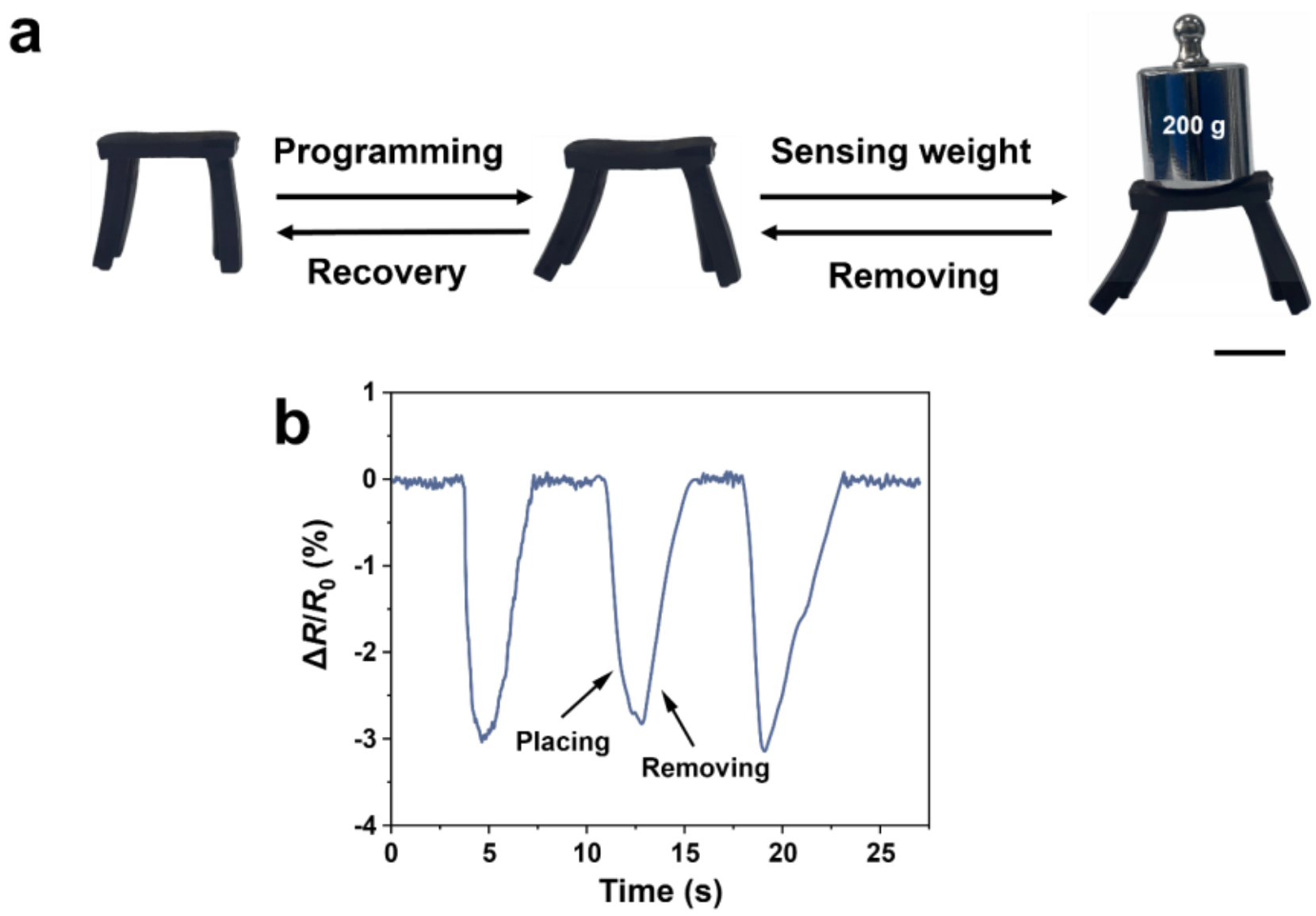

Supplementary Figure 45. Shape memory and sensing capabilities of the 3D-printed chair. a) A 3D-printed chair is programmed into the temporary state for bearing loads. Scale bar: 2 cm. b) Relative resistance change of the 3D-printed chair during bearing load.

Supplementary Table 1. Preparation parameters of P(AM-*co*-AA) hydrogels.

AA ratio	AM (g)	AA (g)	H_2O (g)	MBAA (mol%)	HMPP (mol%)
10%	1.6	0.16	16.1	0.05	0.01
15%	1.6	0.24	16.9	0.05	0.01
20%	1.6	0.32	17.7	0.05	0.01
25%	1.6	0.40	18.5	0.05	0.01

Supplementary Table 2. Comparison of the mechanical properties between this work and other metal coordination hydrogels.

Hydrogel	Stress (MPa)	Modulus (MPa)	Toughness (MJ m^{-3})	Metal ions	References
IWH	9.2	18.2	57.4	Fe^{3+}	This work
$A_{0.67}$PCL10-$G_{0.1}$	8	7.8	44.1	Fe^{3+}	*Angew. Chem. Int. Ed.* **2023**, *62*, e20231083
PAAC- Fe^{3+}	0.15	0.025	1.2	Fe^{3+}	*Adv. Sci.* **2024**, *11*, 2305702
Fe^{3+}-PAA/CNF	0.045	0.02	0.006	Fe^{3+}	*Adv. Funct. Mater.* **2025**, *35*, 2504726
FC-EtFe	6.5	1.7	0.66	Fe^{3+}	*Adv. Funct. Mater.* **2022**, *32*, 2203610

P(AAc-*co*-AAm)/NdFeB	0.85	1.4	2.04	Fe^{3+}	*Sci. Adv.* **2025**, *11*, eadw0500
CNF-DA/PAA@Fe^{3+}	0.053	0.01	0.52	Fe^{3+}	*Nat. Commun.* **2024**, *15*, 3859
PAAm-PAA/Fe^{3+}	3.4	1.75	0.0046	Fe^{3}	*Matter* **2021**, *4*, 1935
MI-HG Tanning	7.8	1.7	27.3	Zr^{4+}	*Adv. Funct. Mater.* **2024**, *34*, 2313633
PAAc-5-C-Zr^{4+}	1.35	0.3	5.2	Zr^{4+}	*Adv. Mater.* **2022**, *34*, 2204333
Zn-HA-Gel	8.8	4.4	8.8	Zn^{2+}	*Angew. Chem. Int. Ed.* **2025**, *64*, e202506449
PSZC	2	0.3	7	Zn^{2+}	*Adv. Funct. Mater.* **2024**, *34*, 2410698
PCZ-gel	0.2	0.021	1.11	Zn^{2+}	*Angew. Chem. Int. Ed.* **2023**, *62*, e202217833
C-PAMCS	0.05	0.004	0.6	Zn^{2+}	*Adv. Mater.* **2023**, *35*, 2300498
SH	0.11	0.02	0.68	Zn^{2+}	*Nat. Commun.* **2025**, *16*, 10619
PAM-hbimcp-Zn	0.19	0.095	1.18	Zn^{2+}	*Adv. Funct. Mater.* **2024**, *34*, 2314864
M-LPP	0.11	0.37	0.38	Ca^{2+}	*Adv. Mater.* **2025**, *37*, 2503910

SP-CaAc$_2$	0.5	0.1	2.1	Ca^{2+}	*Adv. Funct. Mater.* **2025**, e14481
GDL	0.14	0.075	1.37	Ca^{2+}	*Sci. Adv.* **2025**, *11*, eadz0440
MPEC	3.56	1.2	15.5	Al^{3+}	*Nat. Commun.* **2024**, *15*, 6850
HG-AlPAC	1.55	0.43	2.75	Al^{3+}	*Adv. Funct. Mater.* **2025**, *35*, 2414016
MAlgGel	1.8	2	2.6	Cu^{2+}	*Adv. Funct. Mater.* **2024**, *34*, 2410663
PAA-Fe-Li	3.3	1.2	8.4	Li^{+}	*Adv. Mater.* **2022**, *34*, 2108820
SSIH	0.013	0.45	7.35	Li^{+}	*Adv. Mater.* **2022**, *34*, 2105416
RHOCF	7.9	5.3	75	Bi^{3+}	*Nat. Commun.* **2025**, *16*, 9454
COMBIA	0.37	0.13	12.1	Bi^{3+}	*Sci. Adv.* **2025**, *11*, eadv8523

Supplementary Table 3. Comparison of anti-freezing and anti-swelling properties between this work and other hydrogels.

No.	Temperature (°C)	Swelling ratio (%)	References
	−196	0.5	**This work**
S1	−28	300	*Adv. Mater.* **2021**, *33*, 2104006
S2	−20	200	*Adv. Mater.* **2024**, *36*, 2309508
S3	−0.45	12	*Adv. Mater.* **2022**, *34*, 2108931
S4	N/A	84	*Angew. Chem. Int. Ed.* **2021**, *60*, 25856
S5	−13	−3	*Nat. Commun.* **2025**, *16*, 6221
S6	−20	700	*Adv. Mater.* **2022**, *34*, 2205344
S7	−46	200	*Adv. Mater.* **2025**, *37*, 2502850
S8	N/A	120	*Nat. Commun.* **2024**, *15*, 1587
S9	N/A	131	*Angew. Chem. Int. Ed.* **2024**, *63*, e202406407
S10	−80	N/A	*Adv. Mater.* **2023**, *35*, 2211673

Supplementary Table 4. Comparison of functional characteristics between this work and other materials.

No.	Type	Conductivity	Sensing	Dehydration-rehydration	Reconfigurable shape memory	3D printing	Self-sensing 4D printing	References
	Hydrogel	Yes	Yes	Yes	Yes	Yes	Yes	This work
S11	Hydrogel	Yes	Yes	No	No	No	No	*Science* **2024**, *384*, 557
S12	Hydrogel	Yes	Yes	No	No	No	No	*Angew. Chem. Int. Ed.* **2025**, *64*, e202514750
S13	Hydrogel	Yes	Yes	No	No	No	No	*Sci. Adv.* **2025**, *11*, eadv8523
S14	Hydrogel	Yes	Yes	No	No	Yes	No	*Nat. Commun.* **2025**, *16*, 185
S15	Hydrogel	Yes	Yes	No	No	Yes	No	*Nat. Electron.* **2022**, *5*, 893
S16	Hydrogel	Yes	No	No	No	Yes	No	*Nat. Commun.* **2024**, 15, 6850
S17	Hydrogel	Yes	Yes	No	No	Yes	No	*Adv. Mater.* **2022**, *34*, 2203650
S18	Hydrogel	Yes	Yes	No	No	Yes	No	*Adv. Mater.* **2023**, *35*, 2307070
S19	Hydrogel	Yes	Yes	No	No	Yes	No	*Nat. Commun.* **2023**, *14*, 4289
S20	Hydrogel	No	No	No	Yes	No	No	*Adv. Mater.* **2026**, *38*, e11014
S8	Hydrogel	No	No	Yes	No	Yes	No	*Nat.Commun.* **2024**, *5*, 1587
S21	Hydrogel	No	No	Yes	No	No	No	*Science* **2020**, *370*, 335
S22	Elastomer	No	No	No	No	Yes	No	*Nature* **2024**, *631*, 783
S23	Elastomer	No	No	No	Yes	Yes	No	*Sci. Adv.* **2024**, *10*, eadl4387
S24	Ionogel	Yes	Yes	No	Yes	No	No	*Sci. Adv.* **2024**, *10*, eadl2737

Supplementary Table 5. Preparation parameters of P(AM-*co*-MAA), P(AM-*co*-IA), and P(AM-*co*-MH) hydrogels.

Monomer	AM (g)	Carboxyl-containing monomer (g)	H_2O (g)	MBAA (mol%)	HMPP (mol%)
MAA	1.6	0.78	21.45	0.05	0.01
IA	1.6	0.88	22.32	0.05	0.01
MH	1.6	0.78	21.45	0.05	0.01

Supplementary references

S1. Zhang, D.*, et al.* A general crosslinker strategy to realize intrinsic frozen resistance of hydrogels. *Adv. Mater.* **33**, 2104006 (2021).

S2. Liu, X., Ji, X., Zhu, R., Gu, J., & Liang, J. A microphase-separated design toward an all-round ionic hydrogel with discriminable and anti-disturbance multisensory functions. *Adv. Mater.* **36**, 2309508 (2024).

S3. Luo, Z.*, et al.* Vertical extrusion cryo(bio)printing for anisotropic tissue manufacturing. *Adv. Mater.* **34**, 2108931 (2022).

S4. Macdougall, L. J.*, et al.* Using stereochemistry to control mechanical properties in thiol-yne click-hydrogels. *Angew. Chem. Int. Ed.* **60**, 25856-25864 (2021).

S5. Huo, H.*, et al.* A tough and robust hydrogel constructed through carbon dots induced crystallization domains integrated orientation regulation. *Nat. Commun.* **16**, 6221 (2025).

S6. Lu, H.*, et al.* Tailoring the desorption behavior of hygroscopic gels for atmospheric water harvesting in arid climates. *Adv. Mater.* **34**, 2205344 (2022).

S7. Wu, Q., Chauhan, M., Khamaisi, B., Nassar-Marjiya, E., & Farah, S. Biomimetic 3d-printed adaptive hydrogel bioadhesives featuring superior infection resistance for challenging tissue adhesion, hemostasis, and healthcare. *Adv. Mater.* **37**, 2502850 (2025).

S8. Liu, B.*, et al.* 4d printed hydrogel scaffold with swelling-stiffening properties and programmable deformation for minimally invasive implantation. *Nat. Commun.* **15**, 1587 (2024).

S9. Yang, B.*, et al.* Strong acid enabled comprehensive training of poly (sodium acrylate) hydrogel networks. *Angew. Chem. Int. Ed.* **63**, e202406407 (2024).

S10. Yan, Y.*, et al.* Tough hydrogel electrolytes for anti-freezing zinc-ion batteries. *Adv. Mater.* **35**, e2211673 (2023).

S11. Li, P.*, et al.* N-type semiconducting hydrogel. *Science* **384**, 557-563 (2024).

S12. Liu, G.*, et al.* Energy dissipation mediated by multiple noncovalent interactions in

hydrogen‐bonded organic frameworks‐based hydrogels for wearable gesture‐to‐recognition translation. *Angew. Chem. Int. Ed.* **64**, 2514750 (2025).

S13. Guo, X., *et al.* A skin-mimicking multifunctional hydrogel via hierarchical, reversible noncovalent interactions. *Sci. Adv.* **11**, eadv8523 (2025).

S14. Li, J., *et al.* Multimaterial cryogenic printing of three-dimensional soft hydrogel machines. *Nat. Commun.* **16**, 185 (2025).

S15. Hui, Y., *et al.* Three-dimensional printing of soft hydrogel electronics. *Nat. Electron.* **5**, 893-903 (2022).

S16. Lee, S., *et al.* Molecular control via dynamic bonding enables material responsiveness in additively manufactured metallo-polyelectrolytes. *Nat. Commun.* **15**, 6850 (2024).

S17. Shen, Z., *et al.* High‐stretchability, ultralow‐hysteresis conductingpolymer hydrogel strain sensors for soft machines. *Adv. Mater.* **34**, 2203650 (2022).

S18. Kim, S., *et al.* Injection‐on‐skin granular adhesive for interactive human–machine interface. *Adv. Mater.* **35**, 2307070 (2023).

S19. Xie, X., Xu, Z., Yu, X., Jiang, H., Li, H., & Feng, W. Liquid-in-liquid printing of 3d and mechanically tunable conductive hydrogels. *Nat. Commun.* **14**, 4289 (2023).

S20. Shan, L., *et al.* Mechanically compliant and impedance matching hydrogel bioelectronics for low‐voltage peripheral neuromodulation. *Adv. Mater.* **38**, e11014 (2026).

S21. Lin, W., *et al.* Cartilage-inspired, lipid-based boundary-lubricated hydrogels. *Science* **370**, 335-338 (2020).

S22. Fang, Z., *et al.* 3d printable elastomers with exceptional strength and toughness. *Nature* **631**, 783-788 (2024).

S23. Li, H., *et al.* Reconfigurable 4d printing via mechanically robust covalent adaptable network shape memory polymer. *Sci. Adv.* **10**, eadl4387 (2024).

S24. Zhao, Z., *et al.* Bicontinuous vitrimer heterogels with wide-span switchable stiffness-gated iontronic coordination. *Sci. Adv.* **10**, eadl2737 (2024).
