# Supplementary material for: Ionic weaving network enables mechanically robust smart hydrogels with anti-swelling and anti-freezing capabilities
Source: Nat Commun. 2026 Jul 29;17:9191. doi: 10.1038/s41467-026-76165-4 (PMC13522580; doi:10.1038/s41467-026-76165-4)
Supplement: Supplementary file 2 [file 41467_2026_76165_MOESM2_ESM.pdf]

nature portfolio

Peer Review File

Ionic weaving network enables mechanically robust smart hydrogels with anti-swelling and anti-freezing capabilities

Corresponding Author: Professor Xiaolin Lyu

This file contains all reviewer reports in order by version, followed by all author rebuttals in order by version.

Version 0:

Reviewer comments:

Reviewer #1

(Remarks to the Author)
This work presents an innovative ionic weaving strategy for fabricating high-performance hydrogels with exceptional mechanical properties, environmental stability, and integrated sensing-actuation capabilities. The restricted drying pre-treatment combined with uniform metal ion coordination successfully constructs a 3D topological network, addressing critical limitations of traditional hydrogels such as poor mechanical strength, swelling in water, and frost damage at low temperatures. The integration of ionic conductivity enables versatile sensing applications like underwater communication, while the solvent-induced shape memory effect and 3D/4D printing compatibility demonstrate great potential for intelligent soft robotics. The study is well-designed, and the conclusion has scientific significance. Overall, this work provides a novel and universal approach for developing next-generation multifunctional smart hydrogels, which is suitable for publication in Nature Communications. However, there are still some minor revisions to be addressed.
1. The specific environmental conditions for the restricted drying step, such as temperature and humidity, are not explicitly stated. These parameters are crucial for reproducibility.
2. Meanwhile, it is important to clarify how variations in environmental parameters affect the performance of IWH.
3. The reconfigurable capability of shape memory is interesting. I suggest using schematic diagrams to better illustrate this reconfiguration process on the molecular scale, as the mechanism of ion coordination reconfiguration is distinctly different from that of common dynamic covalent bond networks.
4. The shape memory process or 4D printing process can be monitored via electrical signals, which is also a significant highlight of this research. The underlying principle lies in the correlation between the deformation degree of the hydrogel and its electrical resistance. Additionally, it seems that the IWH exhibits different electrical resistances or conductivities in various solvents. It is recommended to provide a more detailed explanation in the relevant section regarding the actuation-sensing integration for readers to better understand.
5. Some data are expressed with slight inconsistencies, such as the mechanical attributes of IWH in the manuscript and supplementary materials. The author should carefully review the entire paper to unify the data.

Reviewer #2

(Remarks to the Author)
This study introduces an ionic weaving method to develop multifunctional hydrogels, which possess mechanical robustness, anti-freezing, and anti-swelling properties. Thus, the hydrogel can be used in applications including underwater sensing and intelligent 4D printing with self-sensing capabilities. This work is experimentally comprehensive and demonstrates impressive macroscopic performance. In summary, the topic is innovative, and the results are interesting. The manuscript can be published in Nature Communications after solving the following concerns.

1. The authors indicate that ionic weaving can enhance the stability of polymer networks and dissipate energy through synchronous dissociation. At present, the authors have only investigated the hysteresis area and damping capacity of IWH. To better illustrate the contribution of ionic weaving networks to energy dissipation, it is suggested to supplement the cyclic stress-strain curves of the control group and compare their hysteresis area and damping capacity with those of IWH, thereby supporting the conclusion above.
2. In the calculation of energy density for the shape memory process, what was the pre-stretching strain of the hydrogel?

3. Why did the water content of the precursor solution need to be reduced to 60% in 3D printing?
4. Why was the initiator in 3D printing replaced from HMPP to LAP?
5. How long did it take for the programmed IWH to fully recover its original shape after being immersed in water?
6. The reconfigurable shape memory behavior of IWH was set by re-immersion in salt solution. What are the advantages or significance of the solution-based reconfigurable process compared to the thermal reconfigurable process based on other polymers?
7. Some figures in the manuscript (such as Fig. 4g) have scale bars with excessively small font sizes. Some photographs do not have scale bars. These need to be revised.

Reviewer #3

(Remarks to the Author)
This manuscript proposes a strategy termed ionic weaving hydrogels (IWHs). Specifically, an AM/AA copolymer hydrogel is first subjected to restricted drying (four-edge constrained drying), followed by Fe^{3+} incorporation to generate dynamic coordination crosslinks. The authors claim that this process yields a uniformly distributed three-dimensional topological "weaving" network, which concomitantly enhances strength, modulus, and toughness. The resulting hydrogels are further reported to exhibit minimal swelling, anti-freezing capability, and recoverable performance after dehydration–rehydration cycling, and the work additionally demonstrates 4D printing and self-sensing applications. The manuscript's core claims, specifically the formation of a uniform 3D "ionic weaving" network and its role in coordination-driven toughness and anti-swelling, are not supported by sufficient direct evidence. Critical baselines and controls (e.g., 0% AA, Fe-free, dry-only) are missing, making it impossible to decouple Fe^{3+} coordination effects from processing artifacts such as restricted-drying–induced densification, water-content changes, and anisotropy. Furthermore, the boundary conditions for the restricted-drying process are underdefined, which undermines reproducibility. The extraordinary anti-swelling results lack adequate methodological detail, statistical results, and controls to rule out Fe^{3+} leaching, surface-shell formation, or ionic screening. Mechanical characterization relies heavily on tensile curves without fracture-level testing, and the temperature-dependent coordination dynamics remain unvalidated due to the absence of variable-temperature rheology and in situ spectroscopy. Finally, the biocompatibility evidence provided is too preliminary to support the claims regarding wearable or biomedical applications. I can reject this manuscript to be published in this journal. Some other comments are provided as follows:

1. The attribution of mechanical performance to dynamic Fe^{3+}–carboxylate coordination is currently under-supported. To distinguish true coordination dynamics from processing artifacts (e.g., drying), "temperature-dependent rheology" ($G'/G''/\tan\delta$ vs. T in the LVE regime) is essential. Furthermore, claims of reversible dissociation require verification via in situ variable-temperature FTIR rather than ex situ measurements. Correlating spectral changes with rheological relaxation is necessary to rigorously establish the structure–dynamics–property relationship.

2.The claim of a "uniformly distributed 3D topological weaving" network is unsubstantiated. Current characterizations (SAXS, XPS, SEM) capture only average or surface features and fail to rule out diffusion-induced gradients; depth-resolved measurements are required to verify bulk uniformity. Furthermore, the term "weaving" lacks a clear operational definition. Quantitative metrics are needed to strictly distinguish this specific topology from generic effects like densification or increased crosslink density.

3. The experimental design suffers from missing baselines and ambiguous nomenclature. Supplementary Figs. 1 and 6 require AM-only (0% AA) and Fe-free references to isolate chemical contributions. Crucially, a "restricted drying without Fe^{3+}" control is missing, making it impossible to decouple coordination strengthening from processing-induced densification. Figure 1 and terms like "Control" must be revised to explicitly map sample names to their precise composition and processing history (e.g., restricted drying, Fe^{3+}).

4. The extraordinary anti-swelling claim (~0.5% vs. ~7225%) lacks rigorous validation. Key experimental parameters (sample geometry, surface-to-volume ratio, pH/ionic strength) are undefined. Crucially, the current data fails to distinguish a true bulk network from kinetic suppression via a surface "shell" or Fe^{3+} leaching. The authors must provide thickness-dependent swelling kinetics, quantified Fe leaching (ICP-MS), and cross-sectional mapping to confirm bulk uniformity. Additionally, statistical data (n>=3, mean SD) and visual documentation are required to ensure reproducibility.

5. The current biocompatibility assessment (24 h MTT, single cell line) is insufficient to support wearable/biomedical applications. To validate safety, time-dependent Fe^{3+} leaching must be quantified via ICP-OES/MS in relevant media (e.g., simulated sweat). Additionally, cytotoxicity testing requires expansion to skin-relevant cells (fibroblasts/keratinocytes) over multiple time points (24–72 h). Without these data, biomedical claims must be significantly moderated to "preliminary screening."

6. The "restricted drying" process, crucial for the "ionic weaving" mechanism, lacks sufficient detail to ensure reproducibility. The manuscript fails to define key boundary conditions, specifically: ambient parameters (temperature, humidity, airflow), specimen geometry/constraints (dimensions, clamping, applied tension), and the drying endpoint criteria. Given the sensitivity of this method, video documentation of the setup and long-term time-lapse observations are required to capture the full drying evolution and distinguish robust properties from environment-dependent processing artifacts.

7. The reliance on tensile toughness (area under the curve) is inadequate, as this metric is often flaw-insensitive. To validate the "3D topological weaving" mechanism, fracture-level characterization (e.g., notch/tear tests, fracture energy) is required to quantify actual crack resistance and defect tolerance.

Version 1:

Reviewer comments:

Reviewer #1

(Remarks to the Author)
The authors replied to my comments and questions properly, which satisfied me sufficiently. I think that it can be published in Nature Communications now.

Reviewer #2

(Remarks to the Author)
The authors have adequately revised their manuscript. I have no further questions.

Reviewer #3

(Remarks to the Author)
The revised manuscript has been substantially improved. The added Fe-free, AA-free, and dry-only controls, together with the clarified restricted-drying conditions, strengthen the mechanistic discussion and reproducibility. The additional fracture-related tests, Fe^{3+} leaching analysis, and cytotoxicity experiments also improve the overall quality of the work.
However, several issues still require revision.
First, the temperature-dependent coordination dynamics remain insufficiently validated. The step-strain rheology and in situ tensile FTIR support deformation-induced dissociation/reformation of Fe^{3+}–carboxylate coordination, but they do not fully replace temperature-dependent rheology or variable-temperature spectroscopy. The authors should clearly acknowledge this limitation and avoid implying that temperature-dependent coordination dynamics have been directly verified.
Second, the Introduction should refine the definition of the literature gap. The current discussion attributes hydrogel environmental instability mainly to uneven interchain interactions and heterogeneous phase-separated structures. This explanation is somewhat oversimplified, as anti-swelling and anti-freezing behaviors may also be affected by water state, osmotic pressure, ion leaching, solvent exchange, network charge density, and polymer–water interactions.
Finally, the biocompatibility claims should be moderated. The current data support only preliminary skin-contact compatibility for wearable applications, not broad biomedical safety or biological tissue compatibility.
Overall, I recommend borderline acceptance after substantial revision.

Version 2:

Reviewer comments:

Reviewer #3

(Remarks to the Author)
The authors have already addressed my comments.

Open Access This Peer Review File is licensed under a Creative Commons Attribution 4.0 International License, which permits use, sharing, adaptation, distribution and reproduction in any medium or format, as long as you give appropriate credit to the original author(s) and the source, provide a link to the Creative Commons license, and indicate if changes were made.
In cases where reviewers are anonymous, credit should be given to 'Anonymous Referee' and the source.
The images or other third party material in this Peer Review File are included in the article's Creative Commons license, unless indicated otherwise in a credit line to the material. If material is not included in the article's Creative Commons license and your intended use is not permitted by statutory regulation or exceeds the permitted use, you will need to obtain permission directly from the copyright holder.
To view a copy of this license, visit https://creativecommons.org/licenses/by/4.0/

Response to Reviewers

We are appreciative of the feedback from our peer reviewers. Their constructive comments have significantly contributed to enhancing our manuscript. In response to their concerns, we have expanded our research with additional experiments, analyses, and discussions. These improvements are thoroughly integrated into the revised manuscript.

To facilitate a clear understanding of our revisions, we have organized our responses as follows:

Reviewer Comments and Our Responses: Each comment from the Reviewers (presented in black text) is followed by our corresponding response (in blue text).

Highlighted Text in the Revised Manuscript: Text modifications in the revised manuscript are red in the response letter.

Response to the Reviewer #1

Comment: This work presents an innovative ionic weaving strategy for fabricating high-performance hydrogels with exceptional mechanical properties, environmental stability, and integrated sensing-actuation capabilities. The restricted drying pre-treatment combined with uniform metal ion coordination successfully constructs a 3D topological network, addressing critical limitations of traditional hydrogels such as poor mechanical strength, swelling in water, and frost damage at low temperatures. The integration of ionic conductivity enables versatile sensing applications like underwater communication, while the solvent-induced shape memory effect and 3D/4D printing compatibility demonstrate great potential for intelligent soft robotics. The study is well-designed, and the conclusion has scientific significance. Overall, this work provides a novel and universal approach for developing next-generation multifunctional smart hydrogels, which is suitable for publication in Nature Communications. However, there are still some minor revisions to be addressed.

Reply: We are very grateful to the Reviewer for the encouraging comments on our manuscript. We have revised the manuscript and hope to address the issues raised by

the Reviewer.

Question 1: The specific environmental conditions for the restricted drying step, such as temperature and humidity, are not explicitly stated. These parameters are crucial for reproducibility.

Answer 1: Thanks for the Reviewer's suggestion. The restricted drying step was performed at room temperature (25 °C) with a relative humidity of 60%. The environmental parameters have been added to the experimental section for the reproducibility of the experiments.

We supplement the experimental parameters on Page S3 of the revised supplementary information.

"Its four sides were secured with clamps onto a glass slide without pre-applied tension, and it was left to dry naturally in an indoor environment (25 °C, 60% RH, natural convection) for one day."

Question 2: Meanwhile, it is important to clarify how variations in environmental parameters affect the performance of IWH.

Answer 2: Thanks for the Reviewer's suggestion. We investigated the effect of different environmental parameters, such as temperature and humidity, on the mechanical properties of IWH. The results show that whether the temperature is varied (*e.g.*, higher temperatures of 40 °C and 60 °C) or the humidity is changed (*e.g.*, 30% RH and 80% RH), the resulting IWHs exhibit similar mechanical properties. This indicates that the improvement in the mechanical properties of IWH is primarily dependent on the unique restricted drying step and is less influenced by environmental parameters.

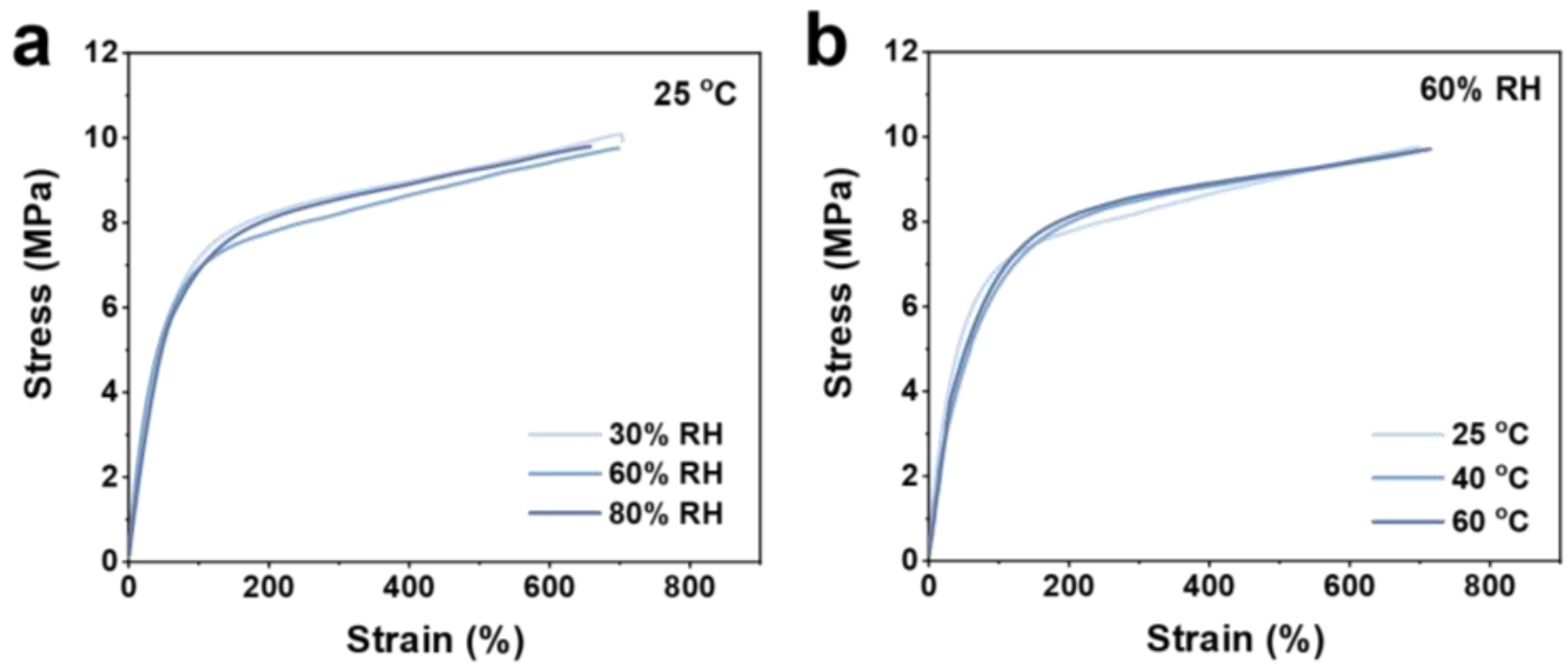

Supplementary Figure 9. Effect of restricted drying under different environmental conditions on the mechanical properties of IWHs. Stress-strain curves of IWHs prepared under different humidities (a) and different temperatures (b).

We supplement the discussion on Page 10 of the revised manuscript.

"We further explored the influence of different environmental conditions during restricted drying on the mechanical properties of IWH. Similar mechanical properties are observed under different temperatures and humidities, revealing that the improvement in the mechanical properties of IWH is primarily dependent on the unique restricted drying step and is less influenced by environmental parameters (Supplementary Fig. 9)."

Question 3: The reconfigurable capability of shape memory is interesting. I suggest using schematic diagrams to better illustrate this reconfiguration process on the molecular scale, as the mechanism of ion coordination reconfiguration is distinctly different from that of common dynamic covalent bond networks.

Answer 3: Thanks for the Reviewer's suggestion. We have included a schematic diagram to illustrate the shape memory and reconfiguration process of IWH (Supplementary Fig. S40). The solvent-triggered shape reconfiguration of IWH primarily relies on dynamically reversible metal coordination. After programming the hydrogel and immersing it in a 1 M $FeCl_3$ solution, Fe^{3+} can form dynamic coordination bonds with the carboxyl groups on the polymer chains. Through the dynamic nature of

the coordination network, the polymer chains can rearrange and anchor into a new topological structure, thereby transforming from a permanent shape I to a new permanent shape II. This process is based on reversible metal coordination interactions rather than the breaking and reforming of covalent bonds, enabling mild, rapid, and repeatable shape reconfiguration under ambient solvent conditions.

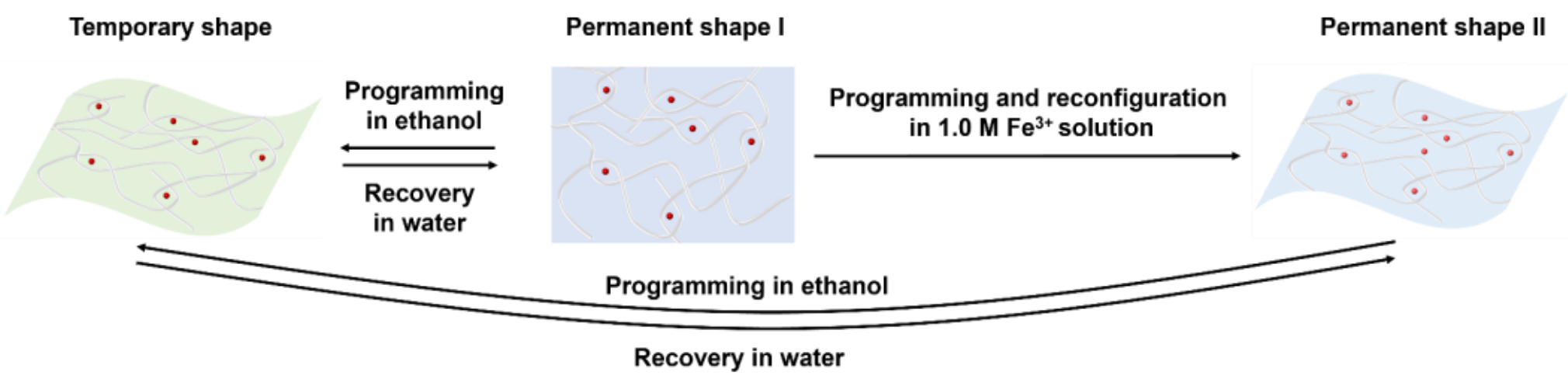

Supplementary Figure 40. Schematic illustration of the shape memory and reconfiguration processes. The IWH can be programmed by solvent stimulation and reconfigured by $FeCl_3$ solution.

We supplement the discussion on Page 20 of the revised manuscript.

"The schematic of shape memory and reconfiguration process is shown in Supplementary Fig. 40. IWH can achieve shape memory through immersion in ethanol and water, and can be immersed in an $FeCl_3$ solution to realize shape reconfiguration."

Question 4: The shape memory process or 4D printing process can be monitored via electrical signals, which is also a significant highlight of this research. The underlying principle lies in the correlation between the deformation degree of the hydrogel and its electrical resistance. Additionally, it seems that the IWH exhibits different electrical resistances or conductivities in various solvents. It is recommended to provide a more detailed explanation in the relevant section regarding the actuation-sensing integration for readers to better understand.

Answer 4: Thanks for the Reviewer's suggestion. In the Supplementary Information,

we provide a more detailed complementary discussion on actuation-sensing integration, elucidating the principle by which IWH enables real-time monitoring of the actuation process through electrical signals. This principle is closely related to macroscopic deformation of the hydrogel, solvent diffusion, chain mobility, ion migration capability, *etc*.

We add the supplementary discussion on Page S8 of the revised supplementary information.

"The sensing principle of the actuation process through electrical signals is as follows. The actuation-sensing integration of IWH arises, on one hand, from the intrinsic correlation between its deformation and electrical signals, and on the other hand, from the dynamic changes of the ion coordination network under solvent action. When IWH undergoes deformation (*e.g.*, stretching, bending, and twisting), its resistance changes according to Pouillet's law. For example, the resistance increases upon stretching and decreases under compression. When IWH is immersed in ethanol, the solvent pressure first causes its resistance to drop. Subsequently, dehydration resulting from solvent exchange hinders chain mobility, thereby fixing the temporary shape, and gradually increases the resistance by suppressing ion migration. Once the solvent exchange is complete, the resistance reaches a stable value. After re-immersing IWH in water, the rehydration caused by solvent exchange promotes chain mobility, restoring the original shape. Meanwhile, the water pressure and the recovery of ion migration capability lead to a decrease in resistance. When the rehydration is complete, the resistance stabilizes again. The changes in electrical signals are closely related to macroscopic deformation, solvent diffusion, chain mobility, ion migration capability, *etc.*, enabling IWH to continuously sense the shape change process, thus achieving actuation-sensing integration."

Question 5: Some data are expressed with slight inconsistencies, such as the mechanical attributes of IWH in the manuscript and supplementary materials. The

author should carefully review the entire paper to unify the data.

Answer 5: Thanks for the Reviewer's suggestion. We have carefully checked all the data in the manuscript and supplementary information, and corrected inconsistent data.

Response to the Reviewer #2

Comment: This study introduces an ionic weaving method to develop multifunctional hydrogels, which possess mechanical robustness, anti-freezing, and anti-swelling properties. Thus, the hydrogel can be used in applications including underwater sensing and intelligent 4D printing with self-sensing capabilities. This work is experimentally comprehensive and demonstrates impressive macroscopic performance. In summary, the topic is innovative, and the results are interesting. The manuscript can be published in Nature Communications after solving the following concerns.

Reply: We are very grateful to the Reviewer for the encouraging comments on our manuscript. We have revised the manuscript and hope to address the issues raised by the Reviewer.

Question 1: The authors indicate that ionic weaving can enhance the stability of polymer networks and dissipate energy through synchronous dissociation. At present, the authors have only investigated the hysteresis area and damping capacity of IWH. To better illustrate the contribution of ionic weaving networks to energy dissipation, it is suggested to supplement the cyclic stress-strain curves of the control group and compare their hysteresis area and damping capacity with those of IWH, thereby supporting the conclusion above.

Answer 1: Thanks for the Reviewer's suggestion. To more clearly illustrate the contribution of the ionic weaving network to energy dissipation, we supplemented the cyclic stress-strain curves of the control group at a strain of 100%. By comparing the hysteresis area and damping capacity, it can be seen that IWH exhibits stronger energy

dissipation capability, highlighting the important role of the ionic weaving network.

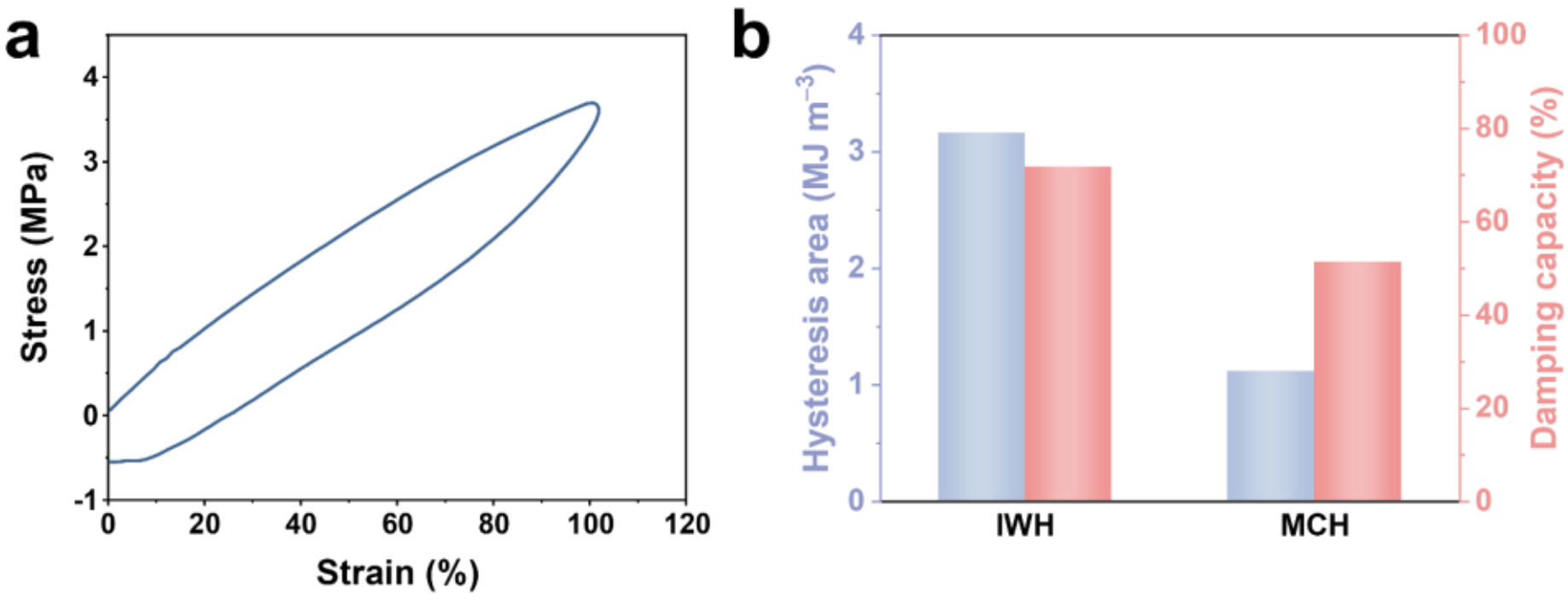

Supplementary Figure 12. Mechanical properties of MCH. a) Cyclic stress-strain curve of MCH at a strain of 100%. b) Dissipated energies and damping capacities of IWH and MCH.

We supplement the discussion on Page 12 of the revised manuscript.

"Compared with the MCH, IWH exhibits dramatically improved dissipated energy and damping capacity (Supplementary Fig. 12)."

Question 2: In the calculation of energy density for the shape memory process, what was the pre-stretching strain of the hydrogel?

Answer 2: Thanks for the Reviewer's question. The pre-stretching strain of the hydrogel is 100%. This value is slightly higher than the strain range of human muscles and is applicable to most actuation scenarios. Therefore, we selected this value as the pre-stretching strain.

We supplement this value on Page S6 of the revised supplementary information.

"The hydrogel was stretched to a pre-strain (100%) and then placed in anhydrous ethanol for shape programming to fix its shape."

Question 3: Why did the water content of the precursor solution need to be reduced to 60% in 3D printing?

Answer 3: Thanks for the Reviewer's question. In 3D printing, the water content of the precursor solution is reduced to 60% primarily to achieve a balance between photopolymerization efficiency and printing resolution. High water content dilutes the monomers and crosslinkers, reducing the photopolymerization rate and conversion degree, which leads to incomplete curing and weak interlayer adhesion, ultimately resulting in poor shape retention. Moreover, high water content increases volumetric shrinkage during curing, causing warping or cracking. On the other hand, low water content makes the precursor solution excessively viscous with poor flowability, and the resulting hydrogel becomes brittle and prone to internal stress. Controlling the water content at around 60% maintains sufficient photoreactivity and curing depth to ensure stable layer-by-layer fabrication, while providing a moderate viscosity that facilitates resin leveling and recoating, thereby enabling the production of high-fidelity 3D hydrogel structures.

Question 4: Why was the initiator in 3D printing replaced from HMPP to LAP?

Answer 4: Thanks for the Reviewer's question. In 3D printing, the replacement of HMPP with LAP as the photoinitiator is mainly owing to the higher water solubility and better compatibility with 405 nm blue light sources. The higher water solubility ensures uniform dispersion of the initiator in the precursor aqueous solution, effectively preventing phase separation or local aggregation. The better matching with the light source provides higher initiation efficiency, enabling fast and efficient polymerization, which in turn helps improve the dimensional accuracy, structural stability, and mechanical uniformity of the printed structures. Therefore, replacing HMPP with LAP effectively enhances the printability, curing performance, and reliability of the hydrogel ink.

Question 5: How long did it take for the programmed IWH to fully recover its original shape after being immersed in water?

Answer 5: Thanks for the Reviewer's question. After being immersed in water, the programmed IWH can fully recover to its original shape within 30–60 seconds, depending on the complexity of the deformation. Meanwhile, its electrical signal can respond synchronously with the deformation process.

Question 6: The reconfigurable shape memory behavior of IWH was set by re-immersion in salt solution. What are the advantages or significance of the solution-based reconfigurable process compared to the thermal reconfigurable process based on other polymers?

Answer 6: Thanks for the Reviewer's question. Thermal-stimulated shape reconfiguration typically utilizes the reversible breaking and reformation of dynamic covalent bonds, which often requires high temperatures. Therefore, the solution-stimulated reconfiguration process in this study exhibits three advantages. First, it enables mild and rapid shape reconfiguration at room temperature, avoiding the adverse effects of high temperatures on the polymer network (such as irreversible thermal oxidative degradation), thereby helping to maintain the mechanical robustness and structural integrity of the material. Second, regulating the shape memory and reconfiguration process of the polymer network through solvent exchange offers simple operation and high controllability, avoiding the issue of uneven heating often encountered in thermal reconfiguration processes. Third, in addition to enabling shape reconfiguration, the dynamic ionic coordination network can simultaneously tune the mechanical and electrical properties of the material, achieving integrated "actuation-sensing" control. These features endow the solution-stimulated reconfiguration process with unique value.

Question 7: Some figures in the manuscript (such as Fig. 4g) have scale bars with

excessively small font sizes. Some photographs do not have scale bars. These need to be revised.

Answer 7: Thanks for the Reviewer's suggestion. We have revised the size of scale bars in Fig. 4g. In addition, we have also added scale bars to all photographs in the manuscript and supplementary information.

Response to the Reviewer #3

Comment: This manuscript proposes a strategy termed ionic weaving hydrogels (IWHs). Specifically, an AM/AA copolymer hydrogel is first subjected to restricted drying (four-edge constrained drying), followed by Fe^{3+} incorporation to generate dynamic coordination crosslinks. The authors claim that this process yields a uniformly distributed three-dimensional topological "weaving" network, which concomitantly enhances strength, modulus, and toughness. The resulting hydrogels are further reported to exhibit minimal swelling, anti-freezing capability, and recoverable performance after dehydration – rehydration cycling, and the work additionally demonstrates 4D printing and self-sensing applications. The manuscript's core claims, specifically the formation of a uniform 3D "ionic weaving" network and its role in coordination-driven toughness and anti-swelling, are not supported by sufficient direct evidence. Critical baselines and controls (e.g., 0% AA, Fe-free, dry-only) are missing, making it impossible to decouple Fe^{3+} coordination effects from processing artifacts such as restricted-drying – induced densification, water-content changes, and anisotropy. Furthermore, the boundary conditions for the restricted-drying process are underdefined, which undermines reproducibility. The extraordinary anti-swelling results lack adequate methodological detail, statistical results, and controls to rule out Fe^{3+} leaching, surface-shell formation, or ionic screening. Mechanical characterization relies heavily on tensile curves without fracture-level testing, and the temperature-dependent coordination dynamics remain unvalidated due to the absence of variable-temperature rheology and in situ spectroscopy. Finally, the biocompatibility evidence

provided is too preliminary to support the claims regarding wearable or biomedical applications. I can reject this manuscript to be published in this journal.

Reply: We greatly appreciate the valuable comments from the reviewer on our manuscript. Your thorough evaluation and insightful suggestions have helped us significantly improve the scientific rigor and completeness of our manuscript. We have conducted a series of additional experiments to address and validate the issues you raised, and we hope you will find them satisfactory. Regarding your concerns about the structure of the uniform 3D ionic weaving network and its relationship with properties, we have further elucidated the uniform distribution of ionic crosslinks through elemental distribution analyses based on XPS and SEM, and we have clarified the correlation between the dynamic network and the outstanding properties using *in situ* rheological experiments and infrared spectroscopy results (Answers 1 and 2). Meanwhile, we have set up three control groups (IWH-0%-0.1 without AA, IWH-20%-0 without Fe^{3+}, and dried P(AAm-*co*-AA)), which rule out processing artifacts and demonstrate that the performance enhancement mainly originates from the metal coordination between Fe^{3+} and polymer chains (Answer 3). Furthermore, we have verified that the anti-swelling property arises from the bulk network effect rather than kinetic suppression (Answer 4). In addition, we have clearly supplemented the detailed experimental parameters for restricted drying, as well as fracture-related and biocompatibility-related tests (Answers 5–7). In summary, we are immensely thankful for the Reviewer's comments and suggestions.

Additionally, we would like to take this opportunity to briefly highlight the uniqueness of our work. We have developed a generalized ionic weaving strategy that enables the construction of a uniformly distributed dynamic metal-coordination network within hydrogels, which is distinctly different from previously reported hydrogel designs. This network effectively restricts the mobility of polymer chains and the ordered arrangement of water molecules, while achieving energy dissipation through synchronous dissociation of metal-coordination interactions, thereby yielding hydrogels with excellent mechanical properties and environmental tolerance. Furthermore, such hydrogels can serve as an intelligent platform for multimodal

sensing and integrated actuation-sensing in various environments, contributing to the development of smart soft robotics. Therefore, from the perspectives of material design, properties, mechanisms, and applications, our ionic weaving hydrogels are both innovative and significant.

Given that we have provided substantial new experimental evidence to address the concerns raised by the reviewer, covering structural characterization, performance investigation, in-depth mechanistic elucidation, and biocompatibility-related tests, we respectfully request that the reviewer re-evaluate our revised manuscript based on our responses. We sincerely hope that this revised version satisfies the reviewers and meets the publication standards of *Nature Communications*. Thank you once again for your valuable time and insightful feedback.

Question 1: The attribution of mechanical performance to dynamic Fe^{3+} – carboxylate coordination is currently under-supported. To distinguish true coordination dynamics from processing artifacts (e.g., drying), "temperature-dependent rheology" (G′/G″/tan δ vs. T in the LVE regime) is essential. Furthermore, claims of reversible dissociation require verification via in situ variable-temperature FTIR rather than ex situ measurements. Correlating spectral changes with rheological relaxation is necessary to rigorously establish the structure – dynamics – property relationship.

Answer 1: Thanks for the Reviewer's suggestion. We verified that the improvement in mechanical properties did not originate from processing artifacts but from metal coordination through more comprehensive experiments. Furthermore, we conducted a detailed and in-depth investigation of the reversible dissociation, thereby establishing rigorous structure-dynamics-property relationships.

First, supplementary experiments show that the improvement in mechanical properties of the hydrogel is not caused by processing artifacts, but originates from effective coordination between metal ions and polymer chains. As shown in Supplementary Fig. 7, the drying step alone causes the hydrogel to dehydrate and form a dried film, which becomes brittle and exhibits poor extensibility, fracturing under

slight stress. Meanwhile, in the absence of ionic coordination (Fe^{3+} concentration of 0 M), the IWH-20%-0 obtained after immersion in solution still exhibits extremely low mechanical properties even after the restricted drying step (fracture strength of 0.8 kPa) (Supplementary Fig. S8).

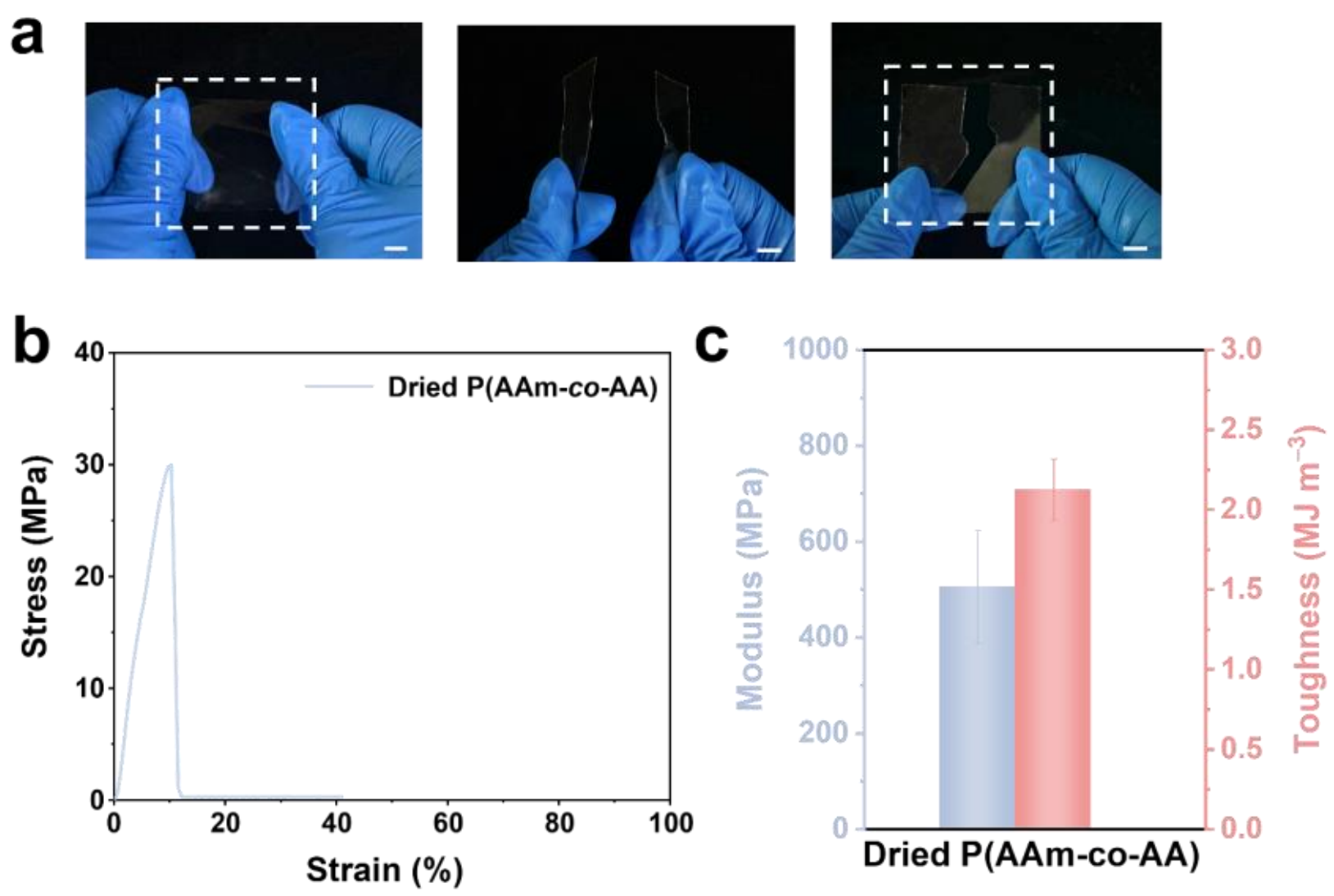

Supplementary Figure 7. Mechanical properties of dried P(AM-*co*-AA). a) Brittle fracture of the dried sample during bending. Scale bar: 1 cm. b) Stress-strain curves of the dried sample. c) Modulus and toughness of the dried sample.

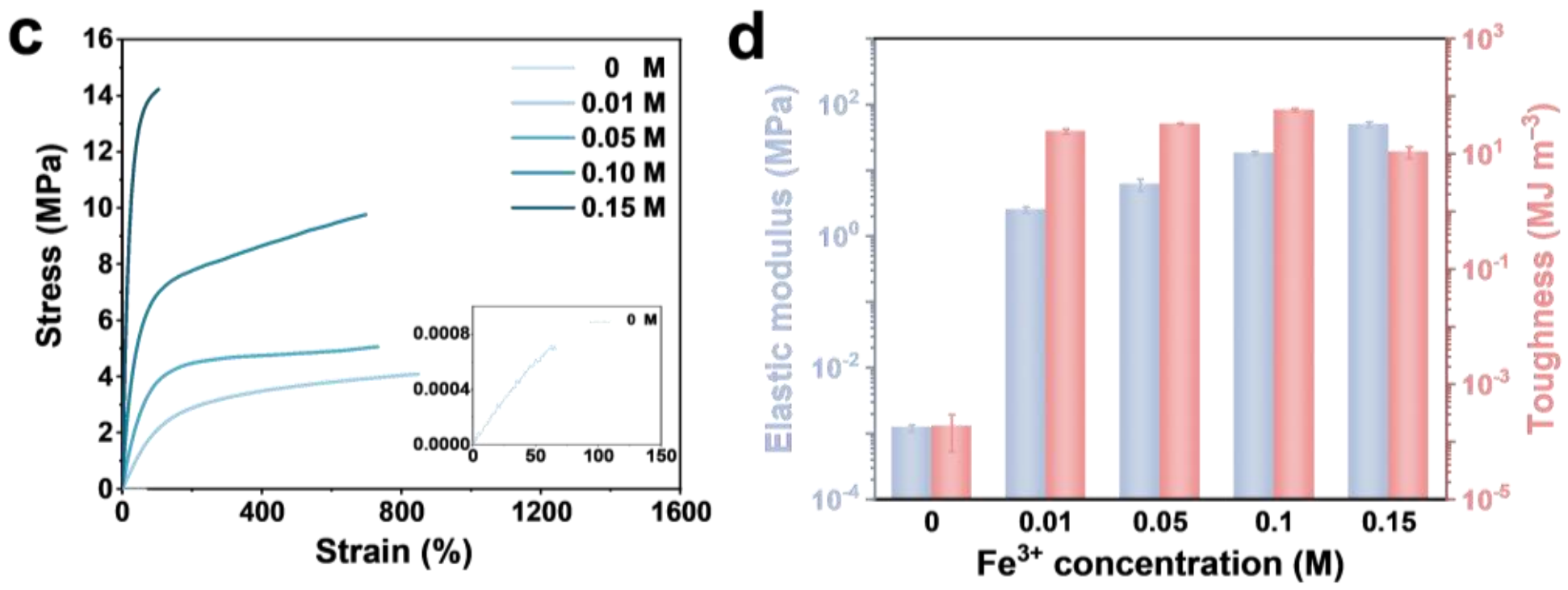

Supplementary Figure 8. c-d) Tensile stress-strain curves of hydrogels with different Fe^{3+} concentrations (c) and the corresponding modulus and toughness (d). The mechanical properties of IWH-20%-0 are very poor owing to the inability to form a stable coordination network.

Furthermore, to our knowledge, the coordination between Fe^{3+} and carboxyl

groups is strong and difficult to dissociate effectively below 100 ºC. Therefore, temperature-dependent experiments such as temperature-variable rheology and temperature-variable infrared spectroscopy, which are used to investigate the dynamic dissociation of metal coordination interactions upon heating, are typically suitable for anhydrous systems such as elastomers. For hydrogel systems, the relatively low upper temperature limit and potential water evaporation make it difficult to clearly observe the decrease in modulus caused by the dynamic dissociation of metal coordination, as well as the temperature-dependent changes of functional groups at the molecular level.

However, the dynamic characteristics can be investigated by step-strain rheology with alternating large and small strains because the dynamic metal coordination network also dissociates under large deformation. As shown in Supplementary Fig. 24, the storage modulus (*G*') of IWH is higher than the loss modulus (*G*'') when scanning at a small strain of 1%. However, when the strain is switched to 100%, *G*'' becomes higher than *G*', indicating the dissociation of metal coordination interactions. Moreover, when the strain is switched back to 1%, both *G*' and *G*'' of IWH rapidly return to their initial values, demonstrating the excellent dynamic reversibility and fast reformation ability of the metal coordination interactions.

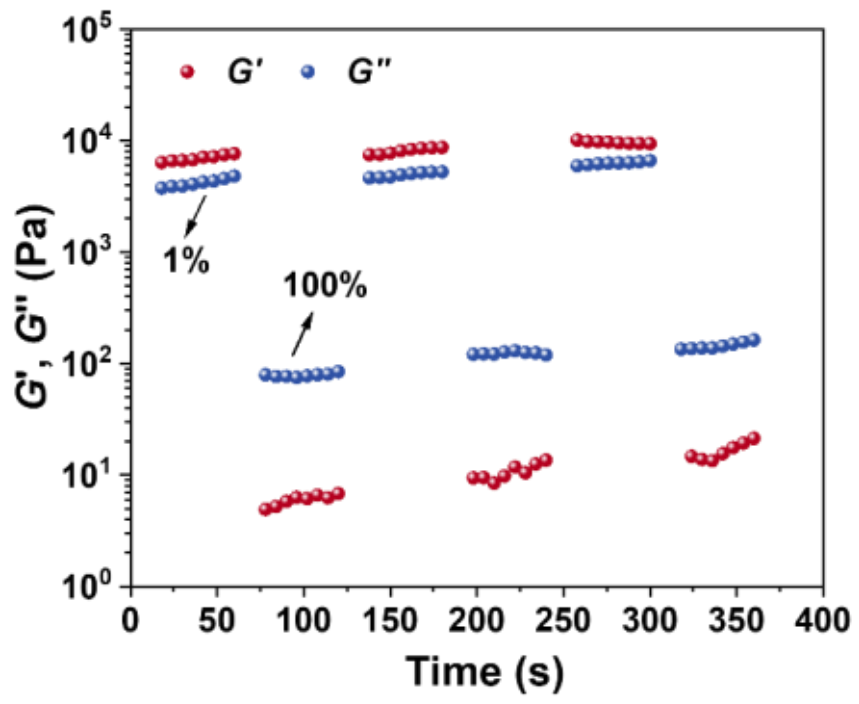

Supplementary Figure 24. Step strain rheological test of IWH with alternating shear strains of 1% and 100%. The IWH possesses a good dynamic nature.

In addition, although the temperature upper limit (within 100 ºC) prevents effective observation of the dynamic dissociation of metal coordination interactions during heating using temperature-variable infrared spectroscopy, the tensile infrared

measurements involved in our mechanistic study are *in situ* tests, rather than *ex situ* tests as the reviewer assumed. *In situ* tensile infrared spectroscopy shows that dynamic dissociation of metal coordination interactions occurs during deformation, thereby effectively dissipating energy to enhance mechanical properties (Fig. 4d-f).

Therefore, through *in situ* spectral changes during deformation and rheological results, we have demonstrated that the ionic coordination network in IWH exhibits excellent dynamic characteristics. When subjected to external forces, it can dissipate energy through the synchronous dissociation of coordination bonds, endowing the hydrogel with high strength, high modulus, and high toughness. Hence, the improvement in mechanical properties primarily originates from the dynamic ionic coordination rather than processing artifacts.

We supplement the discussion on Page 9 of the revised manuscript.

"First, we characterized the mechanical properties of the dried sample to rule out processing artifacts caused by restricted drying, which was brittle and rigid with poor stretchability and fractured immediately upon stretching (Supplementary Fig. 7)."

We supplement the discussion on Page 14 of the revised manuscript.

"Furthermore, the good reversibility of the ionic coordination network in IWH is confirmed through step-strain rheology with alternating small and large strains (Supplementary Fig. 24). At 1% strain, the stable coordination network results in a storage modulus (*G*') higher than the loss modulus (*G*''). When the strain is increased to 100%, the dynamic dissociation of the coordination network leads to the *G*'' exceeding the *G*'. Subsequently, when the strain is switched back to 1%, both the *G*' and *G*'' rapidly return to their initial values owing to the reformation of the coordination network."

Question 2: The claim of a "uniformly distributed 3D topological weaving" network is unsubstantiated. Current characterizations (SAXS, XPS, SEM) capture only average or surface features and fail to rule out diffusion-induced gradients; depth-resolved

measurements are required to verify bulk uniformity. Furthermore, the term "weaving" lacks a clear operational definition. Quantitative metrics are needed to strictly distinguish this specific topology from generic effects like densification or increased crosslink density.

Answer 2: Thanks for the Reviewer's suggestion. We verify the bulk uniformity of IWH using XPS and SEM. First, a central cross-sectional slice was cut from the bulk hydrogel and subjected to XPS analysis. The results show that the surface and the interior of the hydrogel exhibit a similar peak area ratio of Fe^{3+}-COO^{-} (Supplementary Fig. 5 a-b). Furthermore, energy-dispersive spectroscopy was performed on the cross-section of IWH by SEM to investigate the elemental distribution. As shown in Supplementary Fig. 5c, the Fe element is uniformly distributed across the thickness direction, with no obvious aggregation or concentration gradient. In addition, although the previous SAXS results represent averaged information, the absence of scattering from hydrogen-bonded aggregates in the SAXS pattern also reflects the absence of a concentration gradient; otherwise, residual hydrogen bonds would cause the corresponding scattering. Therefore, these results indicate that IWH possesses bulk uniformity rather than a gradient crosslinking structure.

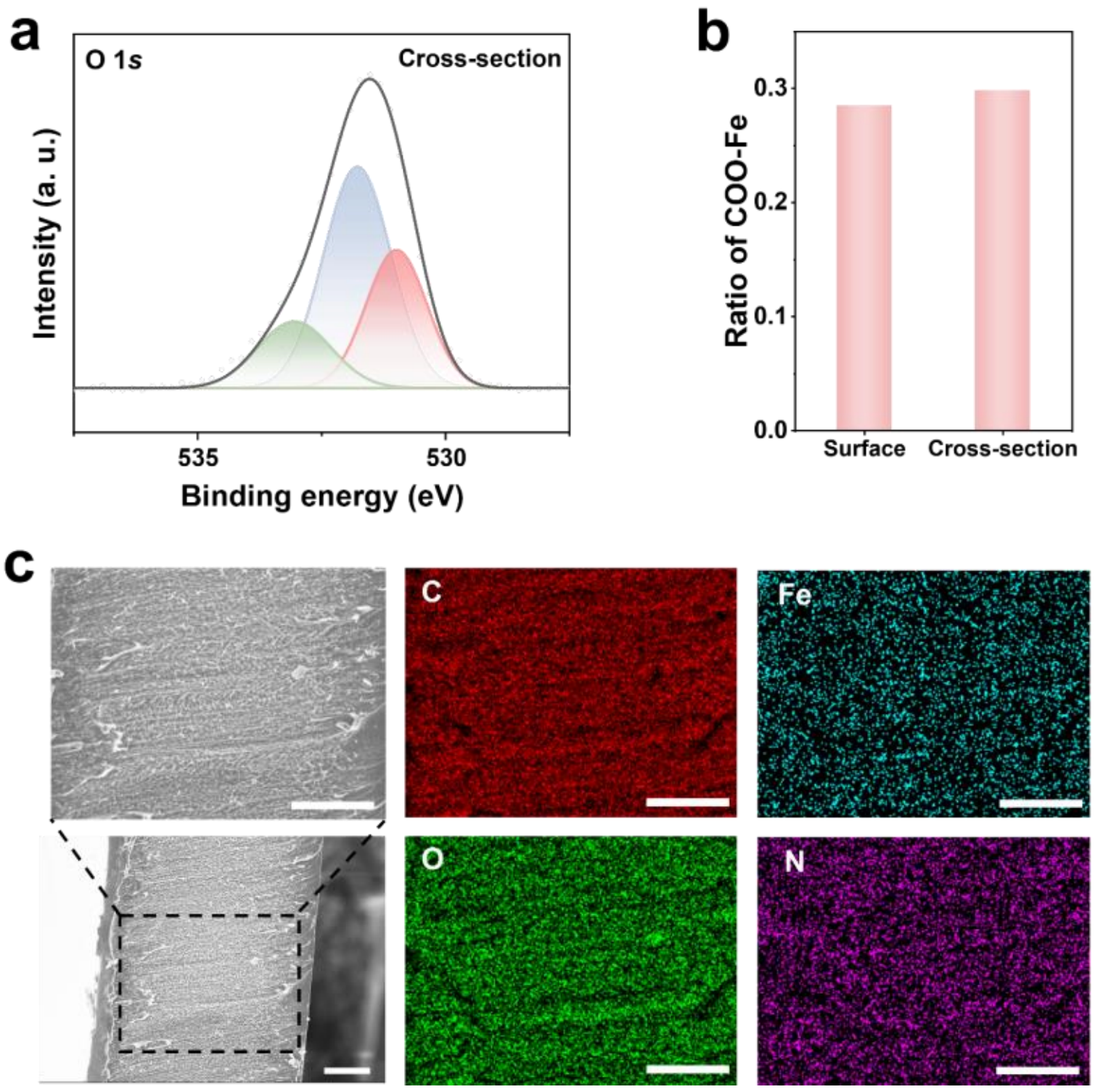

Supplementary Figure 5. Element distribution of the cross-section of IWH. a) O 1*s* XPS spectra of the cross-section of IWH. Green: C-O, blue: C=O, red: COO^{-}–Fe^{3+}. b) Peak area ratios of Fe^{3+}-COO^{-} in the surface and cross-section of IWH. c) Elemental mapping of the cross-section of IWH. Scale bar: 50 μm.

As indicated in the manuscript, "ionic weaving" refers to a strategy for constructing a uniform 3D topological network of dynamic coordination bonds. This is achieved by first forming a closely packed polymer chain template via restricted drying, followed by the introduction of metal ions that form dynamic coordination bonds between the pre-aligned polymer chains. This structure differs significantly from conventional hydrogel networks. For example, densification or an increase in crosslinking density in conventional hydrogels is typically accompanied by the formation of specific aggregates, such as the hydrogen-bonded aggregates within the P(AAm-*co*-AA) hydrogel or the metal polynuclear clusters within the MCH. These aggregates are typically sensitive to SAXS, and once formed, their scattering can be quantitatively characterized by SAXS. Meanwhile, the formation of such aggregates

leads to network heterogeneity, rendering the hydrogel susceptible to local stress concentration or network failure upon deformation. However, the uniform 3D topological network enables effective stress dispersion through the global dissociation of dynamic coordination bonds under deformation. This uniform 3D topological structure achieved via ionic crosslinking resembles a molecularly woven network, in which uniform dynamic crosslinks form a 3D topological network rather than mere densification or an increase in crosslinking density (*Nat. Chem.* **2024**, *16*, 1906–1914; *Nat. Mater.* **2026**, *25*, 107–116). Given that a similar 3D topological structure via ionic crosslinking has not yet been realized in hydrogels, we borrow the concept of molecularly woven networks and name this material an "ionic weaving hydrogel".

We supplement the discussion on Page 9 of the revised manuscript.

"Furthermore, XPS results show that the peak area ratio of COO^{-}−Fe^{3+} is similar on the surface and cross-section of IWH (Supplementary Fig. 5a and 5b). Moreover, energy-dispersive spectroscopy of SEM further indicates that the Fe element is uniformly distributed along the thickness direction of the hydrogel, without aggregation or concentration gradient (Supplementary Fig. 5c)."

Question 3: The experimental design suffers from missing baselines and ambiguous nomenclature. Supplementary Figs. 1 and 6 require AM-only (0% AA) and Fe-free references to isolate chemical contributions. Crucially, a "restricted drying without Fe^{3+}" control is missing, making it impossible to decouple coordination strengthening from processing-induced densification. Figure 1 and terms like "Control" must be revised to explicitly map sample names to their precise composition and processing history (e.g., restricted drying, Fe^{3+}).

Answer 3: Thanks for the Reviewer's suggestion. We supplement two key benchmark control samples, namely IWH-0%-0.10 (the AM-only with 0% AA) and IWH-20%-0 (Fe-free). Both were prepared using the same procedure as IWH, with differences only in the monomer ratio and solution concentration. For the IWH-0%-0.10 sample, the

absence of carboxyl groups prevents Fe^{3+} from crosslinking the polymer chains. For the IWH-20%-0 sample, the absence of Fe^{3+} also prevents crosslinking of the polymer chains. Therefore, as shown in Supplementary Fig. 1, both the IWH-0%-0.10 and IWH-20%-0 samples exhibit severe swelling after immersion owing to the inability to form a stable coordination network. Tensile tests also show that the mechanical properties of these two samples are very poor (Supplementary Fig. 8). Thus, these results indicate that the chemical contribution to the excellent mechanical properties of IWH mainly originates from the coordination between metal ions and polymer chains. In addition, we also include a control sample (dried sample), which was prepared by restricted drying without subsequent immersion in an ionic solution. It can be seen that the dried sample is brittle, lacking extensibility, further demonstrating that the enhancement of mechanical properties does not come from the processing-induced densification (*i.e.*, the drying process) but from the coordination reinforcement (Supplementary Fig. 7).

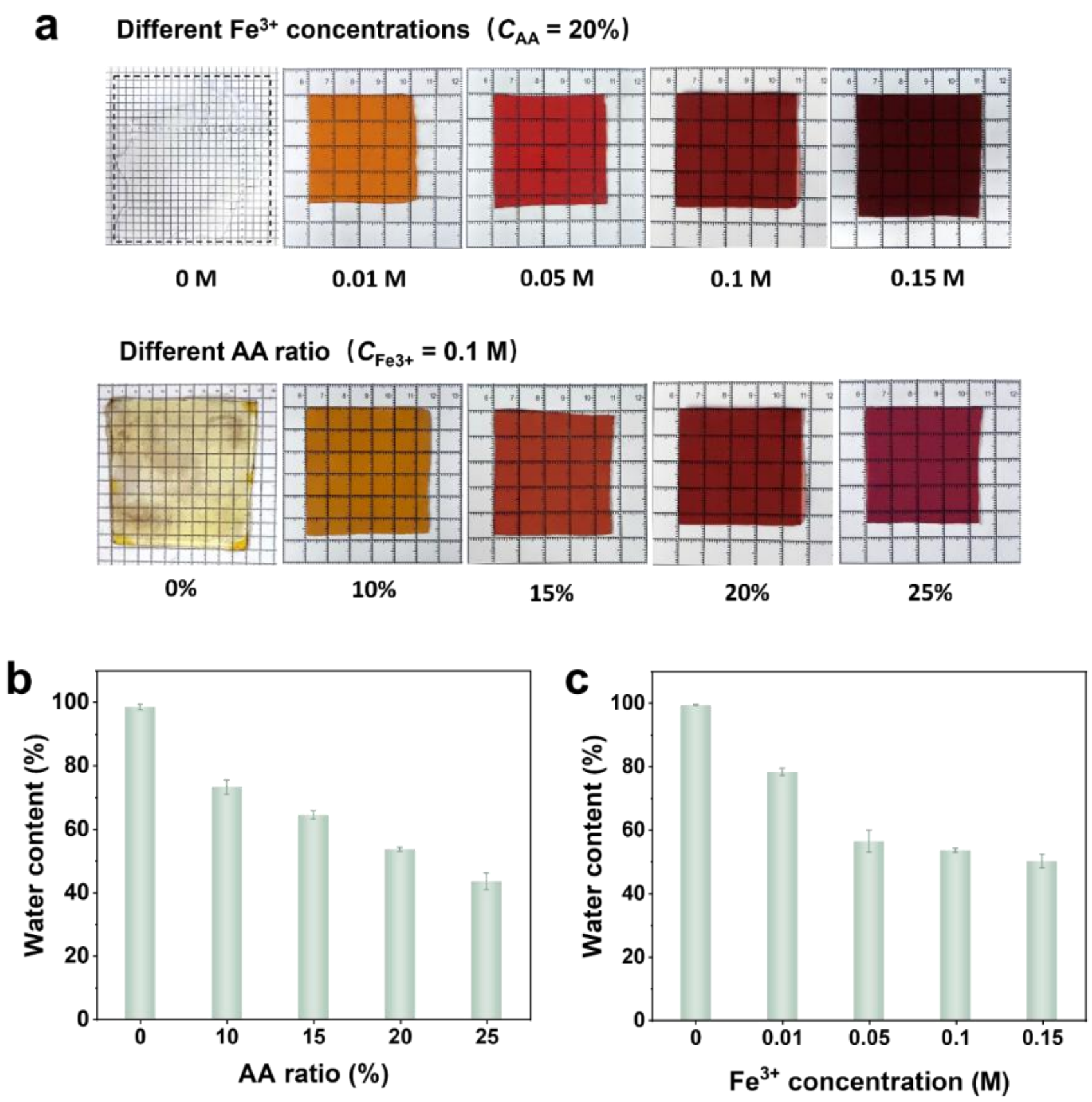

Supplementary Figure 1. Photographs and water contents of different hydrogels. a) Photographs of hydrogel samples with different Fe^{3+} concentrations and AA contents. The side length of the small square is 1 cm. b-c) Water contents of hydrogels with

different AA ratios (b) and Fe^{3+} concentrations (c). Both IWH-0%-0.1 and IWH-20%-0 samples exhibit severe swelling after immersion owing to the inability to form a stable coordination network.

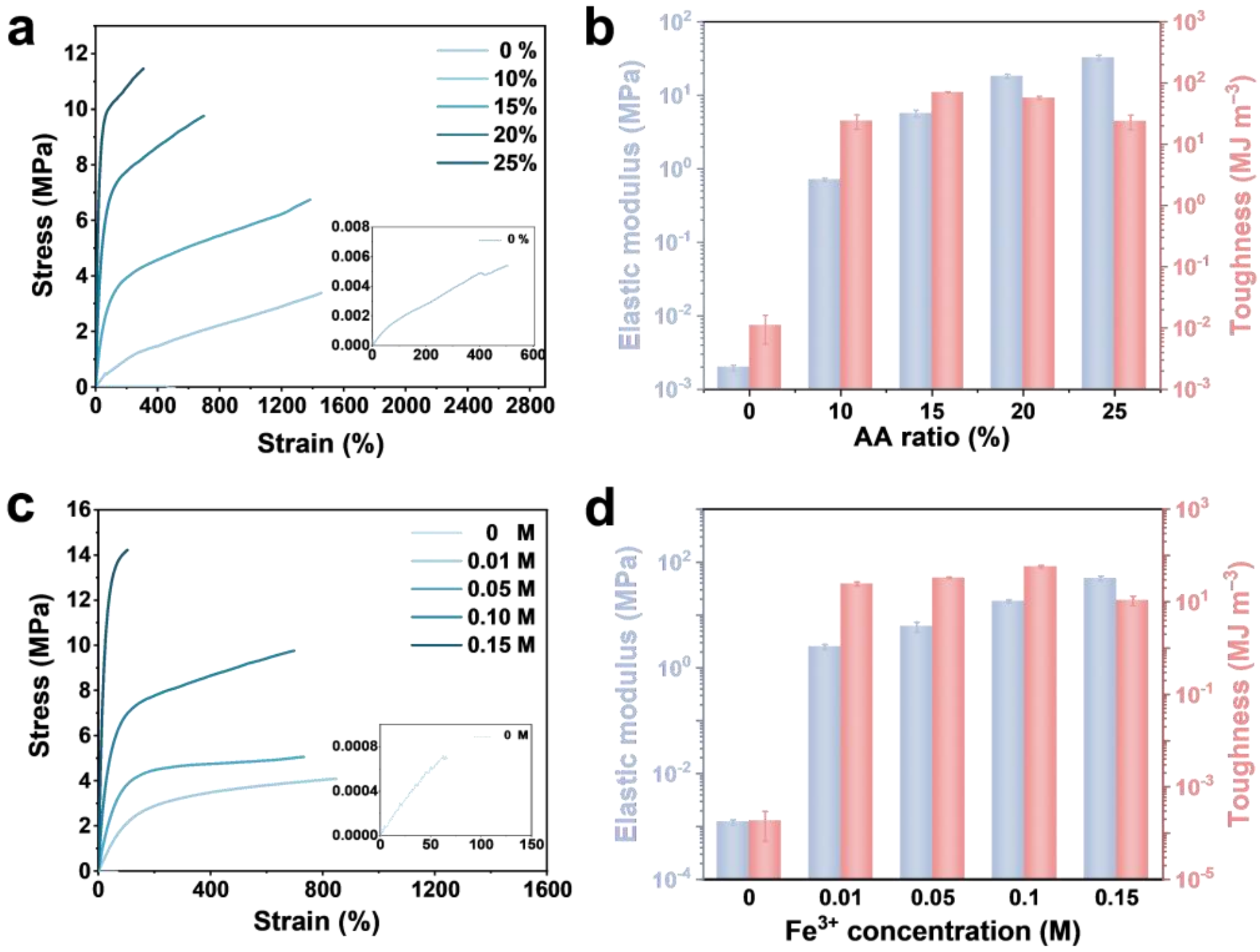

Supplementary Figure 8. Mechanical properties of hydrogels with different AA ratios and Fe^{3+} concentrations. a-b) Tensile stress-strain curves of hydrogels with different AA ratios (a) and the corresponding modulus and toughness (b). c-d) Tensile stress-strain curves of hydrogels with different Fe^{3+} concentrations (c) and the corresponding modulus and toughness (d). The mechanical properties of IWH-0%-0.10 and IWH-20%-0 are very poor owing to the inability to form a stable coordination network.

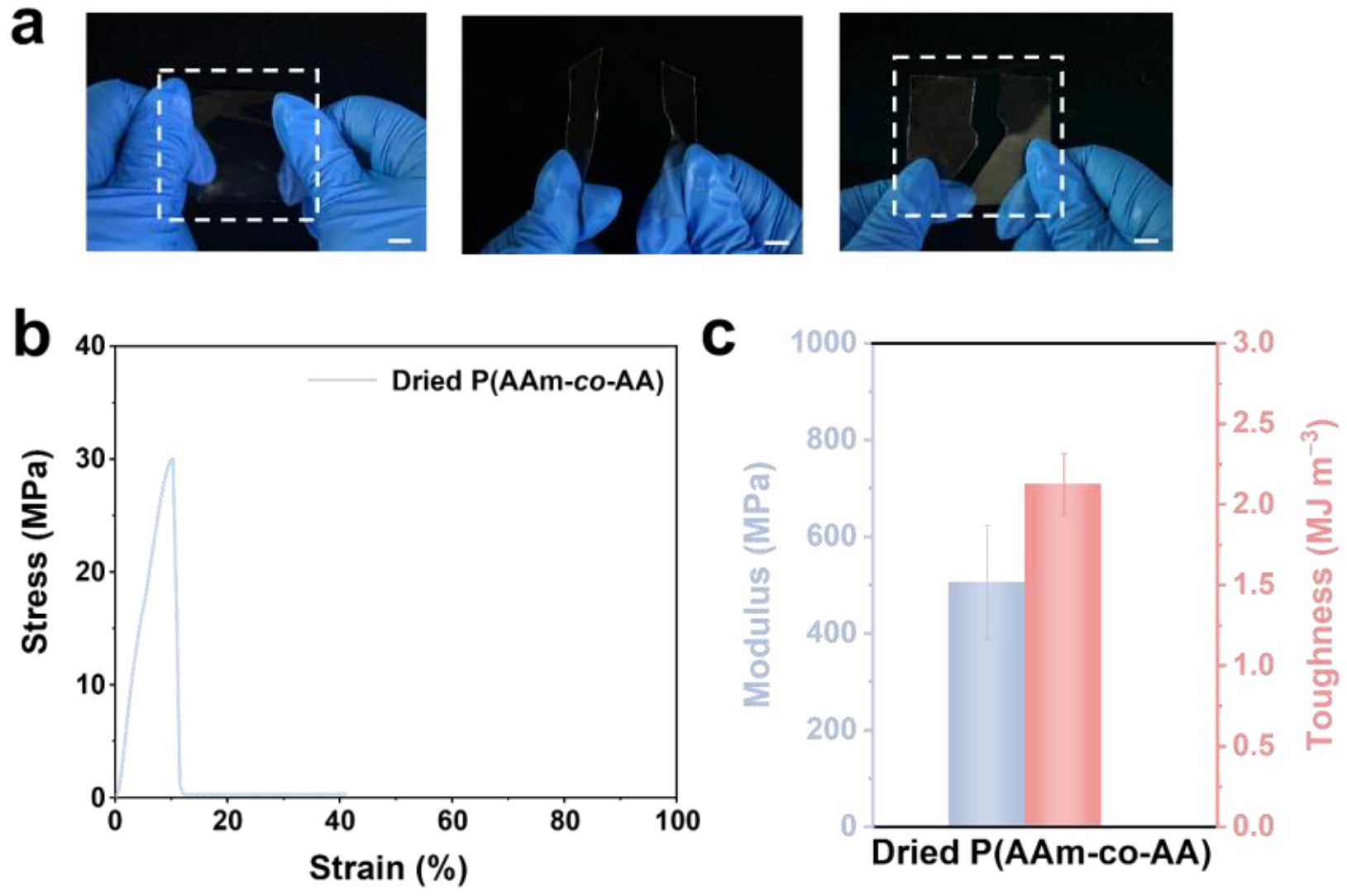

Supplementary Figure 7. Mechanical properties of dried P(AM-*co*-AA). a) Brittle fracture of the dried sample during bending. Scale bar: 1 cm. b) Stress-strain curves of the dried sample. c) Modulus and toughness of the dried sample.

In addition, we have made partial modifications to Fig. 1 to clearly illustrate the preparation process and internal structure of the ionic weaving hydrogel. Moreover, we have modified the terminology and renamed "Control" as "metal coordination hydrogel (MCH)", which is prepared by directly immersing the P(AM-*co*-AA) hydrogel in $FeCl_3$ solution using the conventional soaking method without the restricted drying step. Furthermore, we have provided a more detailed explanation of the abbreviation for the ionic weaving hydrogel, naming it IWH-*m*-*n*, where *m* is the mass ratio of AA to AM, and *n* M is the concentration of the $FeCl_3$ solution used for immersion.

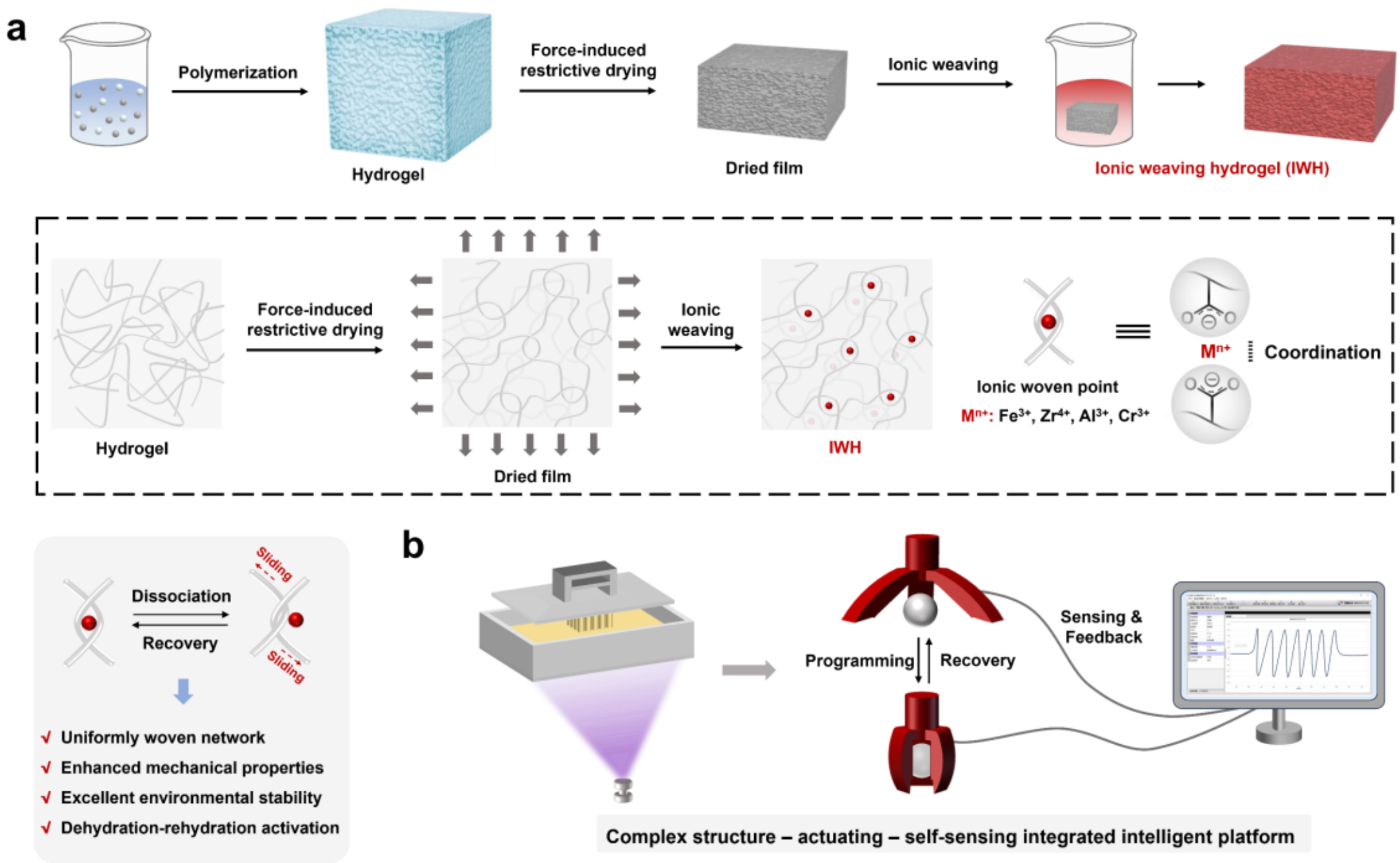

Fig. 1. Design of IWH and integrated intelligent platform. a Schematic illustration of the preparation process and structure of IWH. **b** Schematic illustration of the intelligent platform integrating complex structure, actuation, and sensing functions.

We supplement the discussion on Page 9 of the revised manuscript.

"First, we characterized the mechanical properties of the dried sample to rule out processing artifacts caused by restricted drying, which was brittle and rigid with poor stretchability and fractured immediately upon stretching (Supplementary Fig. 7)."

We supplement the discussion on Page 6 of the revised manuscript.

"A series of hydrogels were prepared and named IWH-*m*-*n*, where *m* is the mass ratio of AA to AM, and *n* M is the concentration of the $FeCl_3$ solution used for immersion."

Question 4: The extraordinary anti-swelling claim (~0.5% vs. ~7225%) lacks rigorous validation. Key experimental parameters (sample geometry, surface-to-volume ratio, pH/ionic strength) are undefined. Crucially, the current data fails to distinguish a true bulk network from kinetic suppression via a surface "shell" or Fe^{3+} leaching. The

authors must provide thickness-dependent swelling kinetics, quantified Fe leaching (ICP-MS), and cross-sectional mapping to confirm bulk uniformity. Additionally, statistical data (n>=3, mean SD) and visual documentation are required to ensure reproducibility.

Answer 4: Thanks for the Reviewer's suggestion. The key experimental parameters in the anti-swelling experiment have been supplemented. The sample size is 1 cm × 1 cm × 1 mm, with a surface-area-to-volume ratio of 24 cm^{-1}. The swelling experiment was carried out in neutral deionized water, whose ionic strength is negligible.

Furthermore, to confirm that the anti-swelling property originates from the bulk network rather than kinetic suppression, we performed swelling tests on samples with three different thicknesses (0.5 mm, 1.0 mm, and 1.5 mm), accompanied by statistical data and photographic documentation. The results show that the swelling ratio remains at a similar level (~0.5%) regardless of sample thickness, and the photographs reveal that both the surface area and thickness of the hydrogel exhibit almost no change after immersion (Supplementary Fig. 18). Additionally, to rule out the effect of Fe^{3+} leaching, we quantitatively determined the Fe^{3+} concentration in the solution after 3 and 6 days of immersion using ICP-MS. The results show that the Fe^{3+} concentration in the solution after immersing IWH is approximately ~0.5 mg L^{-1} (Supplementary Fig. 20). The calculated leaching amount of Fe^{3+} is less than 3%, indicating that the coordination network is stable during the swelling process, with very little Fe^{3+} leaching. Meanwhile, we performed elemental analysis on the cross-section of the hydrogel using SEM and XPS to demonstrate bulk homogeneity and exclude the influence of a surface shell effect. XPS results also show that the surface and interior of the hydrogel exhibit a similar peak area ratio of Fe^{3+}-COO^{-} (Supplementary Fig. 5a-b). SEM results show that the Fe element is uniformly distributed along the thickness direction, without obvious aggregation or concentration gradient, and no surface shell is present (Supplementary Fig. 5c). Therefore, the excellent anti-swelling capability of IWH originates from its bulk network characteristics, rather than from the kinetic inhibition effect of a surface shell or Fe^{3+} leaching.

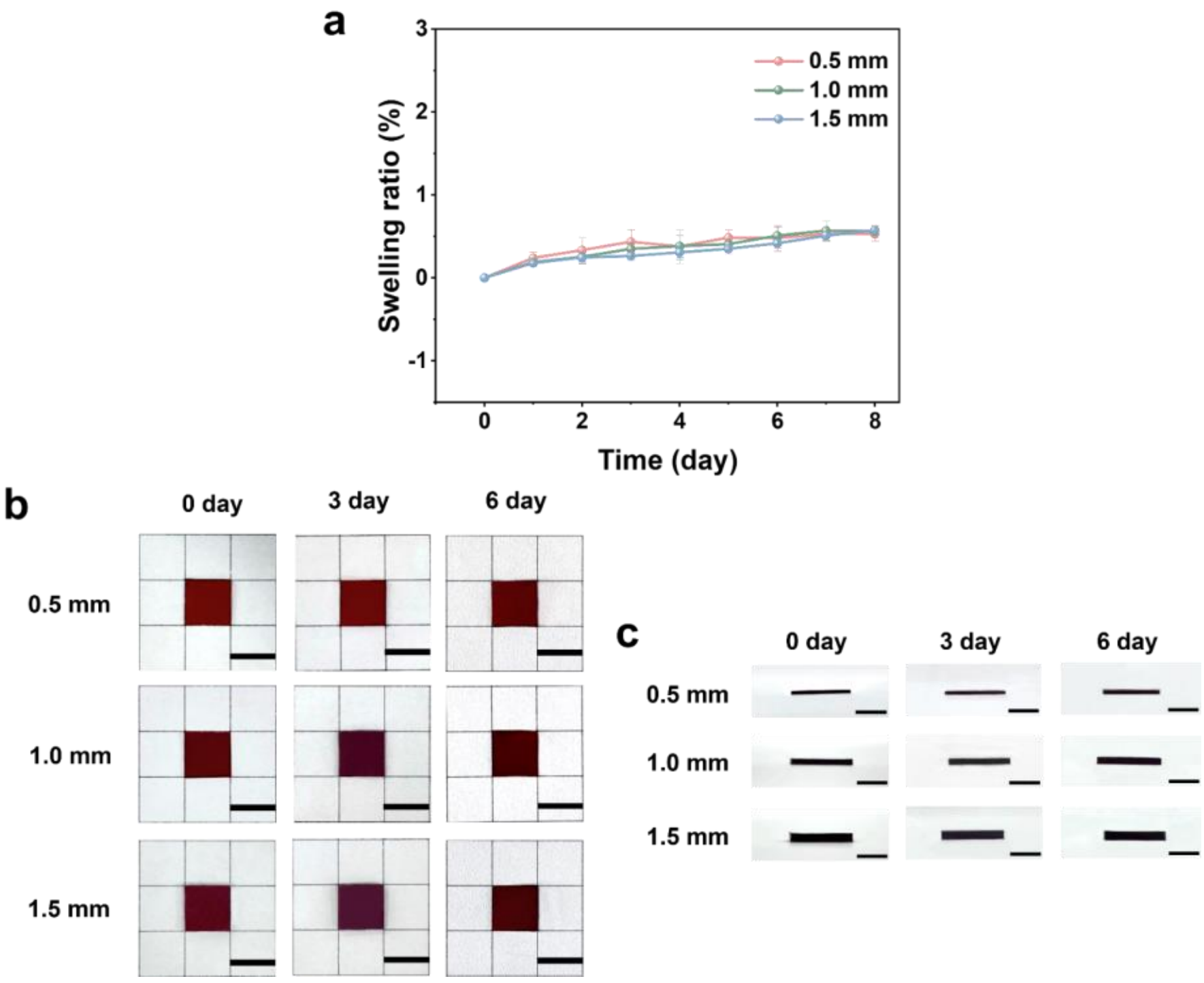

Supplementary Figure 18. Anti-swelling property of IWH. a) Swelling curves of IWHs with different thicknesses over 0–8 days. Scale bar: 1 cm. b-c) Photographs showing the changes in area (b) and thickness (c) of IWHs with different thicknesses at 0, 3, and 6 days. Scale bar: 5 mm.

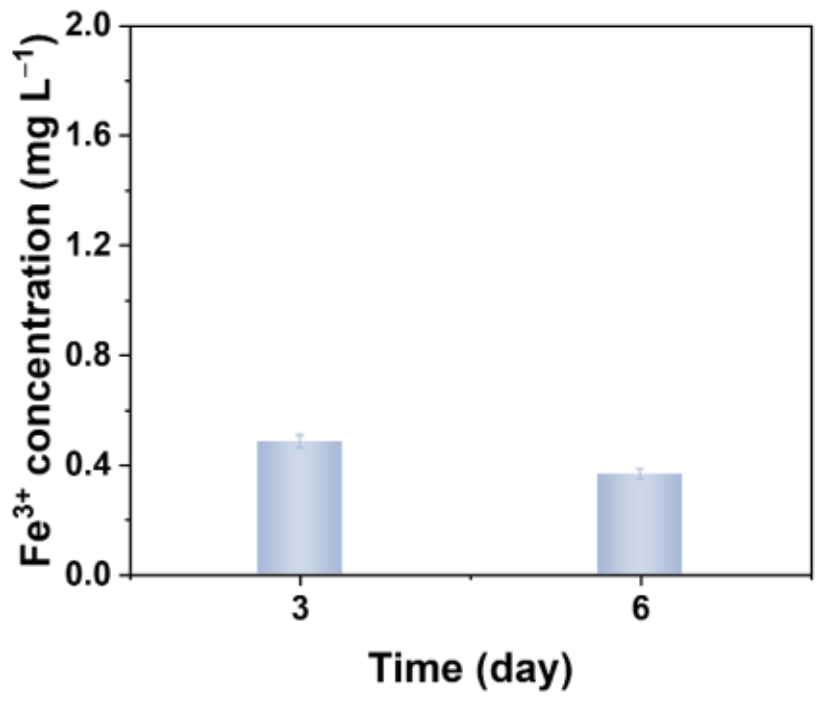

Supplementary Figure 20. Fe^{3+} concentration of the solution after immersing IWH in deionized water for 3 and 6 days. The volume of deionized water used during the swelling test is 100 mL, and the size of IWH is 1 cm × 1 cm × 1 mm. It is estimated that the leaching amount of Fe^{3+} is less than 3%.

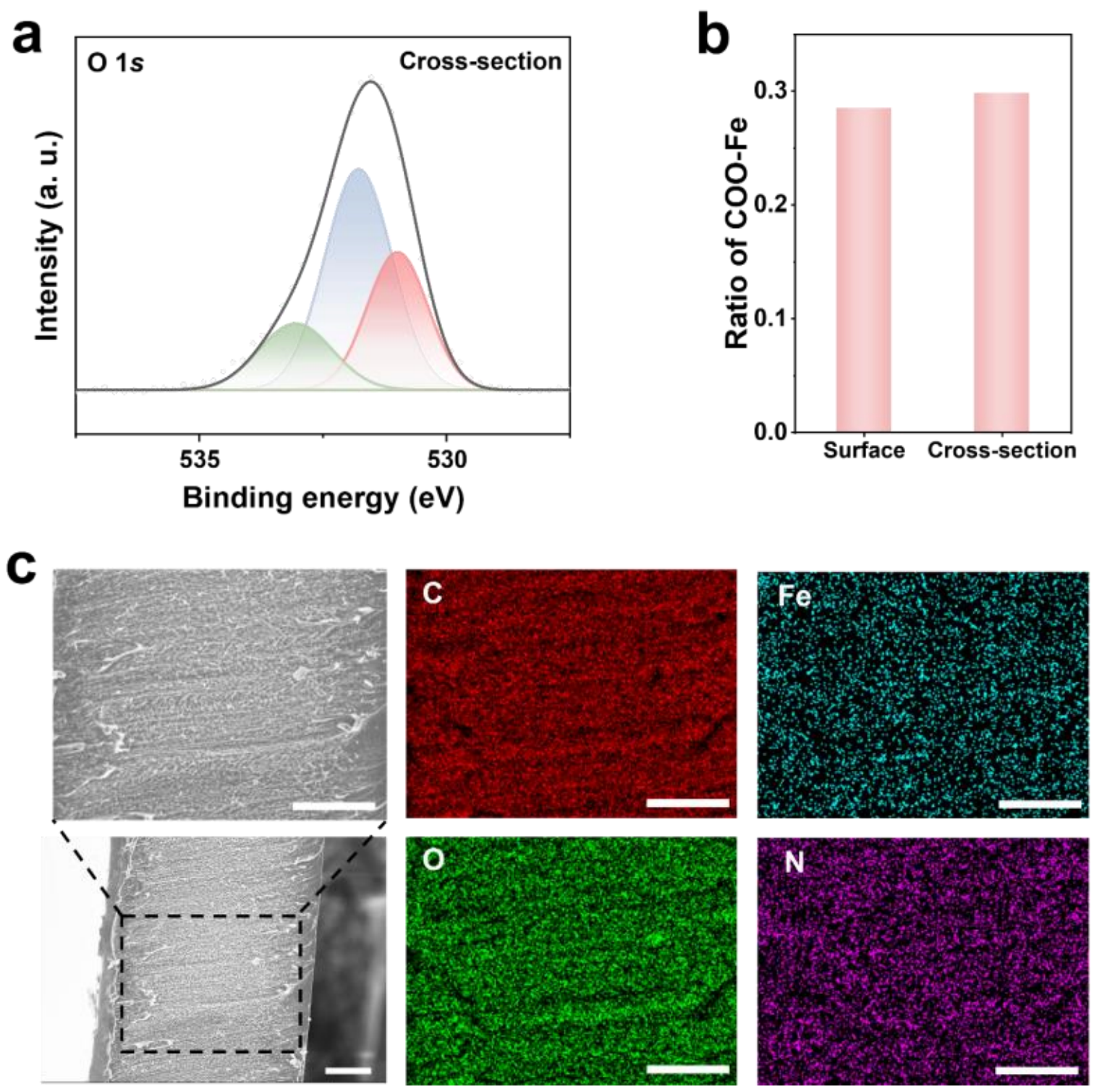

Supplementary Figure 5. Element distribution of the cross-section of IWH. a) O 1*s* XPS spectra of the cross-section of IWH. Green: C-O, blue: C=O, red: COO^{-}–Fe^{3+}. b) Peak area ratios of Fe^{3+}-COO^{-} in the surface and cross-section of IWH. c) Elemental mapping of the cross-section of IWH. Scale bar: 50 μm.

We supplement the discussions on Page 13 of the revised manuscript.

"Changing the thickness of IWH does not affect its anti-swelling capability (Supplementary Fig. 18)."

"Moreover, the leaching amount of Fe^{3+} during the swelling test is extremely low, confirming the high stability of the coordination network (Supplementary Fig. 20)."

We supplement the discussion on Page 11 of the revised manuscript.

"Furthermore, XPS results show that the peak area ratio of COO^{-}–Fe^{3+} is similar on the surface and cross-section of IWH (Supplementary Fig. 5a and 5b). Moreover, energy-dispersive spectroscopy of SEM further indicates that the Fe element is uniformly distributed along the thickness direction of the hydrogel, without aggregation or concentration gradient (Supplementary Fig. 5c)."

We supplement the experimental details on Page S5 of the revised supplementary information.

"**Swelling Test:** The samples were cut into square specimens of 1 cm × 1 cm with thicknesses of 0.5 mm, 1.0 mm, and 1.5 mm. The specimens with different thicknesses were immersed in deionized water at room temperature, and the sample weights were recorded periodically. All tests were performed in triplicate for each thickness, and the data are presented as mean ± standard deviation (SD) to ensure the reliability and reproducibility of the results. The swelling ratio of the specimens was calculated using the following equation:

$$W = \frac{m_t - m_0}{m_0} \times 100\%$$

where W is the swelling ratio, m_0 is the initial weight of the sample, and m_t is the real-time weight of the sample after immersion."

Question 5: The current biocompatibility assessment (24 h MTT, single cell line) is insufficient to support wearable/biomedical applications. To validate safety, time-dependent Fe^{3+} leaching must be quantified via ICP-OES/MS in relevant media (e.g., simulated sweat). Additionally, cytotoxicity testing requires expansion to skin-relevant cells (fibroblasts/keratinocytes) over multiple time points (24–72 h). Without these data, biomedical claims must be significantly moderated to "preliminary screening."

Answer 5: Thanks for the Reviewer's suggestion. To further verify the biocompatibility of this hydrogel, we used ICP-MS to investigate whether Fe^{3+} leaching occurs from the hydrogel in simulated sweat. The results show that the Fe^{3+} concentration in the simulated sweat is approximately 0.2 mg/L after soaking the IWH for 3 and 6 days (Supplementary Fig. 33). The calculated Fe^{3+} leaching amount is less than 1.5%, indicating that the Fe^{3+} release is low and within a safe range.

We also extended the cytotoxicity tests to include skin fibroblasts and skin keratinocytes, with incubation times of 24 h, 48 h, and 72 h. The results demonstrate

that the IWH has good biocompatibility, with high cell viability for all cell types (Supplementary Fig. 32). These results provide a preliminary safety basis for its application in wearable devices.

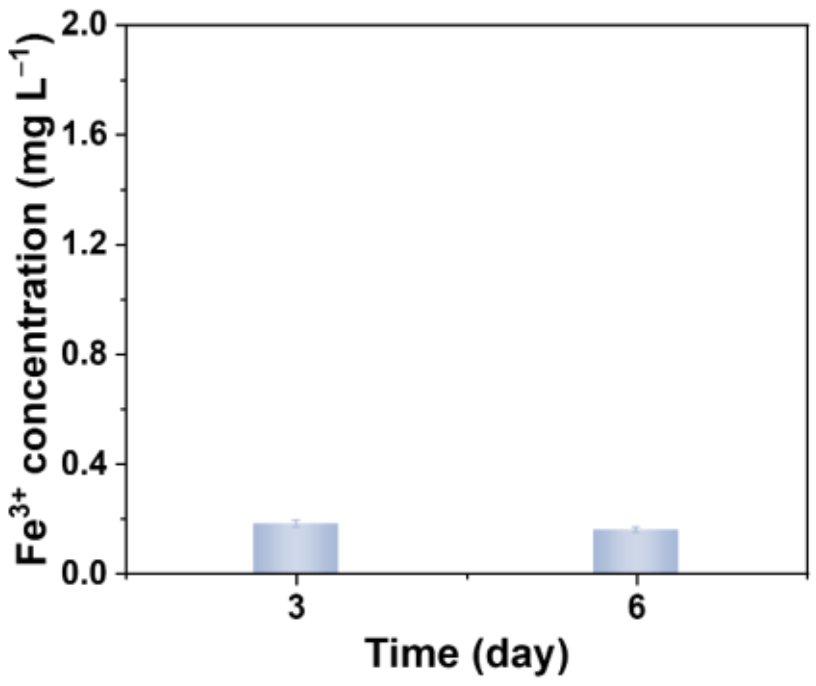

Supplementary Figure 33. Fe^{3+} concentration of the solution after immersing IWH in the simulated sweat for 3 and 6 days. The volume of the simulated sweat used during the swelling test is 100 mL, and the size of IWH is 1 cm × 1 cm × 1 mm. It is estimated that the leaching amount of Fe^{3+} is less than 1.5%.

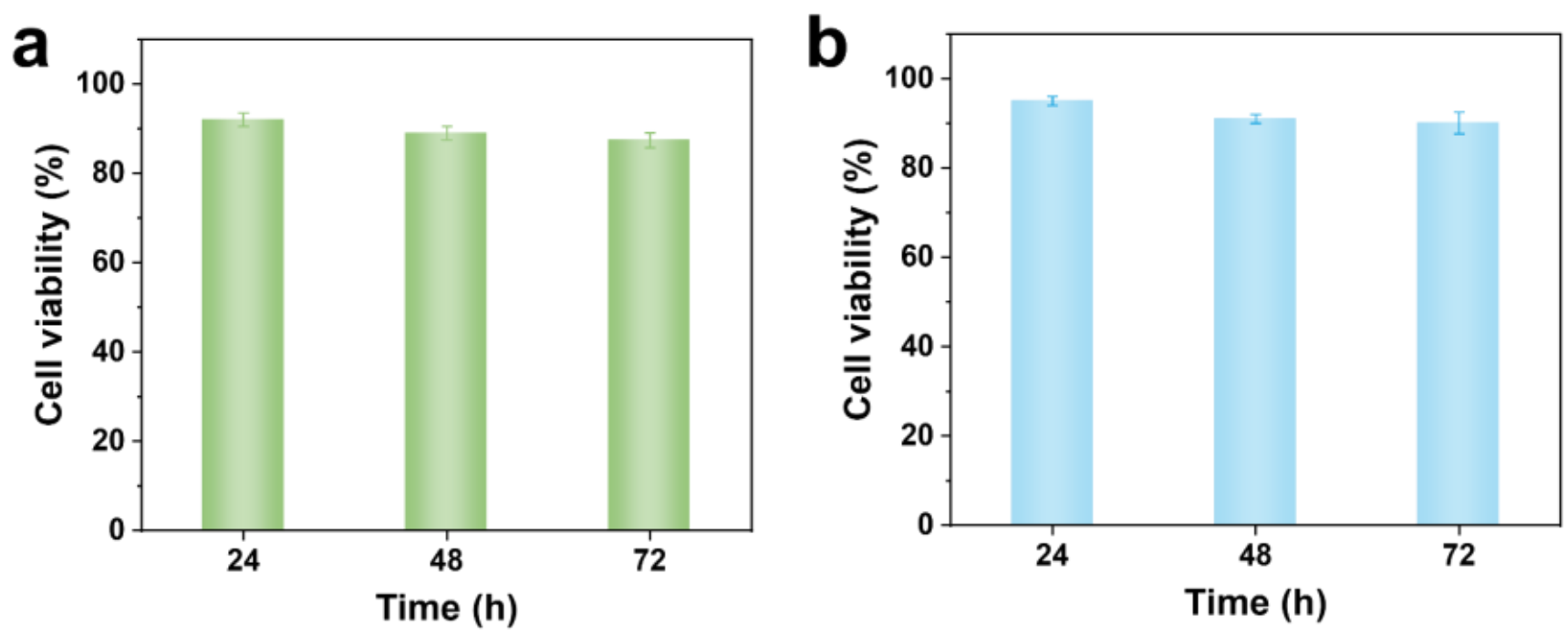

Supplementary Figure 32. Cytotoxicity test of IWH. a) Cell viability of skin fibroblasts incubated with IWH for different times. b) Cell viability of skin keratinocytes incubated with IWH for different times.

We supplement the discussion on Page 18 of the revised manuscript.

"After immersion in simulated sweat, the leaching amount of Fe^{3+} is extremely low, indicating that the release of Fe^{3+} is within a safe range (Supplementary Fig. 33)."

Question 6: The "restricted drying" process, crucial for the "ionic weaving" mechanism, lacks sufficient detail to ensure reproducibility. The manuscript fails to define key boundary conditions, specifically: ambient parameters (temperature, humidity, airflow), specimen geometry/constraints (dimensions, clamping, applied tension), and the drying endpoint criteria. Given the sensitivity of this method, video documentation of the setup and long-term time-lapse observations are required to capture the full drying evolution and distinguish robust properties from environment-dependent processing artifacts.

Answer 6: Thanks for the Reviewer's suggestion. During the restricted drying process, the temperature, humidity, and airflow were controlled at 25 °C, 60% RH, and natural convection, respectively. The sample dimensions were 5 cm × 5 cm in length and width. The sample was clamped onto a glass slide without pre-applied tension. The fixed boundaries generated internal forces during drying to achieve pre-alignment of the polymer chains. The endpoint of drying was defined as the sample reaching a constant weight state, *i.e.*, no significant mass change between two consecutive weighings.

To capture the complete drying evolution process, the hydrogel was dyed red for easy observation. In addition, owing to the long drying time, the evolution was recorded by taking photographs at different time points (0 h, 8 h, 16 h, and 24 h). Over time, the sample gradually dried, resulting in a progressive darkening of its color (Supplementary Fig. 2b). However, because the boundaries were fixed, the hydrogel underwent only a decrease in thickness during drying, while its length and width remained unchanged.

In addition, we also include a control sample (dried sample), which was prepared by restricted drying without subsequent immersion in an ionic solution. It can be seen that the dried sample is brittle, lacking extensibility, further demonstrating that the enhancement of mechanical properties does not come from environment-dependent processing artifacts (*i.e.*, the drying process) but from coordination reinforcement (Supplementary Fig. 7).

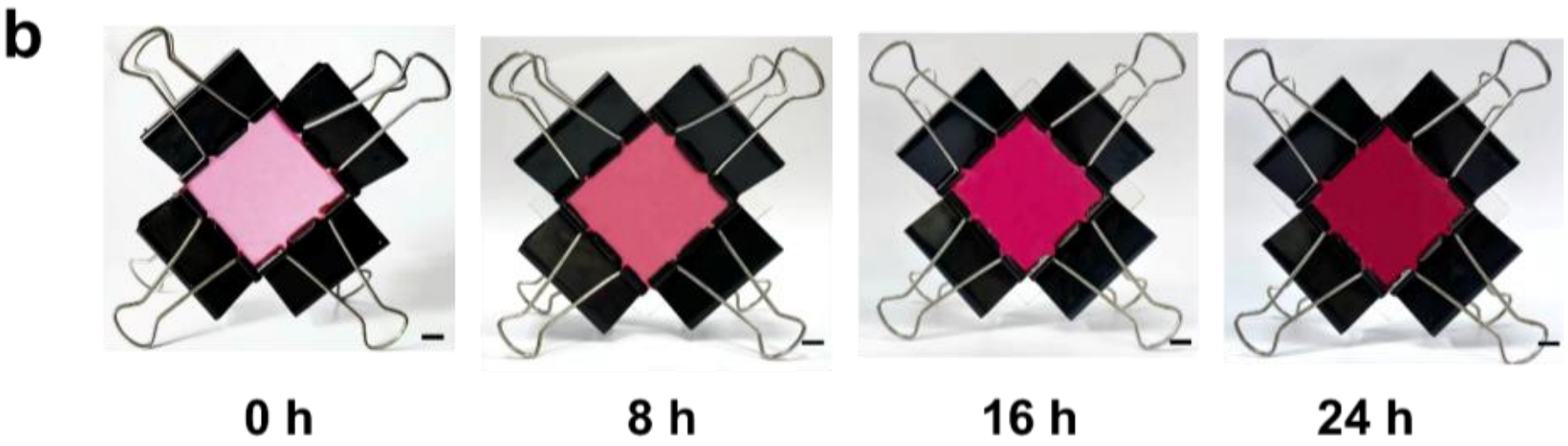

Supplementary Figure 2. b) Photographs showing the morphological evolution of the P(AM-*co*-AA) hydrogel during restricted drying at 0, 8, 16, and 24 h. Scale bar: 1 cm. (The P(AM-*co*-AA) hydrogel was dyed red for clarity of observation).

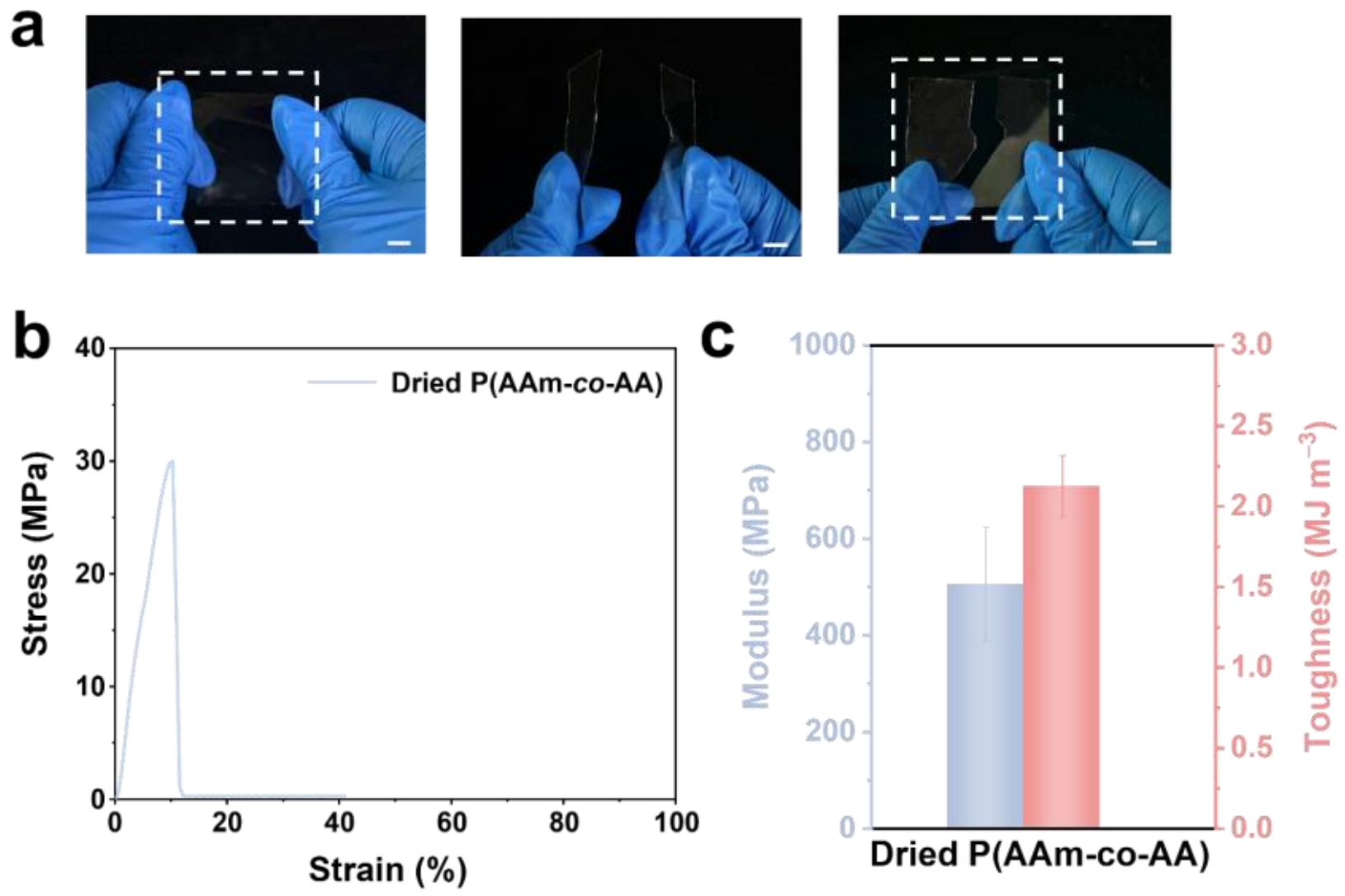

Supplementary Figure 7. Mechanical properties of dried P(AM-*co*-AA). a) Brittle fracture of the dried sample during bending. Scale bar: 1 cm. b) Stress-strain curves of the dried sample. c) Modulus and toughness of the dried sample.

We supplement the discussion on Page S3 of the revised supplementary information.

"The sample dimensions were 5 cm × 5 cm in length and width. Its four sides were secured with clamps onto a glass slide without pre-applied tension, and it was left to dry naturally in an indoor environment (25 ºC, 60% RH, natural convection) for one day. The fixed boundaries generated internal forces during drying to achieve the pre-alignment of polymer chains. The endpoint of drying was defined as the sample reaching a constant weight state, *i.e.*, no significant mass change between two

consecutive weighings.”

We supplement the discussion on Page 11 of the revised manuscript.

“First, we characterized the mechanical properties of the dried sample to rule out processing artifacts caused by restricted drying, which was brittle and rigid with poor stretchability and fractured immediately upon stretching (Supplementary Fig. 7).”

Question 7: The reliance on tensile toughness (area under the curve) is inadequate, as this metric is often flaw-insensitive. To validate the "3D topological weaving" mechanism, fracture-level characterization (e.g., notch/tear tests, fracture energy) is required to quantify actual crack resistance and defect tolerance.

Answer 7: Thanks for the Reviewer’s suggestion. As pointed out by the reviewer, tensile toughness is insensitive to defects and therefore cannot reflect the crack resistance of a hydrogel. Thus, in the original manuscript, we have employed the single-edge crack test to measure the fracture energy of IWH and the control samples in the presence of a notch (Supplementary Fig. 17). IWH exhibits a higher fracture energy compared to MCH and P(AAm-*co*-AA). Furthermore, we calculated the fractocohesive length for the different samples based on these data. When the defect size is smaller than this value, the hydrogel is insensitive to defects. The fractocohesive length of IWH (2.2 mm) is higher than that of MCH (0.47 mm), further demonstrating that the ionic weaving strategy can enhance the defect tolerance of the metal-coordination network.

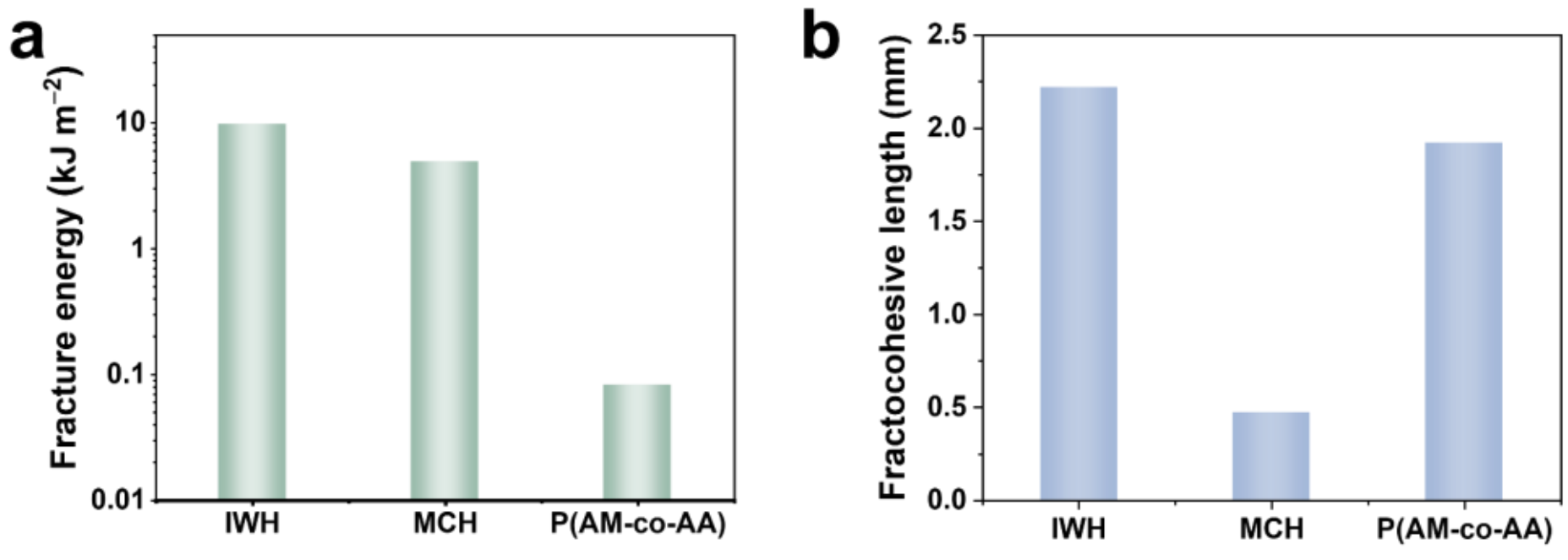

Supplementary Figure 17. Crack resistance of different hydrogels. a) Fracture

energies of IWH, MCH, and P(AM-*co*-AA). b) Fractocohesive lengths of IWH, MCH, and P(AM-*co*-AA).

We revise the discussion on Page 12 of the revised manuscript.

"Meanwhile, the fracture energy and fractocohesive length of IWH (9.8 kJ m^{-2}, 2.2 mm) are also higher than those of the MCH sample (4.9 kJ m^{-2}, 0.47 mm) and P(AM-*co*-AA) hydrogel (0.08 kJ m^{-2}, 1.9 mm) (Supplementary Fig. 17)."

Response to Reviewers

We are appreciative of the feedback from our peer reviewers. Their constructive comments have significantly contributed to enhancing our manuscript. In response to their concerns, we have expanded our research with additional analyses and discussions. These improvements are thoroughly integrated into the revised manuscript.

To facilitate a clear understanding of our revisions, we have organized our responses as follows:

Reviewer Comments and Our Responses: Each comment from the Reviewers (presented in black text) is followed by our corresponding response (in blue text).

Highlighted Text in the Revised Manuscript: Text modifications in the revised manuscript are red in the response letter.

Response to Reviewer #1

Comment: The authors replied to my comments and questions properly, which satisfied me sufficiently. I think that it can be published in Nature Communications now.

Reply: We are very grateful to the Reviewer for the encouraging comments on our manuscript.

Response to Reviewer #2

Comment: The authors have adequately revised their manuscript. I have no further questions.

Reply: We are very grateful to the Reviewer for the encouraging comments on our manuscript.

Response to Reviewer #3

Comment: The revised manuscript has been substantially improved. The added Fe-free, AA-free, and dry-only controls, together with the clarified restricted-drying conditions,

strengthen the mechanistic discussion and reproducibility. The additional fracture-related tests, Fe3+ leaching analysis, and cytotoxicity experiments also improve the overall quality of the work. However, several issues still require revision.

Reply: We are very grateful to the Reviewer for the encouraging comments on our manuscript. We have revised the manuscript and hope to address the issues raised by the Reviewer.

Question 1: First, the temperature-dependent coordination dynamics remain insufficiently validated. The step-strain rheology and in situ tensile FTIR support deformation-induced dissociation/reformation of Fe3+ − carboxylate coordination, but they do not fully replace temperature-dependent rheology or variable-temperature spectroscopy. The authors should clearly acknowledge this limitation and avoid implying that temperature-dependent coordination dynamics have been directly verified.

Answer 1: Thanks for the reviewer's valuable suggestion. We agree that the dynamic nature of the Fe^{3+}–carboxylate coordination network is primarily inferred from deformation-related experiments such as step-strain rheology and in situ tensile FTIR spectroscopy. These experiments confirm the reversible dissociation and reformation of coordination interactions under mechanical deformation. However, these results do not directly verify temperature-dependent coordination dynamics. We have carefully moderated the relevant statements and clarified this limitation in the revised manuscript. Specifically, we now state that the dynamic nature of the coordination network in this work is inferred from mechanical deformation experiments rather than directly demonstrated through variable-temperature experiments. We also avoid implying that temperature-responsive coordination dynamics have been experimentally verified. We appreciate the reviewer's suggestion and believe the revised wording more accurately reflects the scope of the experimental evidence.

We supplement the discussion on Page 17 of the revised manuscript.

"Furthermore, it should be noted that although we have confirmed the dynamic nature

of the coordination network through deformation-induced dissociation and reformation, temperature-dependent coordination dynamics were not directly investigated in this study."

Question 2: Second, the Introduction should refine the definition of the literature gap. The current discussion attributes hydrogel environmental instability mainly to uneven interchain interactions and heterogeneous phase-separated structures. This explanation is somewhat oversimplified, as anti-swelling and anti-freezing behaviors may also be affected by water state, osmotic pressure, ion leaching, solvent exchange, network charge density, and polymer-water interactions.

Answer 2: Thanks for the reviewer's suggestion. We agree that the environmental stability of hydrogels is influenced by multiple interconnected factors, including water state, osmotic pressure, ion leaching, solvent exchange, network charge density, polymer-water interactions, and structural heterogeneity. Our original intention was to highlight the role of heterogeneous structures (*e.g.*, the uneven distribution of interchain interactions and the heterogeneity of phase-separated structures) in environmental instability; however, we acknowledge that this description was overly simplified. In the revised Introduction, we have expanded the discussion to explicitly recognize the multifactorial origins of hydrogel swelling and freezing behavior. These revisions provide a more balanced and comprehensive description of the challenges that hydrogels face in terms of environmental stability.

We revise the content on Page 3 of the revised manuscript.

"The environmental stability of hydrogels is influenced by multiple factors, including water state, osmotic pressure, ion leaching, solvent exchange, network charge density, polymer-water interactions, and structural heterogeneity. These factors make hydrogels susceptible to swelling by external water molecules or freezing of internal water molecules.[34, 35]"

Question 3: Finally, the biocompatibility claims should be moderated. The current data support only preliminary skin-contact compatibility for wearable applications, not broad biomedical safety or biological tissue compatibility.

Answer 3: Thanks for the reviewer's suggestion. We agree that the current biological evaluation is limited to preliminary in vitro cytotoxicity tests and does not justify claims regarding broad biomedical safety or tissue compatibility. Therefore, we have moderated the relevant statements in the manuscript. The revised text now describes the results as preliminary in vitro cytocompatibility evidence. We no longer use broader wording implying comprehensive biological safety or tissue compatibility. We thank the reviewer for helping us improve the accuracy and scope of the manuscript.

We revise the discussion on Page 18 of the revised manuscript.

"Furthermore, cytotoxicity assessments indicate preliminary in vitro cytocompatibility of the hydrogel (Supplementary Fig. 31 and Supplementary Fig. 32)."

Response to Reviewer #3

Comment: The authors have already addressed my comments.

Reply: We are very grateful to the Reviewer for the encouraging comments on our manuscript.
